# Supplementary material for: Identifying modules of cooperating cancer drivers
Source: Mol Syst Biol. 2021 Mar 26;17(3):e9810. doi: 10.15252/msb.20209810 (PMC7995435; doi:10.15252/msb.20209810)
Supplement: Supplementary file 1 — Appendix [file MSB-17-e9810-s015.pdf]

APPENDIX FOR IDENTIFYING MODULES OF COOPERATING CANCER DRIVERS

Michael I. Klein[1,6], Vincent L. Cannataro[2,3], Jeffrey P. Townsend[1,3,4], Scott Newman[6], David F. Stern[4,5†] and Hongyu Zhao [1,3,4†]

[1] Program in Computational Biology and Bioinformatics, Yale University
[2] Department of Biology, Emmanuel College, Boston, MA
[3] Department of Biostatistics, Yale School of Public Health
[4] Yale Cancer Center, Yale University
[5] Department of Pathology, Yale School of Medicine
[6] Bioinformatics R&D, Sema4, Stamford, CT
[†] Equal contributors. Correspondences: df.stern@yale.edu, hongyu.zhao@yale.edu

Contents

Appendix Figures **2**

Appendix Figure S1: Candidate Driver SMGs and SCNVs per Sample . . . 2
Appendix Figure S2: Simulation Example . . . 3
Appendix Figure S3: Core RS Assignment Agreement with Ground Truth . . . 4
Appendix Figure S4: Rule Library Sizes for Simulations . . . 5
Appendix Figure S5: Simulation performance of Fisher's tests under different passenger probabilities . . . 6
Appendix Figure S6: Melanoma Core RS Identification . . . 7
Appendix Figure S7: Melanoma Generalized Cores . . . 8
Appendix Figure S8: Performance and Coverage Convergence for 19 TCGA Cancers . . . 9
Appendix Figure S9: Performance and Coverage Improvements between Phases 2, 3 and 4 . . . 10

Appendix Tables **11**

Appendix Table S1: Mutation Types . . . 11
Appendix Table S2: Default Parameters . . . 12
Appendix Table S3: CRSO Performance on 190 Simulations . . . 13
Appendix Table S4: Phase 1 Performance on 190 Simulations . . . 14
Appendix Table S5: Robustness to Event Exclusion in Melanoma . . . 15
Appendix Table S6: All con-GCRs from 19 TCGA tissues . . . 16
Appendix Table S7: SELECT Common Event Duos Comparison . . . 20

Appendix Figures

Appendix Figure S1: Candidate Driver SMGs and SCNVs per Sample

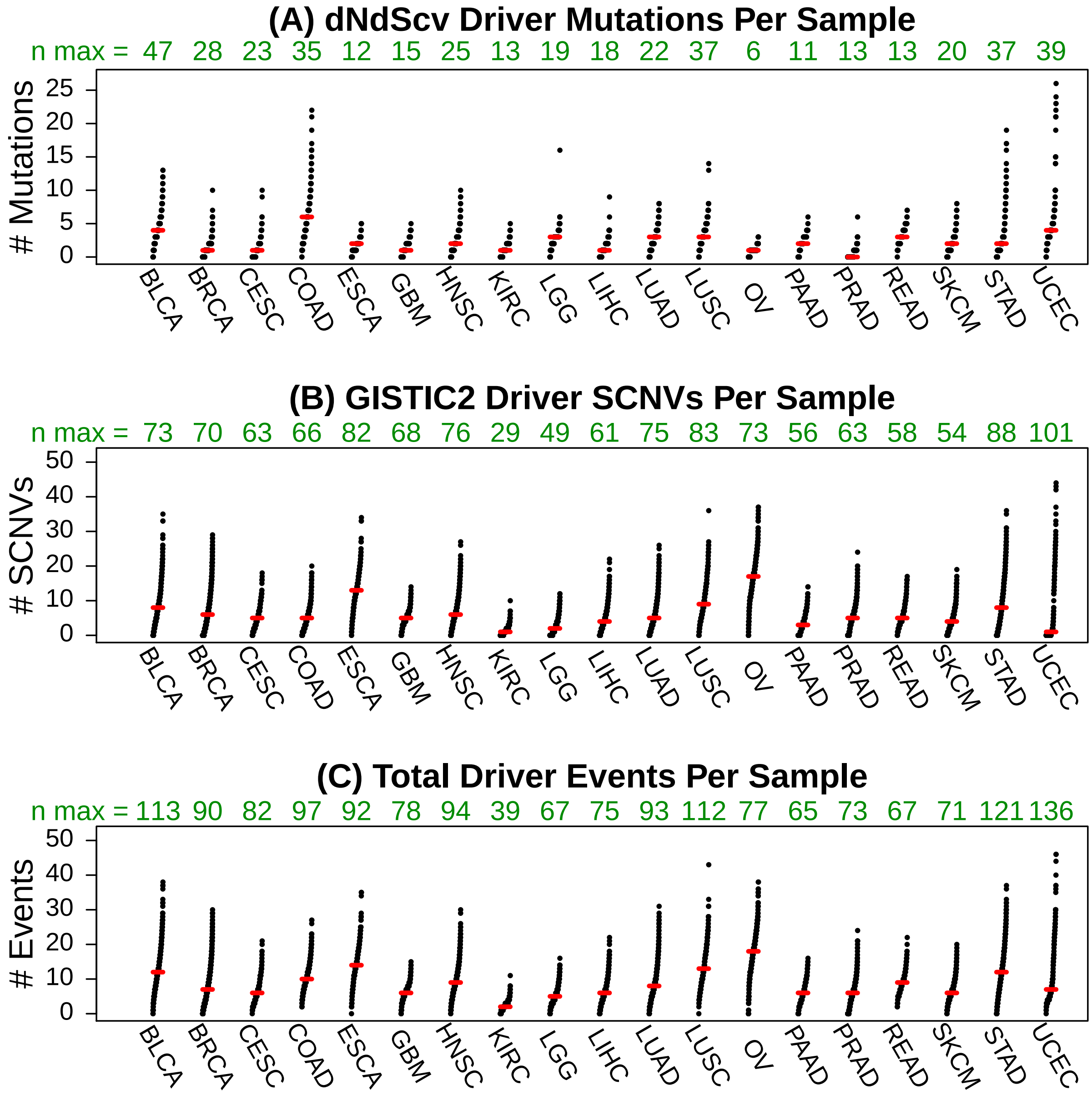

Figure 1: Number of driver events per patients across 19 TCGA cancer types. Panel (A) shows SMGs identified by dNdScv, panel (B) shows SCNVs identified by GISTIC2. Green numbers above each plot show the total number of candidate drivers identified by dNdScv (A) and GISTIC2 (B). Panel (C) shows total driver events from both methods. The number of total drivers per cancer type was less than the sum of the SCNVs and SMGs because some SCNVs and SMGs were combined into hybrid events. Red bars indicate median alterations per sample.

Appendix Figure S2: Simulation Example

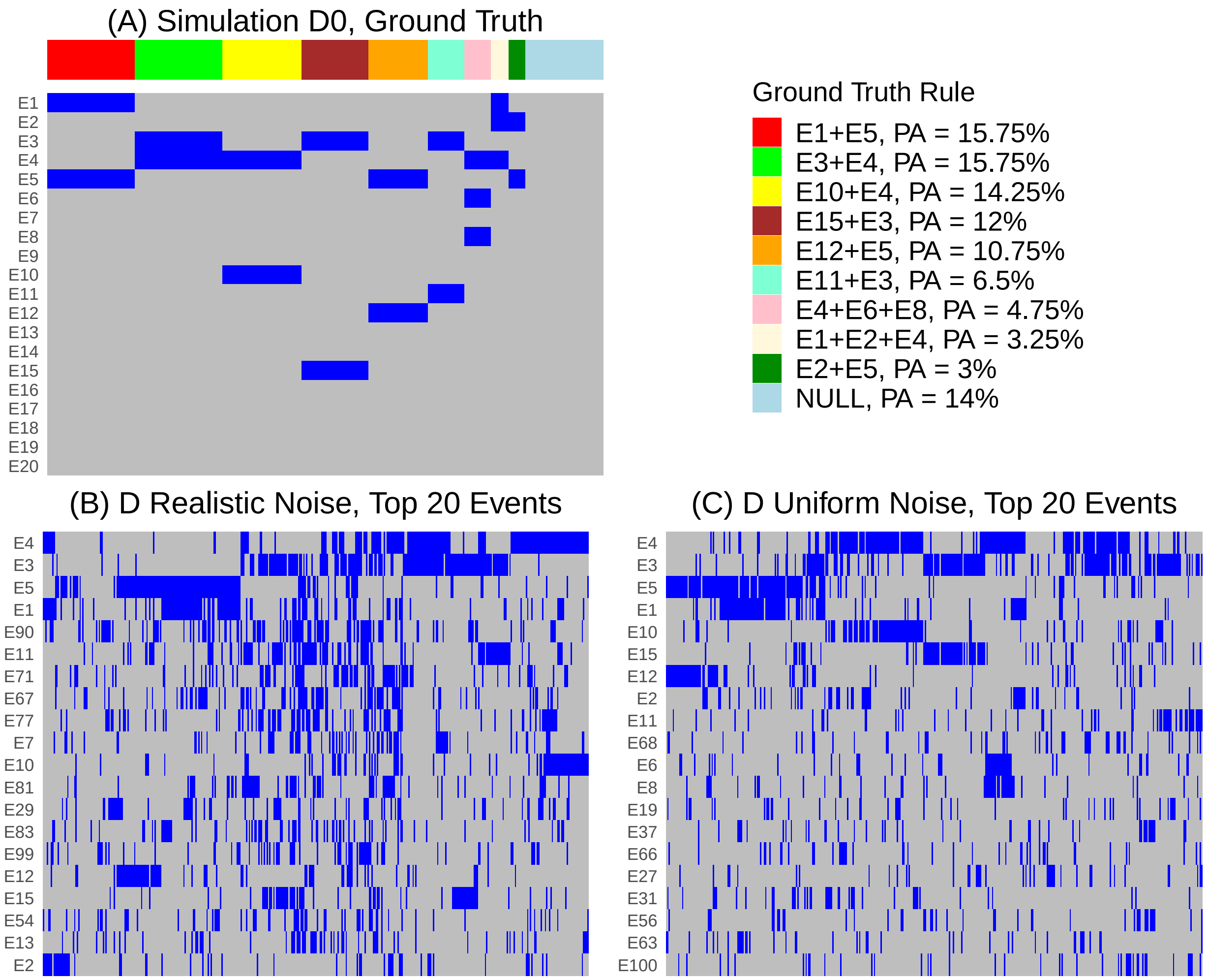

Figure 2: Example of randomly generated simulation with known ground truth of 9 rules. The simulation dataset consists of 400 samples and 100 events (top 20 most frequent events shown). (A) D0 matrix of ground truth driver events. . Legend to the right shows the rules along with percentage of samples assigned (PA). (B) Addition of empirical noise based pooled passenger event rates and sample passenger adjustment factors from 19 TCGA tissue types. (C) Addition of uniform noise equal to the mean of the realistic noise, approximately 8% uniform passenger probability.

Appendix Figure S3: Core RS Assignment Agreement with Ground Truth

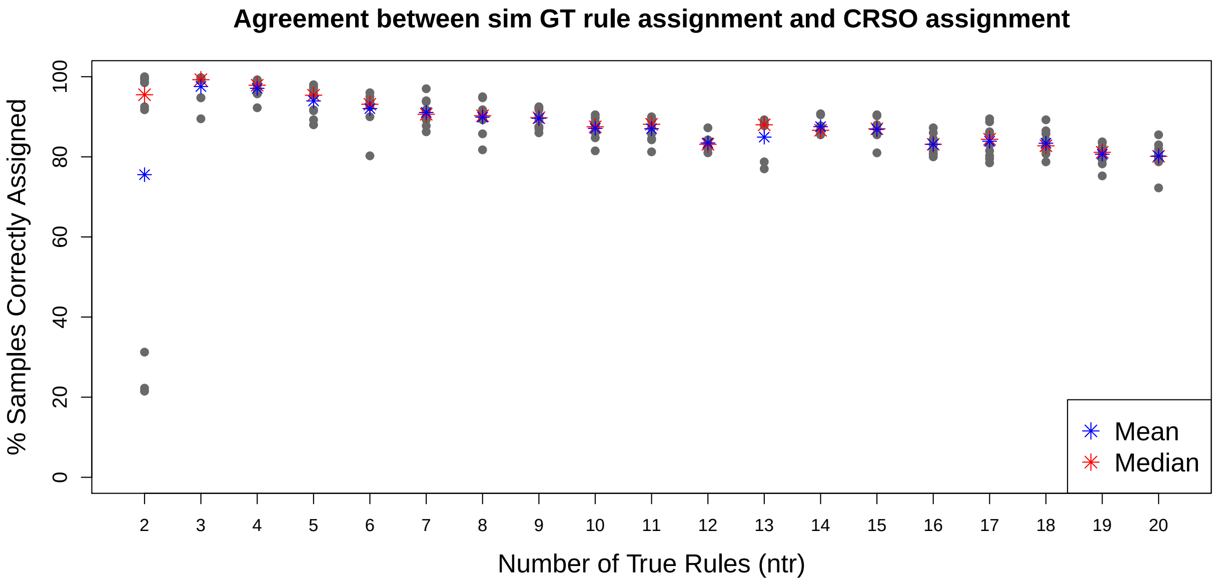

Figure 3: Assignment accuracy of each simulation. CRSO assignments were determined according to the core rule set. Blue/red stars show mean/median accuracy for each ntr value.

Appendix Figure S4: Rule Library Sizes for Simulations

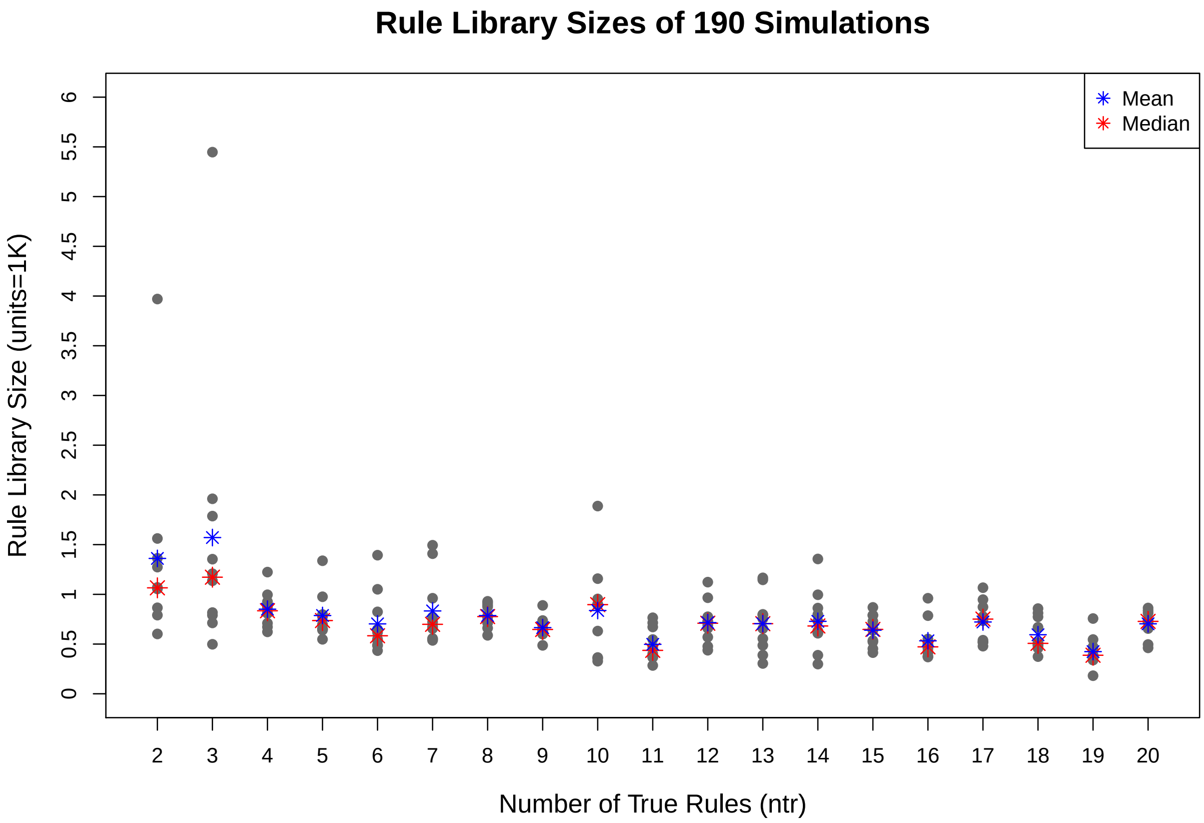

Figure 4: Rule library sizes for the 190 simulations, broken down according to number of true rules (ntr). The largest median library sizes were observed for $ntr = 3(1172.5)$ and $ntr = 2(1065.5)$. For $ntr \in \{4 \ldots 20\}$ the median rule library size ranged from 387.5 ($ntr = 19$) to 896.5 ($ntr = 10$).

Appendix Figure S5: Simulation performance of Fisher's tests under different passenger probabilities

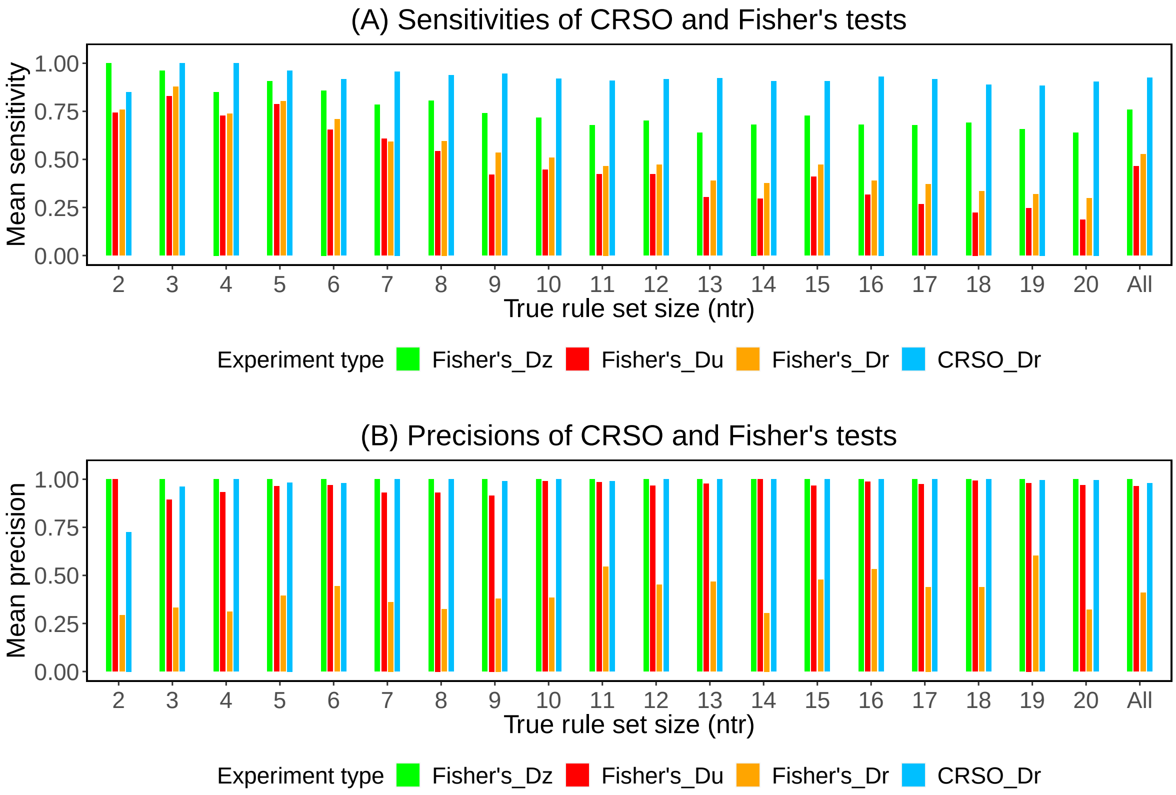

Figure 5: Mean sensitivities and precisions of consensus GCRs grouped by true RS size.

Appendix Figure S6: Melanoma Core RS Identification

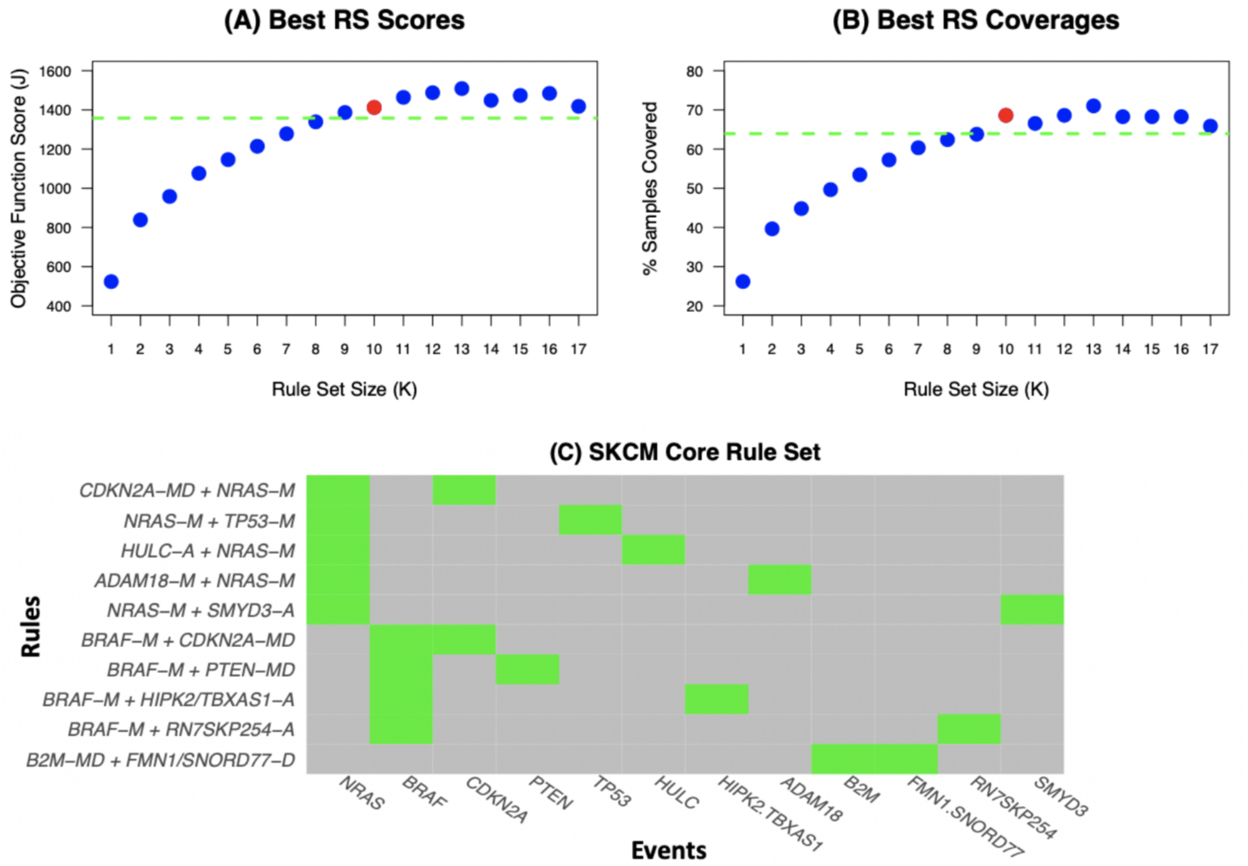

Figure 6: Melanoma CRSO results. A–B) The objective function score (A) and coverage (B) of the best filtered rule sets. The core rule set corresponds to $K = 10$ and is shown in red. The dashed green lines are the thresholds for determining the core rule set. The maximum K shown is the largest K for which valid rule sets that satisfy minimum sample assignment of 9 samples (3%). C) Composition of the core rule set.

Appendix Figure S7: Melanoma Generalized Cores

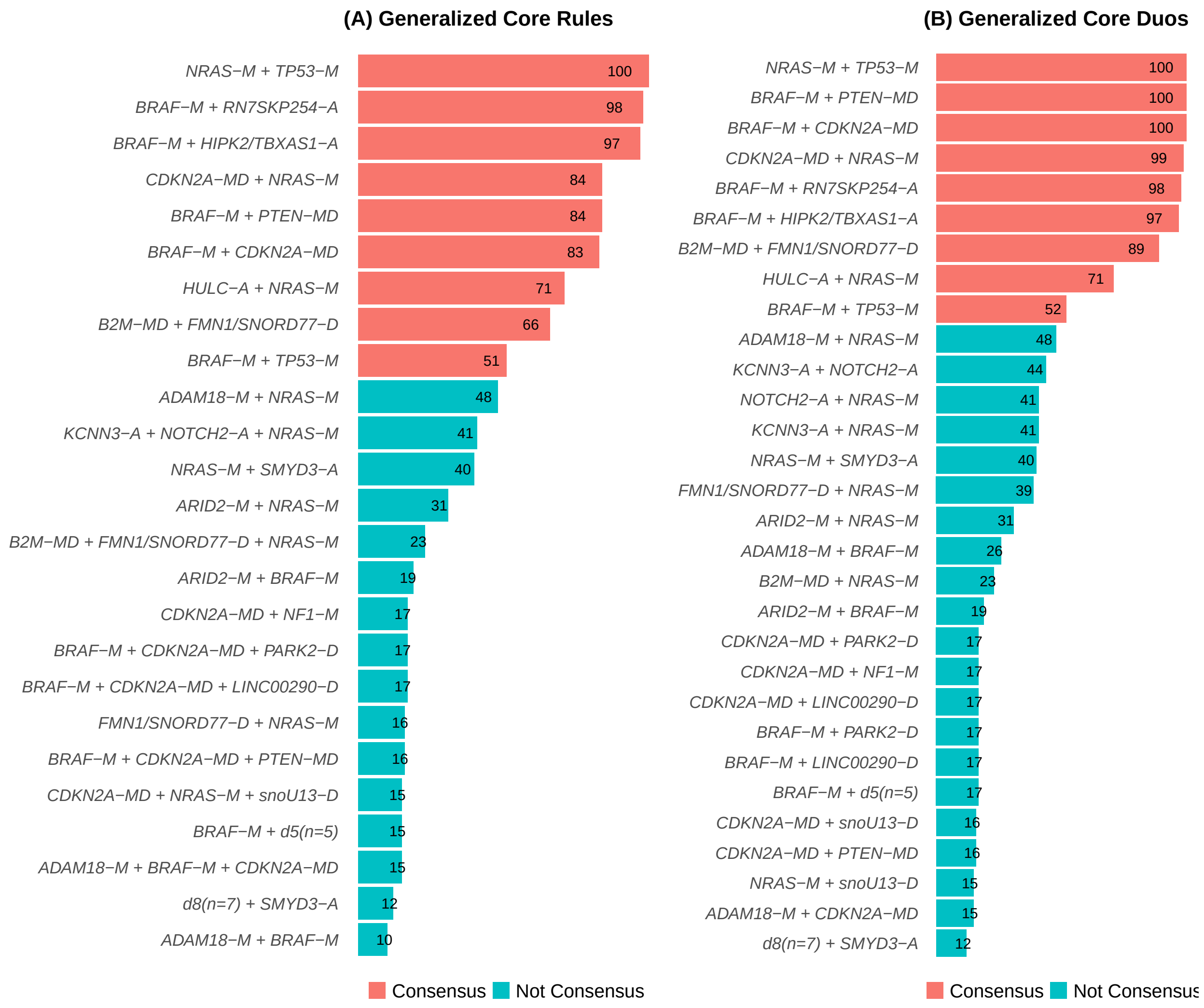

Figure 7: Summary of generalized core (GC) results for melanoma. Bars show confidence levels, which are the percentage of sub-sample iterations containing the observation. Generalized core rules (A) and generalized core duos (B) that achieve a minimum confidence level of 5 are shown.

Appendix Figure S8: Performance and Coverage Convergence for 19 TCGA Cancers

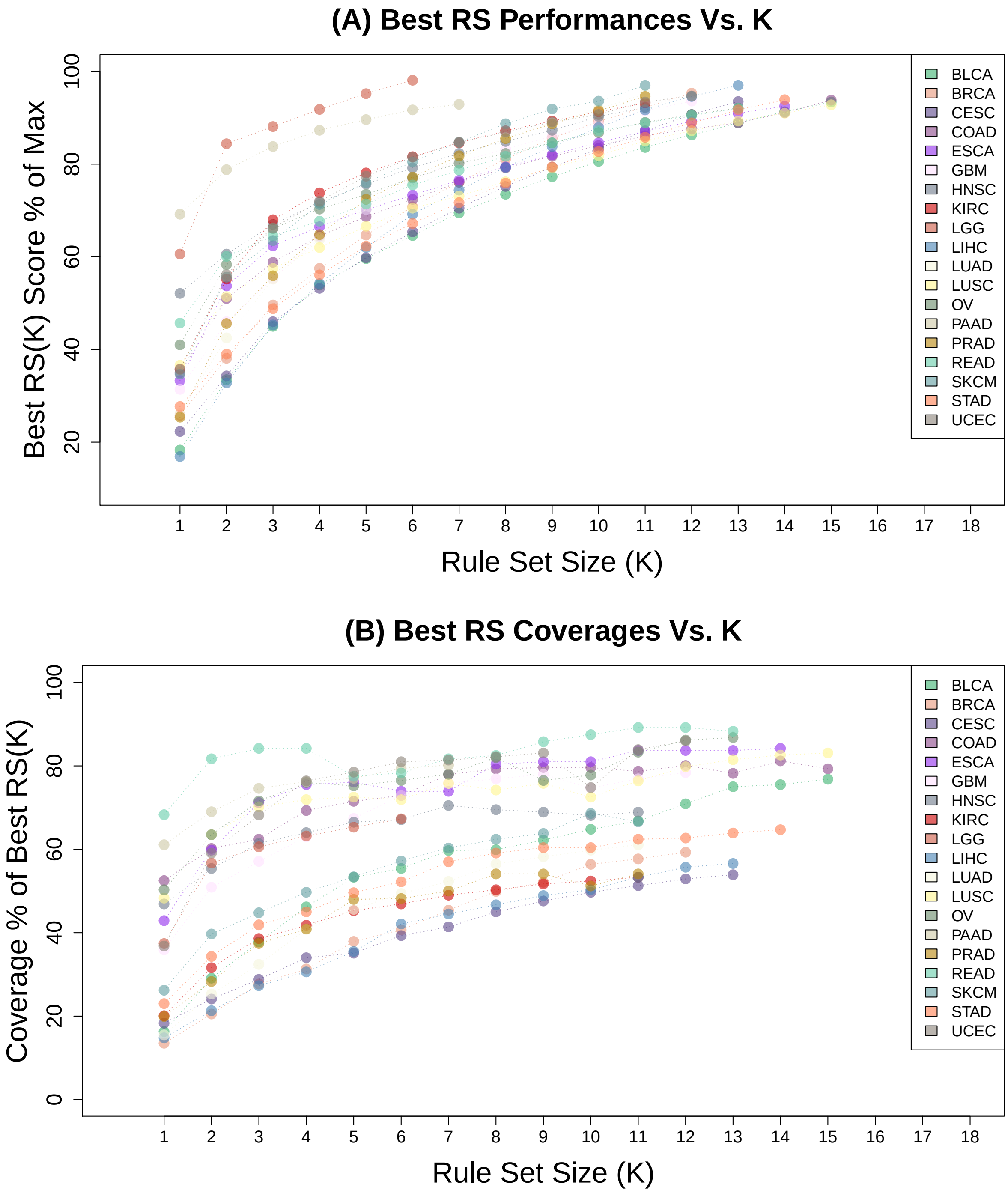

Figure 8: Performance and Coverage Convergence for Different Cancers. For each cancer the maximum K plotted is 2 more than K_{core}.

Appendix Figure S9: Performance and Coverage Improvements between Phases 2, 3 and 4

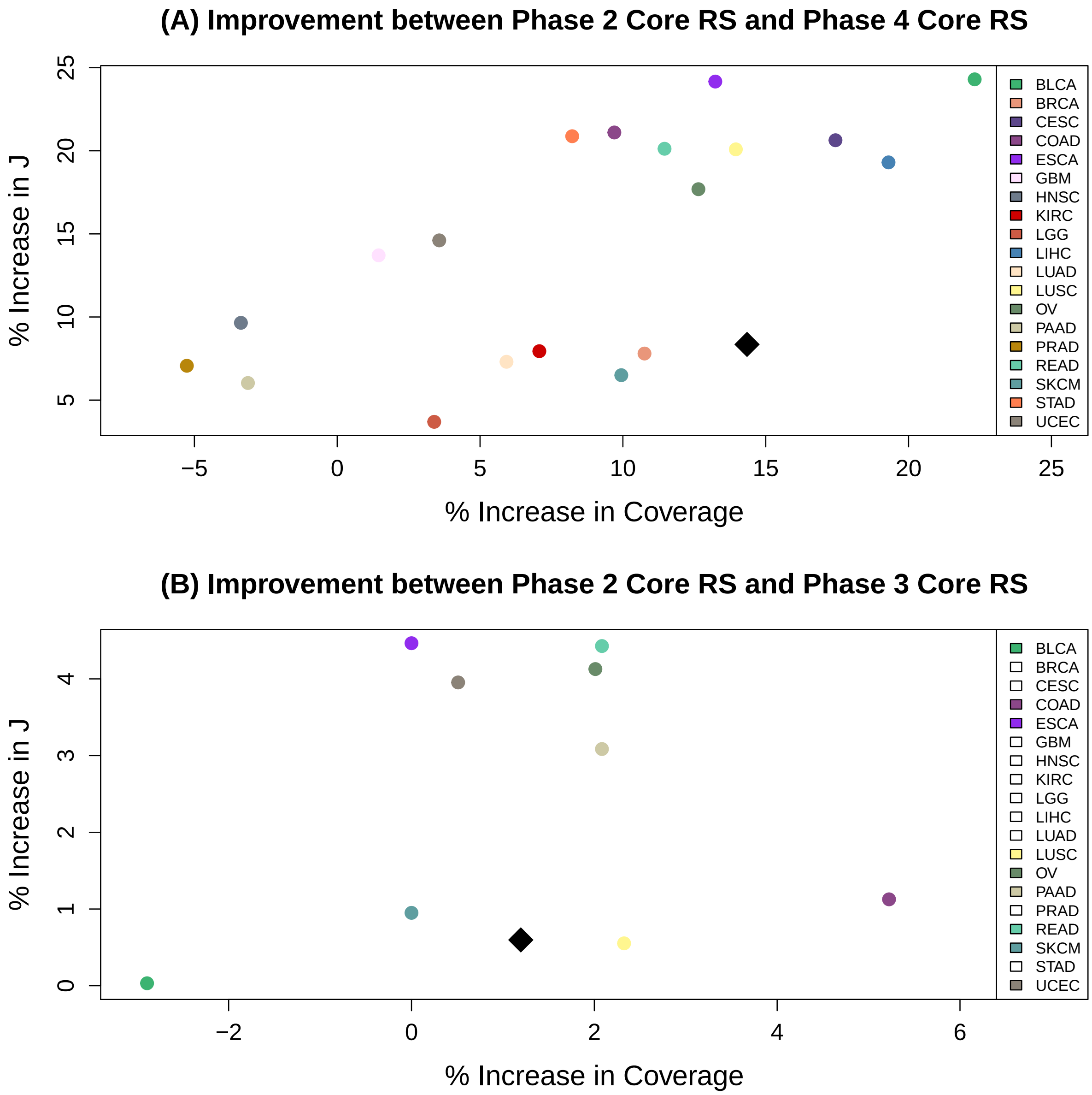

Figure 9: A) Comparison of J and coverage of the core RS post phase 2 with the core RS post phase 4 (i.e., the final core RS) for 19 TCGA cancer types. The mean increases in J and coverage from phase 2 to phase 4 were 14% and 8.3%, respectively (black diamond). B) Comparison of J and coverage of the core RS post phase 2 with the core RS post phase 3 for 19 TCGA cancer types. The mean increases in J and coverage from phase 2 to phase 3 were 1.2% and 0.6%, respectively (black diamond). The core RS were identical post phase 3 and post phase 2 in 10/19 cancer types, labeled in white in panel B.

Appendix Tables

Appendix Table S1: Mutation Types

Table 1: Distribution of mutation types observed across 19 TCGA cancers

Mutation Type	Percentage of All Mutations[a]
Missense_Mutation	61.3
Silent	24.9
Nonsense_Mutation	4.65
Splice_Site	3.08
Frame_Shift_Del	2.85
Frame_Shift_Ins	0.941
Intron	0.597
In_Frame_Del	0.524
RNA	0.397
IGR	0.308
5'Flank	0.165
3'UTR	0.0797
Nonstop_Mutation	0.0677
In_Frame_Ins	0.0655
5'UTR	0.0594
De_novo_Start_OutOfFrame	0.0124
Start_Codon_Del	0.0118
De_novo_Start_InFrame	0.0114
Stop_Codon_Del	0.00515
Start_Codon_Ins	0.00287
Stop_Codon_Ins	0.0015

[a] Mutations occurring in SMGs were excluded.

Appendix Table S2: Default Parameters

Table 2: Default Parameters

Parameter	Default Value	Description	Adjustable
rule.cov.thresh	3% (minimum 6 samples)	Min rule coverage	yes, as desired
msa	3% (minimum 6 samples)	Min samples assigned for every rule in RS	yes, as desired
max.lib.size	2000	Max # rules in rule library	yes, as desired
p1.ss.vec	depends on # rules $(n_r)^a$	Sample sizes per P1 random sampling iterations	no
p1.spr	40	Random sets per rule in P1, for each ss.vec	yes, recommend $\geq$ 10
p1.cut.size	25%	Rules eliminated in each P1 iteration	yes, recommend $\leq$ 50%
p1.stop	24	Stop point for P1	yes, not recommended
K_2	10	Max K considered in P2	yes, recommend $\leq$ 16
p2.mnrs	200,000	Max # RS evaluated for each K in P2	yes, rec. $\geq$ 20,000
p2.max.compute	5*p2.max.nrs	Max # non constrained rule sets considered for evaluation for each K in P2	yes, rec. $\geq$ 2*p2.mnrs
p3.mnrs	100,000	Max # RS considered for each K in P3	yes, rec. $\geq$ 20,000
K_4	40	Max K considered in P4	yes, must be $\geq K_2$ + 1
p4.mnrs	100,000	Max # RS considered for each K in P4	yes, rec. $\geq$ 20,000
core.cov.thresh	90%	Coverage (relative to max) criteria for choosing core RS	yes, recommend $\geq$ 85%
core.perf.thresh	90%	Performance (relative to max) criteria for choosing core RS	yes, recommend $\geq$ 85%
gc.iter	100	subsampling iterations for finding generalized cores	yes, recommend $\geq$ 10
gc.eval	100	Rule sets evaluated per K in each GC iteration	yes, recommend $\geq$ 10
gc.sample.dist	uniform(67,85)	% sub sampled per GC iteration	no
gc.cov.thresh	uniform(85,99)	core coverage thresh per GC iteration	no
gc.perf.thresh	uniform(85,99)	core perf.thresh per GC iteration	no

[a] $ss.vec = [15, 25, 35]$ when $n_r > 300$, $ss.vec = [8, 12, 16]$ when $n_r \in [150, 300)$, $ss.vec = [6, 8, 10, 12]$ when $n_r \leq 150$

Appendix Table S3: CRSO Performance on 190 Simulations

Table 3: Summary of CRSO Performance on Ground Truth Simulations

Number True Rules	Mean Sensitivity	Mean Precision	Mean Accuracy	Median Accuracy
2	0.85	0.73	0.76	0.96
3	1	0.96	0.98	0.99
4	1	1	0.97	0.98
5	0.96	0.98	0.94	0.95
6	0.92	0.98	0.92	0.93
7	0.96	1	0.91	0.91
8	0.94	1	0.9	0.9
9	0.94	0.99	0.9	0.9
10	0.92	1	0.87	0.88
11	0.91	0.99	0.87	0.88
12	0.92	1	0.84	0.83
13	0.92	1	0.86	0.88
14	0.91	1	0.87	0.86
15	0.91	1	0.85	0.86
16	0.93	1	0.83	0.83
17	0.92	1	0.84	0.84
18	0.89	1	0.83	0.83
19	0.88	0.99	0.81	0.81
20	0.9	0.99	0.8	0.8
All	0.93	0.98	0.87	0.88

Ten simulations were performed for each ground truth rule set size. Mean sensitivity is the mean fraction of true rules identified among the con-GCRs. Mean precision is the mean fraction of con-GCRs that are part of the ground truth rule set. Simulation accuracy is the fraction of samples assigned assigned correctly by CRSO, using the core RS. The last row shows the mean/median values over all 190 simulations

Appendix Table S4: Phase 1 Performance on 190 Simulations

Table 4: Phase 1 Performance on Ground Truth Simulations

NTR	P1_Score	P1_Top_10	P1_Top_20	P1_Top_30	P1_Top_40	Cov_Top_40	SJ_Top_40
2	0.99	1	1	1	1	0.9	0.85
3	0.98	1	1	1	1	0.9	0.7
4	0.98	1	1	1	1	0.78	0.7
5	0.96	0.98	1	1	1	0.88	0.72
6	0.97	0.97	1	1	1	0.85	0.78
7	0.98	0.96	1	1	1	0.71	0.69
8	0.96	0.91	0.99	0.99	1	0.75	0.62
9	0.96	0.89	1	1	1	0.72	0.69
10	0.94	0.85	0.97	0.98	0.99	0.65	0.6
11	0.97	0.83	0.99	1	1	0.74	0.66
12	0.97	0.77	1	1	1	0.69	0.64
13	0.97	0.71	1	1	1	0.57	0.5
14	0.97	0.66	0.98	1	1	0.56	0.49
15	0.95	0.6	0.93	0.99	1	0.44	0.39
16	0.94	0.56	0.95	1	1	0.51	0.49
17	0.94	0.54	0.91	0.99	0.99	0.36	0.38
18	0.94	0.54	0.91	0.98	0.99	0.38	0.38
19	0.97	0.51	0.94	0.99	1	0.49	0.49
20	0.94	0.46	0.86	0.98	0.99	0.28	0.26
All	0.96	0.78	0.97	0.99	1	0.64	0.58

Mean performance over ten iterations of ground truth rule set size (NTR). P1_Score is the mean phase 1 score, which compares the positions of ground truth rules in phase 1 rankings to the theoretical maximum ranking. P1_Top_X indicates the mean fraction of ground truth rules identified in the top X phase 1 rules. Nearly 100 percent of ground truth rules are identified within the top 30 rules, for all ntr between 2 and 20. Cov_Top_40 is the mean fraction of rules within the top 40 rules ranked according to coverage. SJ_Top_40 is the mean fraction of rules within the top 40 rules ranked according to single rule objective function score (i.e, SJ for Single rule J score). Phase 1 importance rankings are much better at prioritizing ground truth rules compared to either coverage or SJ rankings. Last row indicates mean over all 190 simulations.

Appendix Table S5: Robustness to Event Exclusion in Melanoma

Table 5: Robustness of CRSO GCDs to Event Exclusion in Melanoma

Excluded E	E Freq %	FR Incl E %	WFR Incl E %	FR Retention %	WFR Retention %	FPR %	WFPR %
BRAF-M	50	35	40.5	94.3	98	31.4	9.53
CDKN2A-MD	46.2	25	22.4	83.9	96.3	19.4	19.9
NRAS-M	30	27.5	40.6	90.2	95.5	39	27.7
ADAM18-M	18.6	10	6.66	92.3	99.8	15.4	4.51
snoU13-D	17.6	7.5	2.37	77.4	98.3	3.23	0.667
TP53-M	16.6	7.5	11.3	92.3	96	12.8	1.34
PTEN-MD	16.6	5	8.59	87.2	93.7	15.4	9.1
SMYD3-A	15.5	10	4.29	62.1	93.2	13.8	3.48
NOTCH2-A	15.5	7.5	6.59	91.7	96.5	5.56	0.657
RPTOR-A	14.5	0	0	77.1	98.3	8.57	0.239
d7(n=3)	13.8	5	9.47	74.3	94.3	17.1	6.03
B2M-MD	13.4	7.5	8.51	85.7	98.5	8.57	1.75
NF1-M	13.1	2.5	1.26	95	97.7	7.5	1.19
HULC-A	13.1	2.5	5.26	81.1	94.7	13.5	2.19
TERT-A	13.1	0	0	82.9	98.5	2.86	0.961

FR Full results (rules or duos), i.e., results obtained using all inputs. **FR Inclusion E** is the percentage of FR that contain E. **WFR Inclusion E** is the sum of confidences of FR that contain E/ sum of confidences of FR. **FR Retention** is the percentage of eligible FR were retained among the new results. **WFR Retention** is the sum of confidences of retainted eligible FR / sum of confidences of eligible FR. **FPR** is the percentage of new results that were not in the full results (i.e., emurgent). **WFPR** is the sum of confidences of emurgent rules / sum of confidences all rules.

Appendix Table S6: All con-GCRs from 19 TCGA tissues

Table 6: Consensus GCRs from 19 TCGA Cancer Types

Tissue	Rule	P1 Rank	Conf.	Cov.	SJ	NE
BLCA	*FGFR3-MA + KDM6A-MD*	5	100	10.7% (r=42)	332 (r=12)	2
BLCA	*ERBB2-MA + TP53-M*	6	100	12.8% (r=22)	330 (r=10)	2
BLCA	*CDKN2A-MD + FGFR3-MA*	1	99	11.2% (r=33)	388 (r=17)	2
BLCA	*RB1-M + TP53-M*	4	97	13.8% (r=15)	336 (r=3)	2
BLCA	*ARID1A-MD + PIK3CA-M*	11	97	8.16% (r=99)	292 (r=47)	2
BLCA	*a1(n=4) + ENSA/SNORA40-A + TP53-M*	12	96	12.8% (r=23)	287 (r=8)	3
BLCA	*PIK3CA-M + TP53-M*	3	89	11.7% (r=28)	343 (r=6)	2
BLCA	*CDKN2A-MD + KDM6A-MD*	7	89	15.1% (r=11)	319 (r=15)	2
BLCA	*CDKN2A-MD + TP53-M*	2	88	16.3% (r=6)	382 (r=1)	2
BLCA	*ERBB4/RNA5SP119-D + KCNJ13-D*	15	81	19.1% (r=1)	278 (r=28)	2
BLCA	*ARID1A-MD + SOX4-A + TP53-M*	37	79	7.14% (r=172)	204 (r=46)	3
BLCA	*CASC8-A + YWHAZ-A*	61	79	13.3% (r=17)	176 (r=99)	2
BLCA	*ELF3-M + KDM6A-MD + TP53-M*	104	53	3.57% (r=1393)	152 (r=199)	3
BLCA	*CDKN2A-MD + NFE2L2-M*	26	51	5.61% (r=337)	220 (r=131)	2
BLCA	*ARID1A-MD + CDKN2A-MD*	10	57	13.3% (r=18)	300 (r=27)	2
BRCA	*MAP3K1-M + PIK3CA-MA*	4	100	3.74% (r=245)	478 (r=49)	2
BRCA	*AQP11/CLNS1A-A + CCND1/ORAOV1-A*	6	100	12.6% (r=3)	457 (r=7)	2
BRCA	*IKBKB/POLB-A + ZNF703-A*	10	100	8.93% (r=10)	424 (r=36)	2
BRCA	*CDH1-M + PIK3CA-MA*	2	99	5.61% (r=71)	676 (r=10)	2
BRCA	*PTEN-MD + TP53-M*	7	99	6.96% (r=32)	448 (r=17)	2
BRCA	*ERBB2-MA + VMP1-A*	11	95	9.35% (r=7)	420 (r=28)	2
BRCA	*d2(snoU13)-D + THYN1-D*	12	94	12.3% (r=4)	414 (r=31)	2
BRCA	*ERBB2-MA + TP53-M*	8	83	8.41% (r=14)	446 (r=4)	2
BRCA	*PIK3CA-MA + TP53-M*	1	72	13.5% (r=1)	911 (r=1)	2
BRCA	*CCND1/ORAOV1-A + PIK3CA-MA*	3	66	11.3% (r=5)	627 (r=3)	2
BRCA	*MYC-A + TP53-M*	5	62	12.8% (r=2)	462 (r=2)	2
BRCA	*GATA3-M + VMP1-A*	23	54	3.43% (r=307)	344 (r=174)	2
BRCA	*FBLN5-D + ZFP36L1-MD*	41	52	5.61% (r=68)	300 (r=178)	2
CESC	*NTM/RNU6ATAC12P-D + TMEM136-D*	1	100	18.3% (r=1)	131 (r=1)	2
CESC	*PIK3CA-M + PTEN-MD*	2	100	5.24% (r=32)	113 (r=8)	2
CESC	*LINC00393-A + RASA3-A*	10	100	5.76% (r=20)	69.4 (r=30)	2
CESC	*a11(n=6) + ZNF750-MD*	37	89	3.66% (r=66)	43.1 (r=78)	2
CESC	*FNDC3B/RN7SL141P-A + PTEN-MD*	14	84	3.14% (r=159)	59.2 (r=70)	2
CESC	*GCDH/SYCE2-A + STK11-MD*	18	84	3.66% (r=68)	53.4 (r=84)	2
CESC	*FAT1-MD + FNDC3B/RN7SL141P-A*	15	78	5.24% (r=25)	58.7 (r=53)	2
CESC	*FNDC3B/RN7SL141P-A + KCNJ13/RN7SL359P-D*	34	78	6.81% (r=14)	44 (r=34)	2
CESC	*a6(n=5) + PIK3CA-M*	5	91	6.28% (r=18)	77.1 (r=7)	2
CESC	*BCL2L1/COX4I2-A + PIK3CA-M*	7	77	3.66% (r=100)	73.5 (r=27)	2
CESC	*FGF3-A + LRP1B-D*	52	75	3.66% (r=74)	37.7 (r=97)	2
CESC	*EP300-M + PIK3CA-M*	9	74	4.71% (r=42)	71.6 (r=15)	2
CESC	*a6(n=5) + FBXW7-M*	16	74	3.14% (r=111)	58.1 (r=48)	2
CESC	*HLA-B-M + PIK3CA-M*	33	72	3.66% (r=97)	44.3 (r=38)	2
CESC	*a2(n=6) + KCNJ13/RN7SL359P-D + TMEM136-D*	57	71	5.24% (r=30)	36.6 (r=14)	3
COAD	*APC-M + PTEN-M + SMAD4-MD*	34	97	20.4% (r=33)	711 (r=28)	3
COAD	*ATM-M + CTNNB1-M + KRAS-M + PIK3CA-M + PTEN-M + TP53-M*	147	92	4.7% (r=1074)	443 (r=293)	6
COAD	*BRAF-M + PIH1/WWOX-D + RBFOX1-D*	6	81	5.52% (r=735)	1030 (r=817)	3
COAD	*APC-M + ATM-M + PIK3CA-M + PTEN-M*	16	78	16.9% (r=51)	825 (r=22)	4
COAD	*ATM-M + PIK3CA-M + SMAD4-MD + TP53-M*	87	64	11.3% (r=151)	512 (r=68)	4
COAD	*RBFOX1-D + TP53-M*	8	60	25.4% (r=18)	999 (r=67)	2
COAD	*APC-M + TP53-M*	1	56	52.5% (r=1)	1330 (r=1)	2
COAD	*BRAF-M + PIK3CA-M + PTEN-M + SMAD4-MD + TP53-M*	196	56	5.25% (r=847)	403 (r=342)	5
COAD	*APC-M + KRAS-M + PIK3CA-M*	7	53	24.3% (r=20)	1010 (r=7)	3
COAD	*APC-M + d2(n=4)*	37	51	23.5% (r=22)	697 (r=108)	2
ESCA	*MYC-A + TP53-M*	6	100	33.2% (r=5)	340 (r=5)	2
ESCA	*GMDS-D + PIH1/WWOX-D + TP53-M*	13	99	18.5% (r=42)	276 (r=19)	3
ESCA	*SMAD4-MD + TP53-M*	15	94	19% (r=36)	272 (r=35)	2
ESCA	*CDKN2A-D + FGF3-A + TP53-M*	2	91	26.6% (r=10)	428 (r=4)	3

ESCA	*ACTRT3/MYNN-A + FGF3-A + IMMP2L/LRRN3-D +PTPRN2-D + TP53-M*	17	87	7.07% (r=797)	261 (r=157)	5
ESCA	*MIR5707/MIR595/PTPRN2-D + PIH1/WWOX-D*	140	84	16.3% (r=53)	139 (r=486)	2
ESCA	*TP53-M + ZNF750-MD*	23	71	13.6% (r=102)	242 (r=108)	2
ESCA	*LRP1B-D + TP53-M*	7	65	32.6% (r=6)	336 (r=7)	2
ESCA	*ACTRT3/MYNN-A + NFE2L2-M + TP53-M*	71	59	5.98% (r=1280)	169 (r=345)	3
ESCA	*d9(n=6) + FHIT/NPCDR1/U3-D + IMMP2L/LRRN3-D + PIH1/WWOX-D + TP53-M*	112	51	5.98% (r=1378)	149 (r=260)	5
GBM	*CDKN2A-D + PTEN-MD*	1	100	27.8% (r=2)	557 (r=2)	2
GBM	*CDK4/MARCH9/TSPAN31-A + CPM/MDM2-A*	9	99	7.69% (r=29)	256 (r=47)	2
GBM	*CDK4/MARCH9/TSPAN31-A + TP53-M*	6	89	6.96% (r=37)	304 (r=36)	2
GBM	*CDKN2A-D + NF1-MD*	12	82	9.89% (r=20)	212 (r=24)	2
GBM	*CDKN2A-D + EGFR-M + SNORA73-A*	3	77	14.7% (r=8)	395 (r=4)	3
GBM	*PTEN-MD + TP53-M*	5	77	12.1% (r=13)	308 (r=9)	2
GBM	*CDKN2A-D + PIK3R1-M*	15	70	8.06% (r=27)	200 (r=30)	2
GBM	*ATRX-M + IDH1-M + TP53-M*	11	63	3.66% (r=131)	221 (r=43)	3
GBM	*CDKN2A-D + PDGFRA-MA*	8	59	11% (r=15)	269 (r=16)	2
GBM	*EGFR-M + PTEN-MD + SNORA73-A*	30	52	6.96% (r=40)	143 (r=14)	3
HNSC	*FAT1-MD + TP53-M*	4	98	27.1% (r=3)	827 (r=5)	2
HNSC	*CDKN2A-MD + FAT1-MD + NOTCH1-MD*	25	74	8.51% (r=83)	400 (r=45)	3
HNSC	*CASP8-M + HRAS-M*	8	73	3.76% (r=549)	621 (r=302)	2
HNSC	*RN7SKP265-A + TP53-M*	7	69	26.9% (r=4)	671 (r=6)	2
HNSC	*NOTCH1-MD + TP53-M*	13	64	15.4% (r=15)	516 (r=16)	2
HNSC	*CDKN2A-MD + PPFIA1-A + TP53-M*	2	52	21.2% (r=8)	892 (r=2)	3
HNSC	*CDKN2A-MD + NFE2L2-MA + TP53-M*	24	50	8.71% (r=77)	406 (r=35)	3
KIRC	*PBRM1-M + VHL-MD*	1	100	20.1% (r=1)	615 (r=1)	2
KIRC	*RNU6ATAC4P-D + VHL-MD*	2	100	15% (r=3)	475 (r=3)	2
KIRC	*RNA5SP200-A + VHL-MD*	3	100	16.6% (r=2)	459 (r=2)	2
KIRC	*ARID1A-MD + VHL-MD*	4	100	7.16% (r=5)	205 (r=5)	2
KIRC	*CAHM/QKI-D + PARK2-D*	6	100	4.39% (r=12)	190 (r=17)	2
KIRC	*BAP1-M + VHL-MD*	5	98	5.54% (r=6)	201 (r=8)	2
KIRC	*PBRM1-M + RNA5SP200-A*	9	97	7.16% (r=4)	166 (r=10)	2
KIRC	*MTOR-M + VHL-MD*	8	76	3.93% (r=15)	169 (r=11)	2
KIRC	*NRXN3-D + VHL-MD*	15	74	2.54% (r=22)	98.4 (r=19)	2
KIRC	*PBRM1-M + SETD2-M*	17	65	4.39% (r=13)	95.3 (r=13)	2
KIRC	*BAP1-M + RNA5SP200-A*	18	50	3% (r=18)	88.4 (r=26)	2
LGG	*CIC-M + IDH1-M*	1	100	19.3% (r=5)	2610 (r=5)	2
LGG	*EGFR-M + SNORA73-A*	10	86	3.9% (r=132)	655 (r=153)	2
LGG	*IDH1-M + PIK3CA-M*	8	84	6.04% (r=61)	695 (r=42)	2
LGG	*IDH1-M + ISOC2/NAT14/ZNF628-D + TP53-M*	13	82	10.3% (r=15)	591 (r=9)	3
LGG	*ATRX-MD + IDH1-M + TP53-M*	2	71	37.4% (r=4)	2520 (r=1)	3
LGG	*FUBP1-M + IDH1-M*	6	59	8.77% (r=21)	889 (r=21)	2
LIHC	*ARID1A-MD + MIR4689-D*	1	100	14.8% (r=1)	209 (r=1)	2
LIHC	*ALB-M + CTNNB1-M*	4	98	4.37% (r=48)	177 (r=12)	2
LIHC	*CTSS-A + TP53-MD*	5	98	7.92% (r=5)	176 (r=7)	2
LIHC	*LINC00676-A + PCCA-A*	12	98	13.4% (r=2)	134 (r=5)	2
LIHC	*CTSS-A + RB1-MD*	16	94	6.83% (r=6)	124 (r=23)	2
LIHC	*ARID1A-MD + CTNNB1-M*	7	91	6.28% (r=15)	162 (r=8)	2
LIHC	*CTNNB1-M + RN7SKP226-A*	11	86	6.28% (r=14)	135 (r=9)	2
LIHC	*RN7SKP226-A + TP53-MD*	6	85	8.74% (r=3)	166 (r=4)	2
LIHC	*ALB-M + TP53-MD*	15	82	3.55% (r=86)	128 (r=27)	2
LIHC	*RN7SKP96-D + TACR3-D*	29	82	4.64% (r=31)	92.8 (r=77)	2
LIHC	*RB1-MD + TP53-MD*	2	50	8.47% (r=4)	201 (r=2)	2
LIHC	*C19orf77/NFIC-D + TP53-MD*	9	80	6.83% (r=7)	143 (r=10)	2
LIHC	*CCND1/ORAOV1-A + TP53-MD*	8	80	4.64% (r=38)	152 (r=17)	2
LIHC	*CTNNB1-M + RB1-MD*	20	53	4.37% (r=49)	112 (r=21)	2
LIHC	*LRP1B-D + TP53-MD*	19	52	5.19% (r=26)	112 (r=24)	2
LIHC	*PTEN-MD + TP53-MD*	25	50	4.64% (r=37)	98.3 (r=22)	2
LUAD	*SFTA3-A + TP53-M*	5	97	15.5% (r=1)	362 (r=5)	2
LUAD	*CDKN2A-MD + KRAS-MA*	7	97	8.16% (r=16)	307 (r=10)	2
LUAD	*KRAS-MA + SFTA3-A*	4	95	10.7% (r=10)	362 (r=6)	2
LUAD	*CDKN2A-MD + TP53-M*	8	95	13.4% (r=5)	305 (r=4)	2
LUAD	*KRAS-MA + STK11-M*	3	90	10% (r=11)	380 (r=3)	2
LUAD	*EGFR-MA + TP53-M*	2	74	11.5% (r=9)	408 (r=2)	2
LUAD	*ATM-M + KRAS-MA*	6	73	6.07% (r=48)	338 (r=16)	2

LUAD	*KRAS-MA + TP53-M*	1	68	15.5% (r=2)	593 (r=1)	2
LUAD	*CDH10-M + TP53-M*	10	67	13.8% (r=4)	289 (r=8)	2
LUAD	*NF1-M + TP53-M*	11	77	8.79% (r=15)	270 (r=18)	2
LUAD	*BRAF-M + TP53-M*	13	66	5.23% (r=70)	240 (r=41)	2
LUSC	*TP53-M + WHSC1L1-A*	3	100	21.3% (r=16)	299 (r=17)	2
LUSC	*FOXP1/MIR1284-D +ROBO1-D + PROS1/STX19-D + ROBO2-D + TP53-M*	17	97	6.74% (r=456)	193 (r=111)	5
LUSC	*CSMD3-M + NFE2L2-MA + SOX2-A*	30	79	8.43% (r=230)	152 (r=126)	3
LUSC	*RB1-MD + SOX2-A + TP53-M*	10	64	8.43% (r=251)	218 (r=59)	3
LUSC	*PIK3CA-M + SOX2-A + TP53-M*	11	63	8.43% (r=252)	216 (r=64)	3
LUSC	*CDKN2A-MD + EGFR-A + LRP1B-D + SOX2-A + TP53-M*	18	61	6.18% (r=605)	185 (r=84)	5
LUSC	*CERS3-A + TP53-M*	44	55	15.2% (r=37)	135 (r=50)	2
LUSC	*TP53-M + TSPAN4-D*	21	90	15.7% (r=33)	172 (r=35)	2
LUSC	*CDH10-M + TP53-M*	12	68	16.3% (r=30)	212 (r=34)	2
LUSC	*NF1-MD + TP53-M*	16	65	15.7% (r=34)	194 (r=38)	2
OV	*RN7SL501P-D + TP53-M*	5	100	31.4% (r=9)	895 (r=8)	2
OV	*BSPH1-D + RN7SL526P-D + TCF3-D + TP53-M*	6	100	11.9% (r=472)	862 (r=85)	4
OV	*d2(n=15) + FKSG52/PDE4D-D + MECOM-A + MYC-A + TP53-M*	8	99	13.2% (r=339)	717 (r=39)	5
OV	*d15(n=5) + PPP2R2A-D + TP53-M*	15	96	12.5% (r=387)	617 (r=159)	3
OV	*BRD4-A + TP53-M*	11	95	24.2% (r=27)	687 (r=24)	2
OV	*d6(n=6) + MECOM-A + MYC-A + TP53-M*	76	92	12.7% (r=373)	456 (r=108)	4
OV	*MYC-A + TCF3-D + TP53-M*	13	89	30.5% (r=11)	674 (r=5)	3
OV	*CBX8-A + MYC-A*	117	82	25.1% (r=23)	405 (r=389)	2
OV	*MECOM-A + TCF3-D + TP53-M*	9	66	29.2% (r=14)	713 (r=6)	3
OV	*CCNE1-A + RN7SL566P/SAMD4B-A + TP53-M*	31	56	13% (r=349)	539 (r=119)	3
OV	*ANKS1B/FAM71C/RNA5SP366-D + d13(n=19) + TP53-M*	55	52	12.7% (r=367)	488 (r=208)	3
OV	*d2(n=15) + FKSG52/PDE4D-D + TCF3-D*	275	51	18.5% (r=97)	321 (r=432)	3
PAAD	*KRAS-M + TP53-M*	1	95	61.1% (r=1)	754 (r=1)	2
PAAD	*CDKN2A-MD + KRAS-M*	3	67	45.2% (r=2)	518 (r=3)	2
PAAD	*KRAS-M + SMAD4-MD*	4	63	28.6% (r=5)	332 (r=6)	2
PRAD	*FAM92B-D + ZFHX3-D*	2	100	13.8% (r=3)	296 (r=8)	2
PRAD	*RNY1P8-D + ZC3H13-D*	3	93	16.1% (r=2)	282 (r=6)	2
PRAD	*FOXA1-M + ZNF292-D*	9	84	3.86% (r=187)	207 (r=102)	2
PRAD	*CHD1-D + SPOP-M + ZNF292-D*	11	72	6.5% (r=49)	195 (r=3)	3
PRAD	*PTEN-MD + TP53-M*	5	69	5.28% (r=85)	258 (r=36)	2
PRAD	*ERG-D + TMPRSS2-MD*	1	66	19.9% (r=1)	373 (r=1)	2
READ	*APC-MD + INS/MIR4686/TH-A + KRAS-MA*	13	83	5.83% (r=459)	231 (r=240)	3
READ	*APC-MD + KRAS-MA + TP53-M*	3	79	35.8% (r=4)	521 (r=2)	3
READ	*APC-MD + d2(n=4)*	6	74	30.8% (r=6)	278 (r=14)	2
READ	*a1(n=8) + APC-MD + CTNNBL1-A + TP53-M*	16	72	8.33% (r=194)	221 (r=88)	4
READ	*APC-MD + PIK3CA-M + TP53-M*	4	71	25.8% (r=12)	430 (r=5)	3
READ	*APC-MD + d3(n=623) + TP53-M*	10	65	21.7% (r=20)	239 (r=8)	3
READ	*APC-MD + SMAD4-MD*	12	65	21.7% (r=19)	237 (r=28)	2
READ	*KRAS-MA + PIK3CA-M*	56	64	16.7% (r=33)	147 (r=36)	2
READ	*d2(n=4) + KRAS-MA + PARK2-D + TP53-M*	192	55	4.17% (r=1011)	90.9 (r=339)	4
SKCM	*NRAS-M + TP53-M*	4	100	6.55% (r=29)	168 (r=8)	2
SKCM	*BRAF-M + RN7SKP254-A*	11	98	6.55% (r=32)	137 (r=21)	2
SKCM	*BRAF-M + HIPK2/TBXAS1-A*	6	97	7.93% (r=13)	161 (r=10)	2
SKCM	*CDKN2A-MD + NRAS-M*	2	84	14.1% (r=2)	327 (r=2)	2
SKCM	*BRAF-M + PTEN-MD*	3	84	10.7% (r=3)	227 (r=3)	2
SKCM	*BRAF-M + CDKN2A-MD*	1	83	26.2% (r=1)	524 (r=1)	2
SKCM	*HULC-A + NRAS-M*	5	71	6.55% (r=28)	167 (r=9)	2
SKCM	*B2M-MD + FMN1/SNORD77/snoU13-D*	10	66	8.97% (r=6)	144 (r=36)	2
SKCM	*BRAF-M + TP53-M*	8	51	8.28% (r=9)	156 (r=7)	2
STAD	*ARID1A-MD + PIK3CA-M*	1	100	9.97% (r=38)	508 (r=34)	2
STAD	*d4(n=4) + TP53-M*	2	100	17.9% (r=3)	408 (r=3)	2
STAD	*ARID1A-MD + TP53-M*	6	96	13.8% (r=14)	319 (r=13)	2
STAD	*CCSER1-D + PIH1/WWOX-D + TP53-M*	9	91	10.5% (r=32)	300 (r=7)	3
STAD	*ARID1A-MD + KRAS-MA*	11	89	8.18% (r=78)	292 (r=70)	2
STAD	*SMAD4-MD + TP53-M*	5	83	11.5% (r=23)	340 (r=17)	2
STAD	*GMDS-D + TP53-M*	10	76	14.6% (r=10)	296 (r=8)	2
STAD	*KRAS-MA + TP53-M*	12	67	8.18% (r=82)	289 (r=39)	2

STAD	*ERBB2-A + FKSG52/PDE4D-D + PIH1/WWOX-D + TP53-M*	79	66	5.12% (r=369)	173 (r=60)	4
STAD	*PIH1/WWOX-D + PTPRD/RN7SL5P/SNORD27-D*	21	64	13.8% (r=13)	255 (r=25)	2
STAD	*d4(n=4) + IMMP2L/LRRN3-D +PTPRN2-D + PIH1/WWOX-D*	110	57	3.32% (r=1236)	156 (r=337)	4
STAD	*ARID1A-MD + SMAD4-MD*	22	55	7.93% (r=84)	255 (r=170)	2
STAD	*ARID1A-MD + FHIT/NPCDR1/U3-D + PIH1/WWOX-D*	14	52	7.42% (r=106)	281 (r=57)	3
STAD	*PIH1/WWOX-D + PIK3CA-M*	19	50	7.42% (r=104)	268 (r=56)	2
UCEC	*PIK3R1-M + PTEN-MD*	2	97	28.9% (r=2)	534 (r=2)	2
UCEC	*ARID1A-MD + CTNNB1-M + PTEN-MD*	38	81	9.92% (r=45)	189 (r=17)	3
UCEC	*SNORD37-D + TP53-M*	8	79	15.7% (r=10)	366 (r=25)	2
UCEC	*PIK3CA-M + PTEN-MD*	1	78	36.8% (r=1)	749 (r=1)	2
UCEC	*CTNNB1-M + PIK3CA-M*	6	75	16.5% (r=7)	421 (r=8)	2
UCEC	*ARID1A-MD + KRAS-M + PTEN-MD*	28	65	10.7% (r=37)	214 (r=14)	3
UCEC	*PIK3CA-M + TP53-M*	5	74	13.2% (r=18)	439 (r=16)	2

SJ Single rule objective function score. **NE** Number of events in rule.

Appendix Table S7: SELECT Common Event Duos Comparison

Table 7: Duos Identified by SELECT or CRSO Across 16 TCGA Cancer Types

Tissue	Mut1	Mut2	Algorithm	CovCRSO	CovSELECT	DuoConf
STAD	*ARID1A*	*RNF43*	**SELECT Only**	8.70	10.00	2
BLCA	*KDM6A*	*STAG2*	**SELECT Only**	7.90	6.00	11
BLCA	*FGFR3*	*STAG2*	**SELECT Only**	6.10	5.10	0
HNSC	*CASP8*	*FAT1*	**SELECT Only**	5.50	2.60	5
LUAD	*KEAP1*	*STK11*	**SELECT Only**	5.20	2.60	10
LUAD	*KRAS*	*RBM10*	**SELECT Only**	3.60	4.30	0
HNSC	*FAT1*	*RASA1*	**SELECT Only**	2.20	1.30	0
CESC	*HLA-A*	*HLA-B*	**SELECT Only**	2.10	2.10	0
BLCA	*C3orf70*	*CREBBP*	**SELECT Only**	2.00	1.30	0
BLCA	*CDKN1A*	*RB1*	**SELECT Only**	2.00	2.60	0
BLCA	*RBM10*	*STAG2*	**SELECT Only**	1.80	1.30	0
BRCA	*CDH1*	*ERBB2*	**SELECT Only**	1.60	1.00	0
LUAD	*RBM10*	*STK11*	**SELECT Only**	1.50	2.20	0
BRCA	*CDH1*	*FOXA1*	**SELECT Only**	0.93	0.92	0
BRCA	*CDH1*	*TBX3*	**SELECT Only**	0.83	0.82	0
BRCA	*CDH1*	*RUNX1*	**SELECT Only**	0.83	0.82	0
BRCA	*CBFB*	*GATA3*	**SELECT Only**	0.62	0.61	0
BRCA	*AKT1*	*GATA3*	**SELECT Only**	0.10	0.92	0
BRCA	*AKT1*	*MAP2K4*	**SELECT Only**	0.10	0.31	0
BLCA	*FGFR3*	*KDM6A*	**BOTH**	11.00	6.00	100
HNSC	*FAT1*	*NOTCH1*	**BOTH**	10.00	4.60	74
LUAD	*KRAS*	*STK11*	**BOTH**	10.00	6.50	99
BRCA	*CDH1*	*PIK3CA*	**BOTH**	5.60	6.20	99
KIRC	*PBRM1*	*SETD2*	**BOTH**	4.40	6.40	65
LGG	*IDH1*	*TP53*	**CRSO Only**	46.00	40.00	100
LGG	*ATRX*	*IDH1*	**CRSO Only**	40.00	18.00	100
LGG	*ATRX*	*TP53*	**CRSO Only**	39.00	15.00	71
UCEC	*PIK3CA*	*PTEN*	**CRSO Only**	37.00	30.00	100
UCEC	*PIK3R1*	*PTEN*	**CRSO Only**	29.00	24.00	100
UCEC	*ARID1A*	*PTEN*	**CRSO Only**	28.00	24.00	93
HNSC	*FAT1*	*TP53*	**CRSO Only**	27.00	14.00	100
UCEC	*ARID1A*	*PIK3CA*	**CRSO Only**	24.00	17.00	58
UCEC	*CTNNB1*	*PTEN*	**CRSO Only**	23.00	19.00	93
LUSC	*NFE2L2*	*TP53*	**CRSO Only**	22.00	12.00	99
LUSC	*PTEN*	*TP53*	**CRSO Only**	22.00	4.50	68
KIRC	*PBRM1*	*VHL*	**CRSO Only**	20.00	18.00	100
ESCA	*SMAD4*	*TP53*	**CRSO Only**	19.00	4.90	98
LGG	*CIC*	*IDH1*	**CRSO Only**	19.00	11.00	100
BLCA	*KDM6A*	*TP53*	**CRSO Only**	17.00	12.00	85
UCEC	*CTNNB1*	*PIK3CA*	**CRSO Only**	17.00	14.00	99
UCEC	*KRAS*	*PTEN*	**CRSO Only**	17.00	14.00	87
LUSC	*NF1*	*TP53*	**CRSO Only**	16.00	2.80	99
BLCA	*ARID1A*	*TP53*	**CRSO Only**	15.00	8.10	87
HNSC	*NOTCH1*	*TP53*	**CRSO Only**	15.00	5.60	65
LUAD	*KRAS*	*TP53*	**CRSO Only**	15.00	9.10	100
UCEC	*KRAS*	*PIK3CA*	**CRSO Only**	15.00	13.00	82
BLCA	*RB1*	*TP53*	**CRSO Only**	14.00	12.00	100
ESCA	*TP53*	*ZNF750*	**CRSO Only**	14.00	2.70	86
STAD	*ARID1A*	*TP53*	**CRSO Only**	14.00	7.10	96
BLCA	*ERBB2*	*TP53*	**CRSO Only**	13.00	4.70	100
BRCA	*PIK3CA*	*TP53*	**CRSO Only**	13.00	7.10	73
PRAD	*PTEN*	*TMPRSS2*	**CRSO Only**	13.00	0.00	86
UCEC	*PIK3CA*	*TP53*	**CRSO Only**	13.00	10.00	100
UCEC	*ARID1A*	*KRAS*	**CRSO Only**	13.00	11.00	87
UCEC	*ARID1A*	*CTNNB1*	**CRSO Only**	13.00	10.00	86
BLCA	*PIK3CA*	*TP53*	**CRSO Only**	12.00	8.10	89
GBM	*PTEN*	*TP53*	**CRSO Only**	12.00	3.40	94
LUAD	*EGFR*	*TP53*	**CRSO Only**	12.00	5.20	74
LUSC	*RB1*	*TP53*	**CRSO Only**	12.00	4.50	99

STAD	*SMAD4*	*TP53*	**CRSO Only**	12.00	2.40	83
GBM	*EGFR*	*PTEN*	**CRSO Only**	11.00	1.90	52
HNSC	*PIK3CA*	*TP53*	**CRSO Only**	11.00	8.30	75
HNSC	*NFE2L2*	*TP53*	**CRSO Only**	11.00	2.60	53
LUSC	*PIK3CA*	*TP53*	**CRSO Only**	11.00	9.60	63
SKCM	*BRAF*	*PTEN*	**CRSO Only**	11.00	3.80	100
STAD	*ARID1A*	*PIK3CA*	**CRSO Only**	10.00	9.30	100
LGG	*FUBP1*	*IDH1*	**CRSO Only**	8.80	4.60	59
LUAD	*NF1*	*TP53*	**CRSO Only**	8.80	5.70	77
LIHC	*RB1*	*TP53*	**CRSO Only**	8.50	2.10	50
BRCA	*ERBB2*	*TP53*	**CRSO Only**	8.40	0.31	83
SKCM	*BRAF*	*TP53*	**CRSO Only**	8.30	5.00	52
BLCA	*ARID1A*	*PIK3CA*	**CRSO Only**	8.20	4.70	97
ESCA	*NFE2L2*	*TP53*	**CRSO Only**	8.20	7.10	84
LUAD	*KEAP1*	*TP53*	**CRSO Only**	8.20	0.87	61
STAD	*ARID1A*	*KRAS*	**CRSO Only**	8.20	2.60	89
STAD	*KRAS*	*TP53*	**CRSO Only**	8.20	2.40	68
STAD	*ARID1A*	*SMAD4*	**CRSO Only**	7.90	1.10	55
KIRC	*ARID1A*	*VHL*	**CRSO Only**	7.20	0.95	100
BRCA	*PTEN*	*TP53*	**CRSO Only**	7.00	1.20	99
SKCM	*NRAS*	*TP53*	**CRSO Only**	6.60	4.70	100
LIHC	*ARID1A*	*CTNNB1*	**CRSO Only**	6.30	1.00	91
LUAD	*ATM*	*KRAS*	**CRSO Only**	6.10	3.50	73
LGG	*IDH1*	*PIK3CA*	**CRSO Only**	6.00	5.30	84
GBM	*RB1*	*TP53*	**CRSO Only**	5.90	3.70	66
BLCA	*ELF3*	*TP53*	**CRSO Only**	5.60	1.30	53
KIRC	*BAP1*	*VHL*	**CRSO Only**	5.50	4.00	98
BLCA	*ELF3*	*KDM6A*	**CRSO Only**	5.40	1.70	53
PRAD	*PTEN*	*TP53*	**CRSO Only**	5.30	0.00	69
CESC	*PIK3CA*	*PTEN*	**CRSO Only**	5.20	3.70	100
LUAD	*BRAF*	*TP53*	**CRSO Only**	5.20	4.30	66
GBM	*IDH1*	*TP53*	**CRSO Only**	4.80	3.40	100
CESC	*EP300*	*PIK3CA*	**CRSO Only**	4.70	2.60	74
LIHC	*PTEN*	*TP53*	**CRSO Only**	4.60	1.60	50
LIHC	*CTNNB1*	*RB1*	**CRSO Only**	4.40	0.00	53
GBM	*ATRX*	*TP53*	**CRSO Only**	4.00	0.00	63
HNSC	*CASP8*	*HRAS*	**CRSO Only**	3.80	0.99	73
BRCA	*MAP3K1*	*PIK3CA*	**CRSO Only**	3.70	3.70	100
CESC	*HLA-B*	*PIK3CA*	**CRSO Only**	3.70	1.00	72
GBM	*ATRX*	*IDH1*	**CRSO Only**	3.70	0.00	63

Duos identified by SELECT (i.e., predicted synergies) and high confidence GCDs (conf. $\geq$ 50) identified by CRSO among the common mutations for each cancer type.
