# Supplementary material for: Identifying modules of cooperating cancer drivers
Source: Mol Syst Biol. 2021 Mar 26;17(3):e9810. doi: 10.15252/msb.20209810 (PMC7995435; doi:10.15252/msb.20209810)
Supplement: Supplementary file 5 — Dataset EV4 [file MSB-17-e9810-s008.zip › DATASET EV4/CRSO_Report_COAD.pdf]

CRSO Output Report: COAD

Michael Klein

Nov 11 2020

Contents

1 Dataset Overview and Parameters **2**
1.1 Parameter table 2
1.2 Timing breakdown (in minutes) 2

2 Heatmaps of D and P **3**

3 Summary of K Best Rule Sets **4**
3.1 Performance and coverage of best rule sets 4
3.2 Table of rules that appear in any best rule set 5

4 Core Rule Set **6**
4.1 Table of core rule set rules 6
4.2 Event breakdown of core rule set 7
4.3 Core Rule Set Penalties: Before and After Assignment 8

5 Generalized Core Analysis **9**

6 Combined Core Table **13**

7 Dictionary of Copy Number Events **14**

1 Dataset Overview and Parameters

Number of samples = 362.
Number of events = 97.
Rule coverage requirement = 14 samples.
Rule assignment requirement = 11 samples.
Rule library size = 1792 rules.

1.1 Parameter table

Parameter Name	Value	Description	Category
n.cores	20	Number HPC Cores	Resources
msa	11	Minimum Samples Assigned	RS Constraint
rule.thresh	0.0387	Rule Coverage Thresh	Library Definition
max.rl	2000	Max Rule Library Size	Library Definition
p1.ntpr	40	P1 Num Trial Per Rule	Phase 1
p1.stop	24	P1 Stop Elimination	Phase 1
cut.size	0.25	P1 Cut Size	Phase 1
k.max.2	10	P2 Max K	Phase 2
max.nrs.p2	1000000	P2 Max RS Evaluated Per K	Phase 2
Total Time	10000000	P2 Max RS Family Check Per K	Phase 2
max.stored.p2	10	P2 Num Top RS Stored Per K	Phase 2
max.nrs.p3	200000	P3 Max RS Evaluated Per K	Phase 3
max.stored.p3	100	P3 Num Top RS Stored Per K	Phase 3
k.max.4	40	P4 Max K	Phase 4
max.nrs.p4	100000	P4 Max RS Evaluated	Phase 4
max.stored.p4	100	P4 Num Top RS Stored Per K	Phase 4
gc.iter	100	Num CG Iterations	Generalized Core
gc.eval	100	Num RS Per GC	Generalized Core
r.seed	111	Total Computational Time	Timing

1.2 Timing breakdown (in minutes)

P1	PS	P2	P3	P4	GC	Total
16.3	32.2	73.6	24.7	36.9	41.4	225

2 Heatmaps of D and P

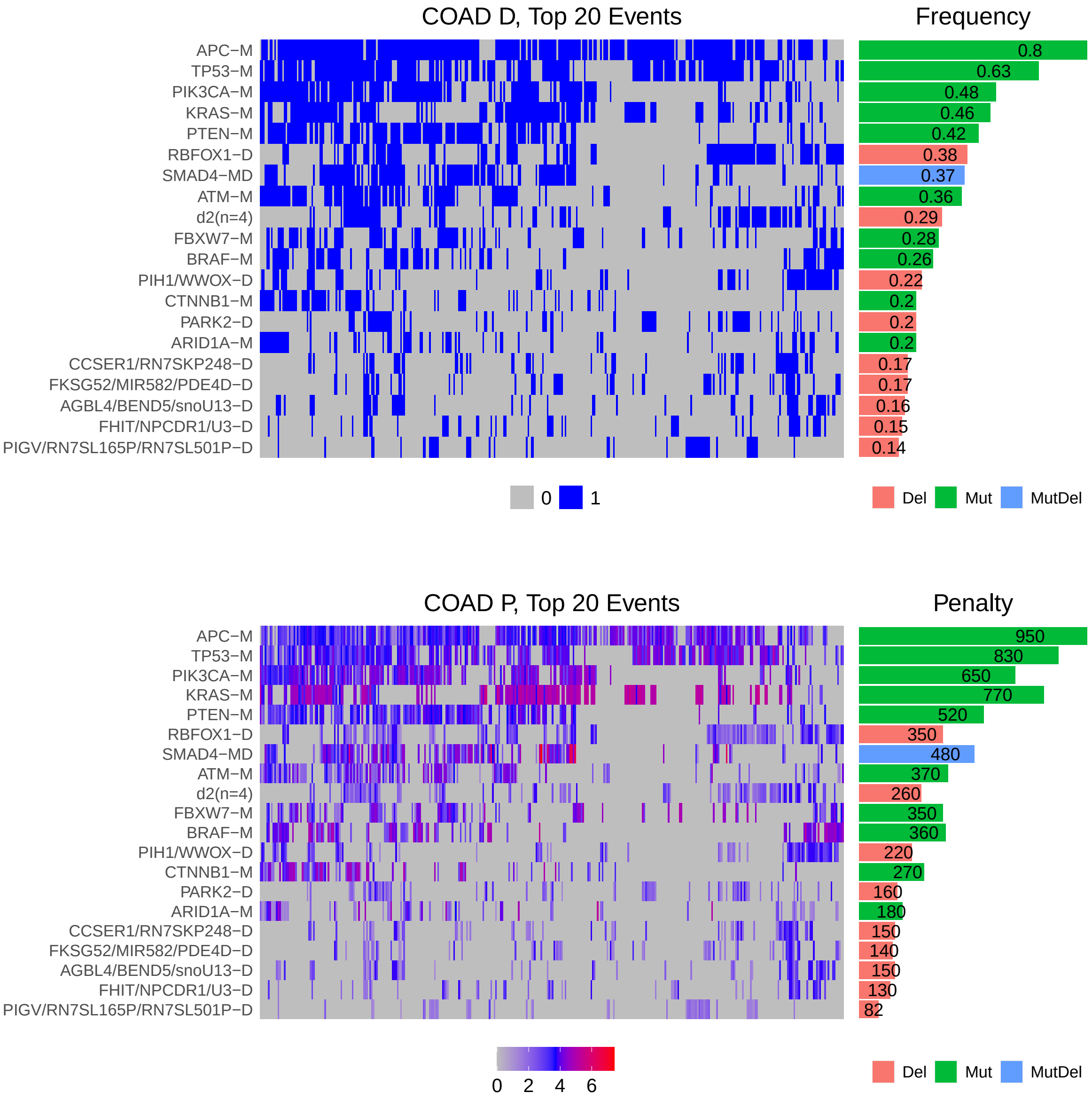

Figure 1: Heatmap of D and P. Top: binary representation of top 20 most frequent events. Top right shows event types and frequencies across the population. Bottom: Penalty matrix for top 20 most frequent events. Bottom right shows total event penalties. Events are ordered by frequency, from top to bottom. Samples are ordered using hierarchical clustering. Wild-type events indicated in grey. Event suffix -M: mutation, -A: amplification, -D: deletion, -MD: mutDel, -MA: mutAmp.

3 Summary of K Best Rule Sets

3.1 Performance and coverage of best rule sets

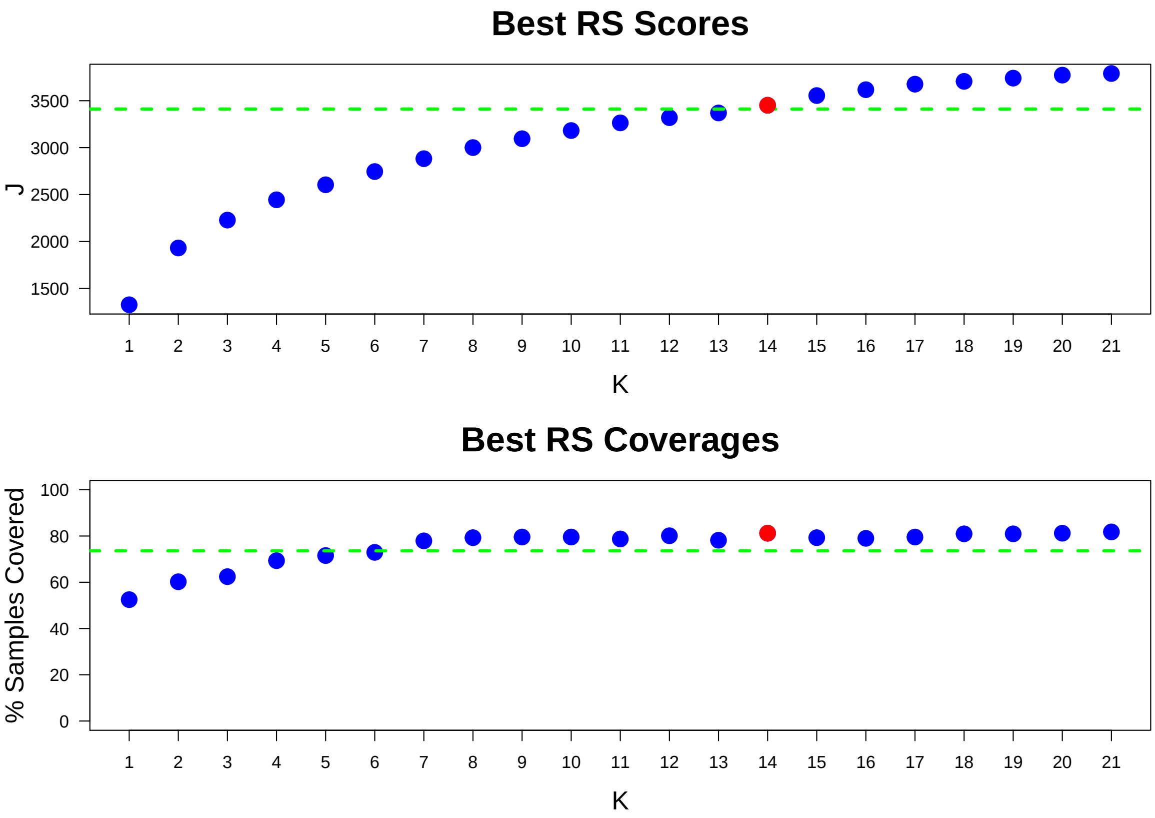

Figure 2: Performance and Coverage of Best Filtered RS for K. The core was determined to be the smallest RS that achieved at least 90% of maximum coverage and at least 90% of maximum performance.

3.2 Table of rules that appear in any best rule set

IR	Rule	PC	Ks
16	APC-M+ATM-M+PIK3CA-M+PTEN-M	17	3-14,17-21
34	APC-M+PTEN-M+SMAD4-MD	20	5-21
1	APC-M+TP53-M	52	1-14
147	ATM-M+CTNNB1-M+KRAS-M+PIK3CA-M+PTEN-M+TP53-M	4.7	10-21
6	BRAF-M+PIH1/WWOX-D+RBFOX1-D	5.5	11-21
7	APC-M+KRAS-M+PIK3CA-M	24	2-3,13-21
146	APC-M+BRAF-M+FBXW7-M+PIK3CA-M+PTEN-M	5.5	9-10,14-21
2	APC-M+KRAS-M	40	4-12
37	APC-M+d2(n=4)	23	13-21
8	RBFOX1-D+TP53-M	25	14-21
87	ATM-M+PIK3CA-M+SMAD4-MD+TP53-M	11	8-14
3	APC-M+KRAS-M+TP53-M	26	15-21
9	APC-M+PIK3CA-M+PTEN-M+TP53-M	20	15-21
25	APC-M+PARK2-D+TP53-M	14	15-21
125	CHD6/SNORA26-A+COX4I2/ID1/MIR3193-A+TP53-M	7.5	15-21
5	APC-M+ATM-M+PIK3CA-M+SMAD4-MD+TP53-M	10	15-20
26	APC-M+KRAS-M+SMAD4-MD	15	13-14,16-19
76	KRAS-M+RBFOX1-D+TP53-M	11	8-13
102	KRAS-M+PIK3CA-M+PTEN-M+TP53-M	12	4-9
138	BRAF-M+SMAD4-MD+TP53-M	9.9	16-21
319	KRAS-M+PIK3CA-M+PTEN-M+SMAD4-MD+TP53-M	6.1	16-21
156	KRAS-M+PIK3CA-M+SMAD4-MD+TP53-M	8.8	10-14
196	BRAF-M+PIK3CA-M+PTEN-M+SMAD4-MD+TP53-M	5.2	11-15
36	KRAS-M+RBFOX1-D	16	18-21
38	BRAF-M+RBFOX1-D	12	7-10
194	APC-M+ATM-M+KRAS-M+PTEN-M+RBFOX1-D	4.7	14-17
280	APC-M+ATM-M+FBXW7-M+PTEN-M+TP53-M	6.9	18-21
49	APC-M+BRAF-M+PIK3CA-M+PTEN-M	10	11-13
61	APC-M+ATM-M+KRAS-M	15	20-21
86	PIK3CA-M+SMAD4-MD+TP53-M	18	6-7
105	APC-M+NRAS-M+TP53-M	6.1	20-21
383	APC-M+CTNNB1-M+KRAS-M+PTEN-M	8.3	19-20
28	APC-M+SMAD4-MD+TP53-M	24	21
55	APC-M+ATM-M+CTNNB1-M+PIK3CA-M+TP53-M	8.6	21
66	KRAS-M+SMAD4-MD	17	15
70	d2(n=4)+RBFOX1-D+TP53-M	13	12

ID = Rule IDs, rules are numbered according to importance rank determined from phase 1
PC = Percent of samples covered **Ks** = Membership in best RS

4 Core Rule Set

Core K = 14.
Core rule set coverage = 81.2%.

4.1 Table of core rule set rules

IR	Rule	CR	SJR	SJ	NSC	NSA	PC	PA	FracA
1	APC-M + TP53-M	1	1	1330	190	66	52	18.0	0.35
6	BRAF-M + PIH1/WWOX-D + RBFOX1-D	762.5	814	210	20	14	5.5	3.9	0.70
7	APC-M + KRAS-M + PIK3CA-M	20	7	1010	88	33	24	9.1	0.38
8	RBFOX1-D + TP53-M	18	66.5	557	92	20	25	5.5	0.22
16	APC-M + ATM-M + PIK3CA-M + PTEN-M	49.5	22	776	61	20	17	5.5	0.33
26	APC-M + KRAS-M + SMAD4-MD	75	42	650	56	18	15	5.0	0.32
34	APC-M + PTEN-M + SMAD4-MD	33	28	746	74	23	20	6.4	0.31
37	APC-M + d2(n=4)	22	108.5	482	85	13	23	3.6	0.15
87	ATM-M + PIK3CA-M + SMAD4-MD + TP53-M	153	68	553	41	14	11	3.9	0.34
146	APC-M + BRAF-M + FBXW7-M + PIK3CA-M + PTEN-M	762.5	387.5	309	20	14	5.5	3.9	0.70
147	ATM-M + CTNNB1-M + KRAS-M + PIK3CA-M + PTEN-M + TP53-M	1061.5	294	343	17	17	4.7	4.7	1.00
156	KRAS-M + PIK3CA-M + SMAD4-MD + TP53-M	272	110.5	481	32	18	8.8	5.0	0.56
194	APC-M + ATM-M + KRAS-M + PTEN-M + RBFOX1-D	1061.5	464.5	286	17	11	4.7	3.0	0.65
196	BRAF-M + PIK3CA-M + PTEN-M + SMAD4-MD + TP53-M	841.5	343.5	324	19	13	5.2	3.6	0.68

ID = Rule IDs, rules are numbered according to importance rank determined from phase 1
CR = Coverage rank
SJ/SJR = Single rule performance / performance rank
NSC/PC = Number/percent of samples covered
NSA/PA = Number/percent of samples assigned
FracA = Fraction of covered samples assigned to rule

4.2 Event breakdown of core rule set

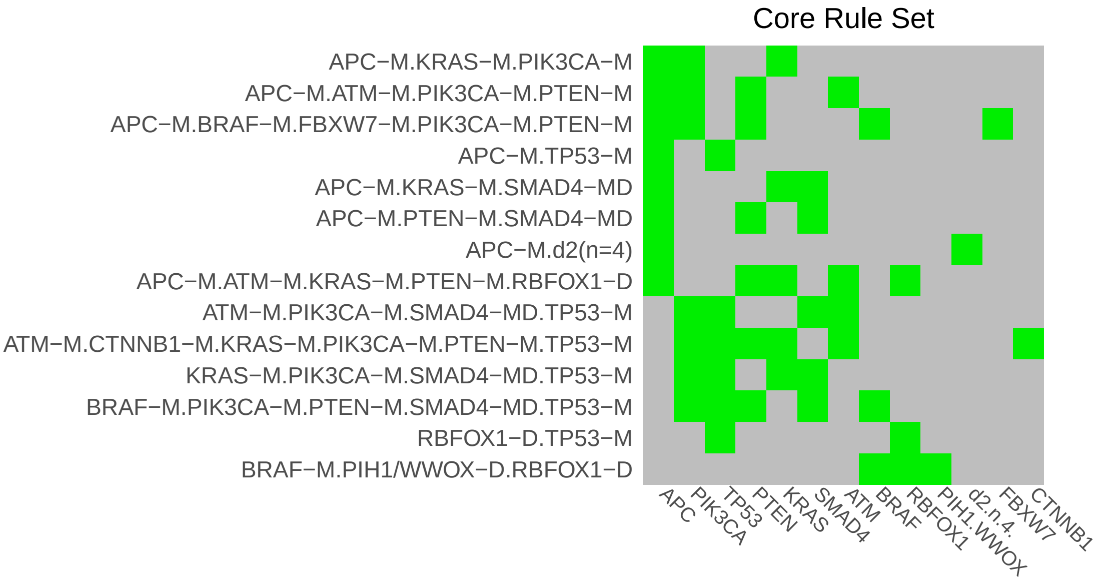

Figure 3: Visualization of core rule set. Rows are rules, columns are events. Events are ordered from left in decreasing rule membership frequency.

4.3 Core Rule Set Penalties: Before and After Assignment

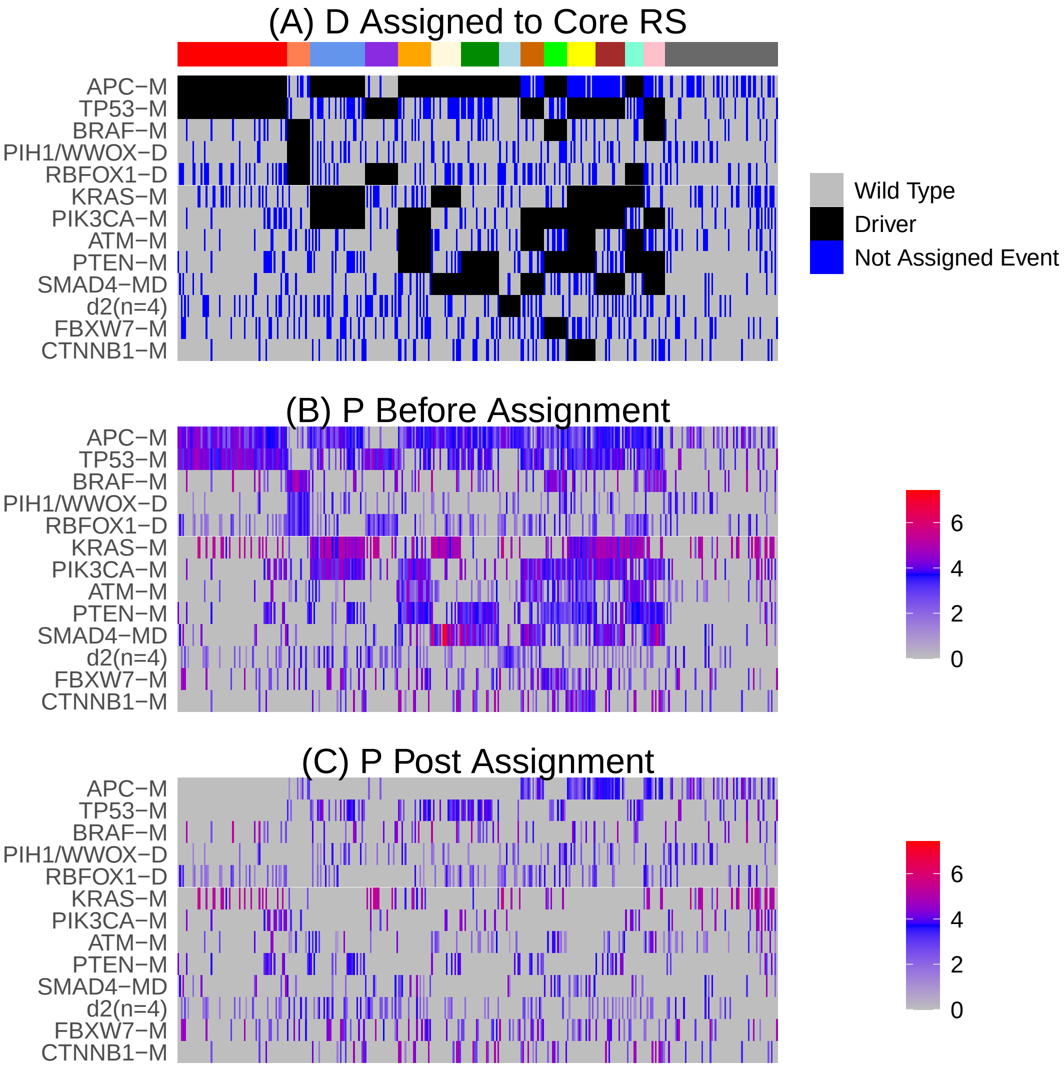

Figure 4: Heatmaps of D and P under core rule set assignment. Events are ordered by frequency. Samples are ordered according rule set membership, as indicated by the color bar. The dark grey group of samples are not assigned to any rule. A) For each sample, assigned events are designated as drivers and are shown in black, unassigned events are shown in blue. B-C) Heatmap of P before and after assignment to core rule set.

5 Generalized Core Analysis

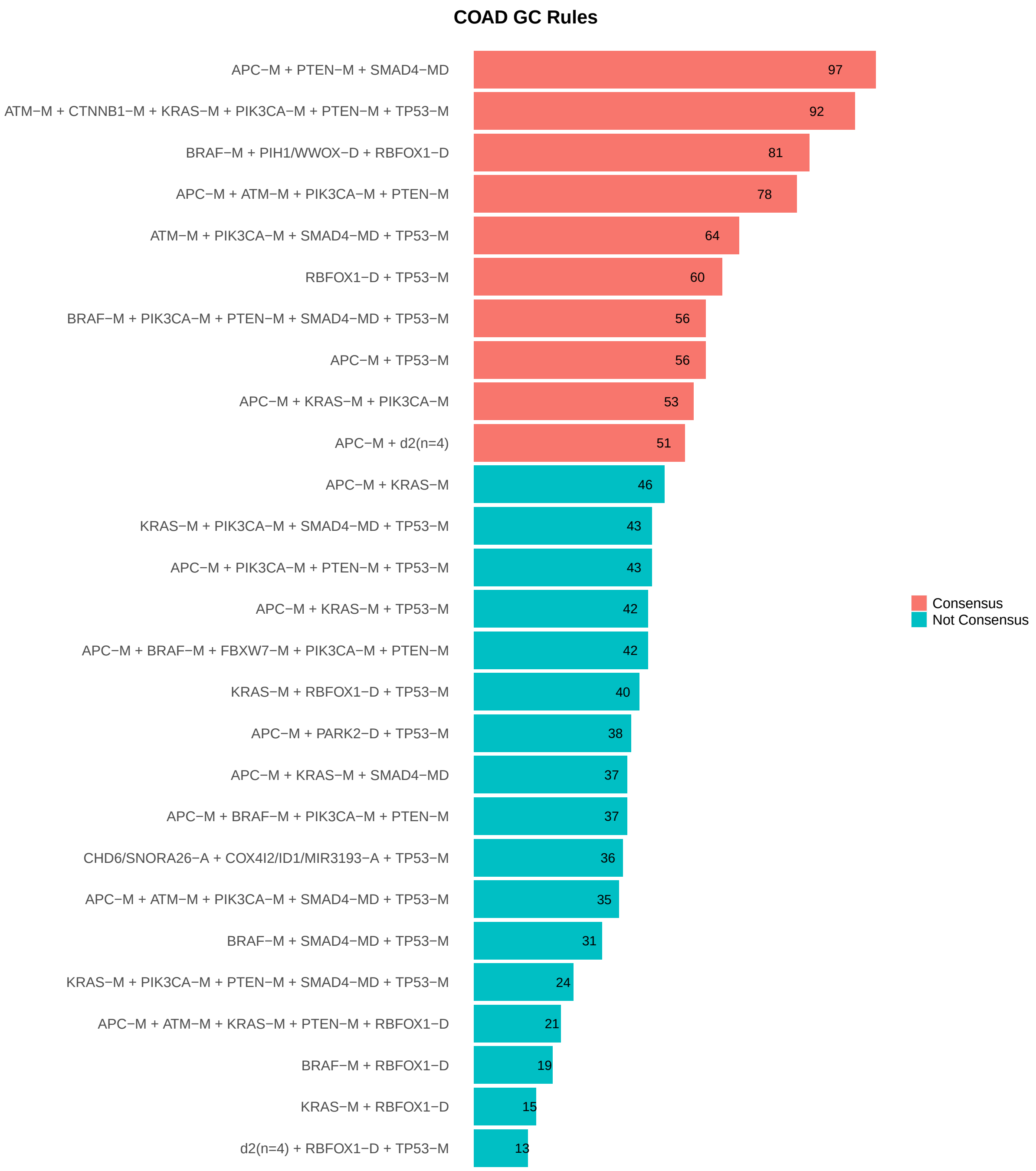

Figure 5: GCRs

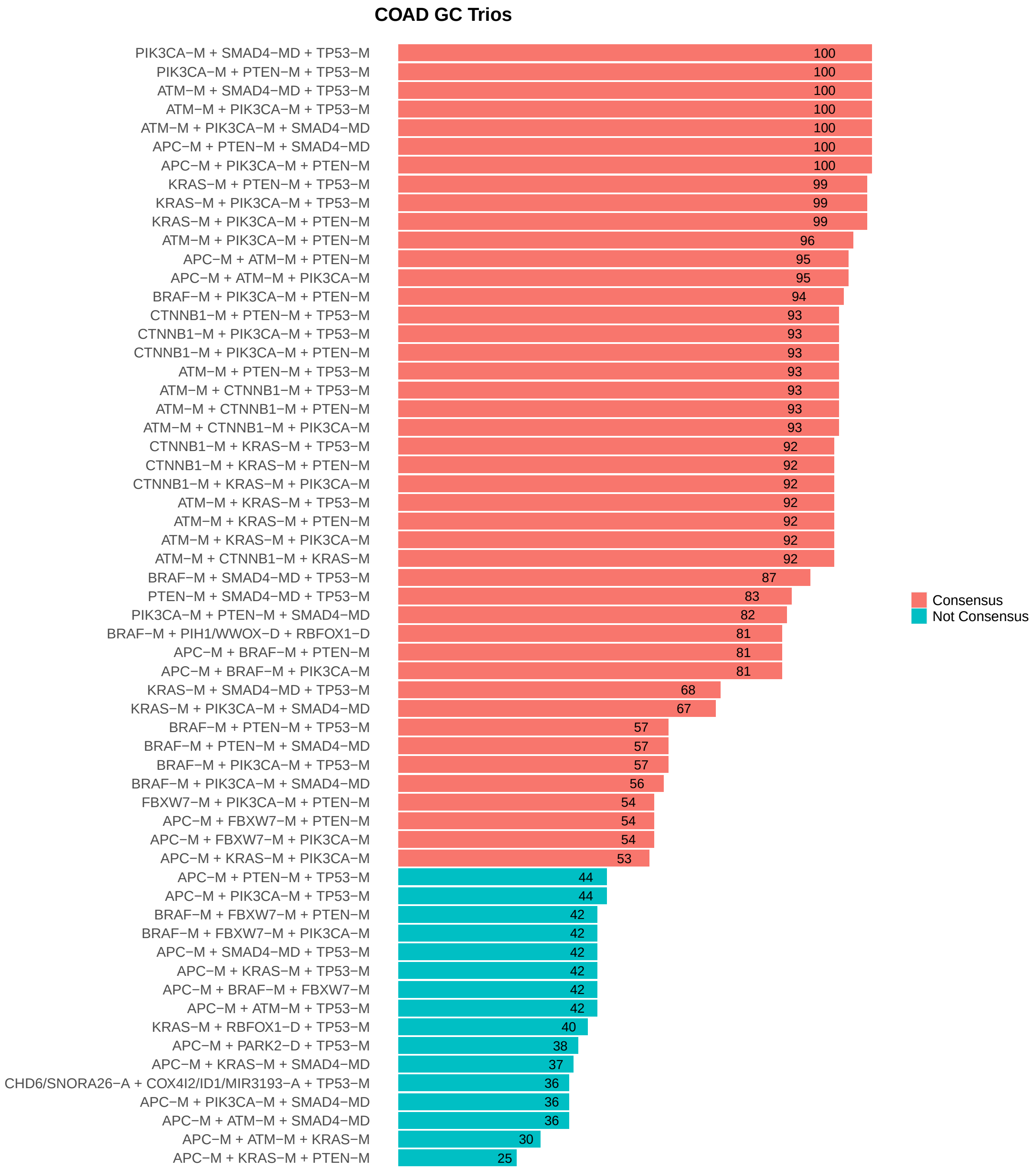

Figure 6: GCTs

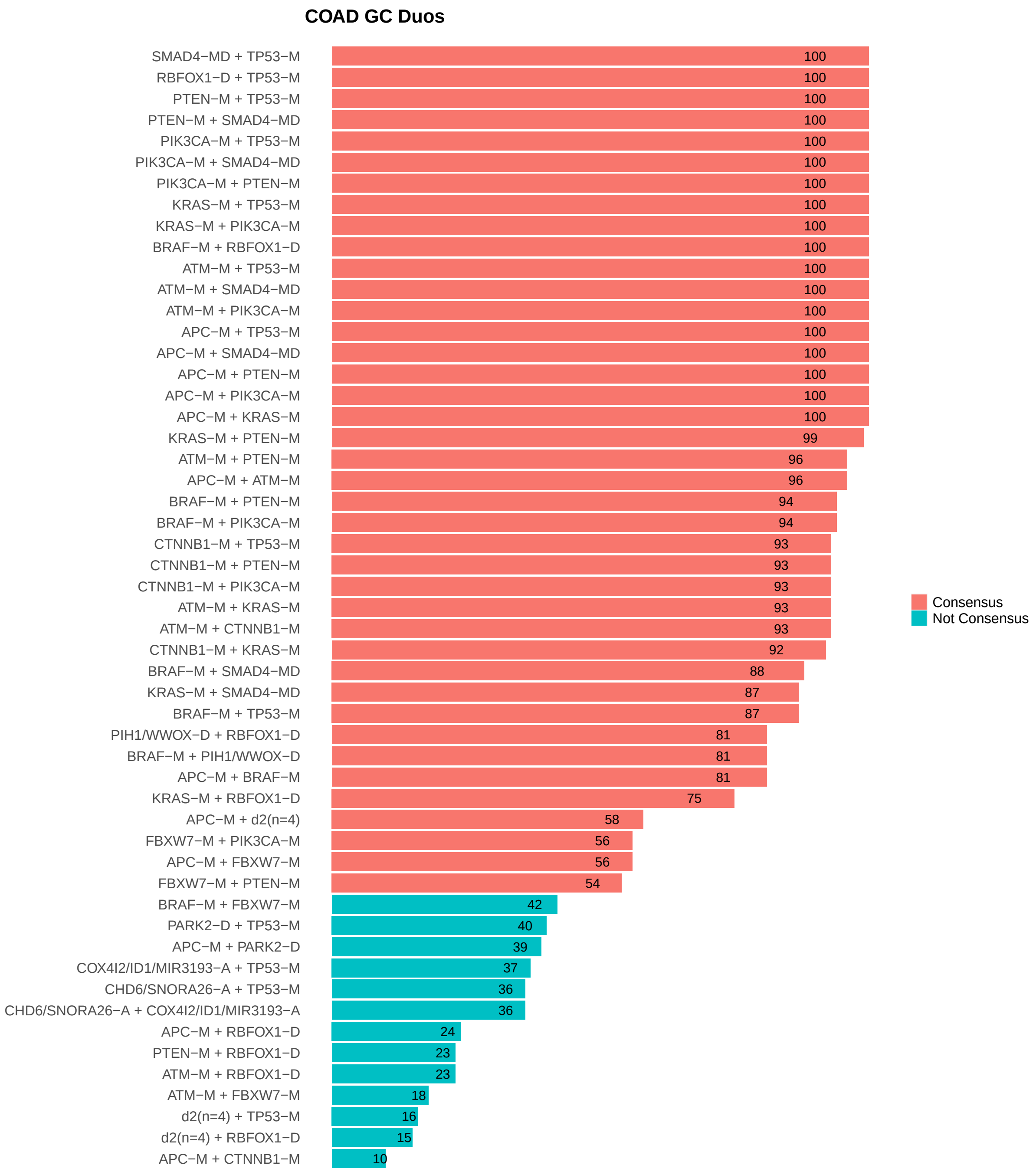

Figure 7: GCDs

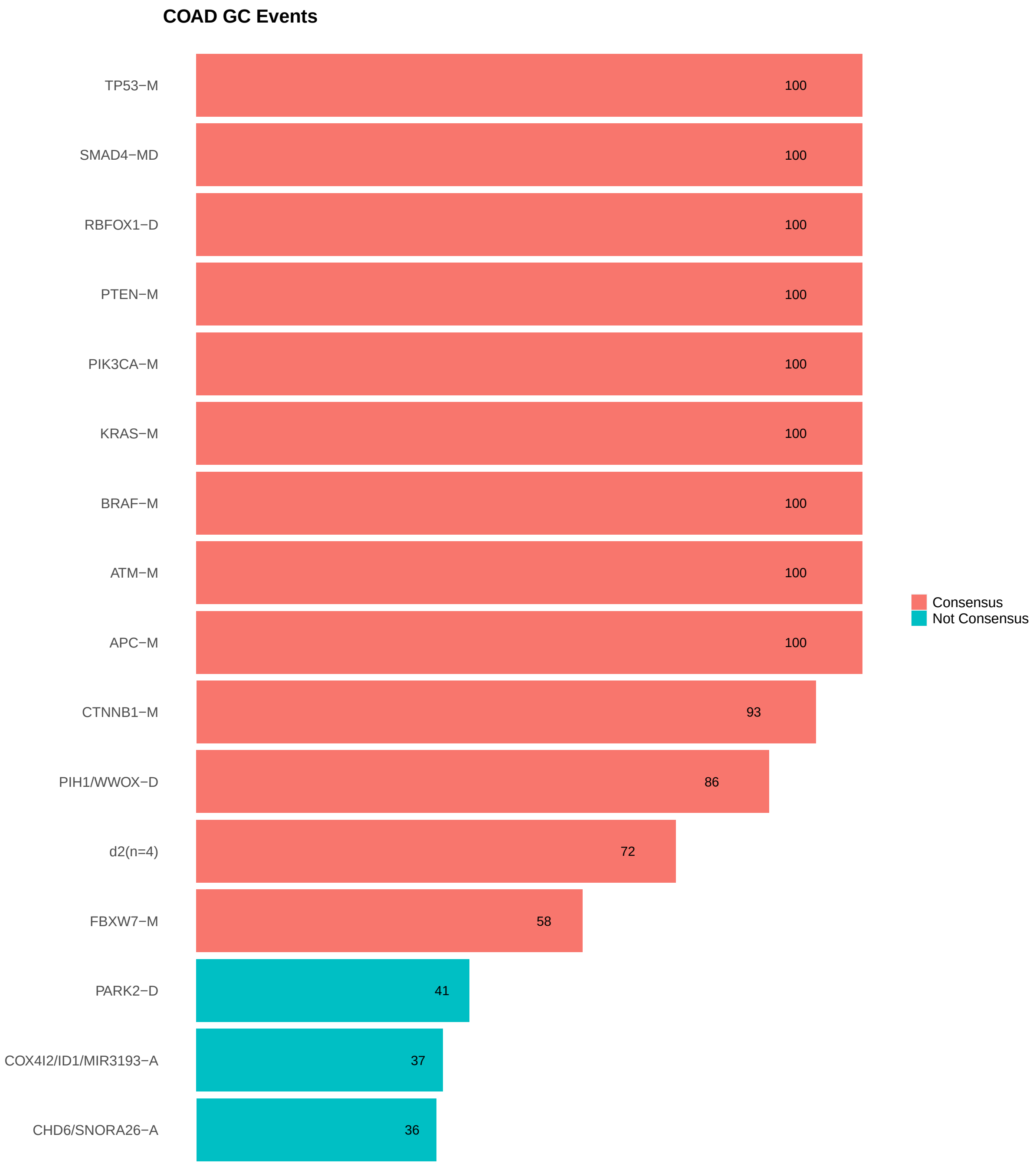

Figure 8: GCEs

6 Combined Core Table

Rule	Core_Type	P1_Rank	Confidence	Coverage	SJ	Fraction_Assigned
APC-M + PTEN-M + SMAD4-MD	Both	34	97	20.4% (r=33)	711 (r=28)	0.180
ATM-M + CTNNB1-M + KRAS-M + PIK3CA-M + PTEN-M + TP53-M	Both	147	92	4.7% (r=1074)	443 (r=293)	0.120
BRAF-M + PIH1/WWOX-D + RBFOX1-D	Both	6	81	5.52% (r=735)	1030 (r=817)	0.850
APC-M + ATM-M + PIK3CA-M + PTEN-M	Both	16	78	16.9% (r=51)	825 (r=22)	0.082
ATM-M + PIK3CA-M + SMAD4-MD + TP53-M	Both	87	64	11.3% (r=151)	512 (r=68)	0.240
RBFOX1-D + TP53-M	Both	8	60	25.4% (r=18)	999 (r=67)	0.330
APC-M + TP53-M	Both	1	56	52.5% (r=1)	1330 (r=1)	0.610
BRAF-M + PIK3CA-M + PTEN-M + SMAD4-MD + TP53-M	Both	196	56	5.25% (r=847)	403 (r=342)	0.160
APC-M + KRAS-M + PIK3CA-M	Both	7	53	24.3% (r=20)	1010 (r=7)	0.520
APC-M + d2(n=4)	Both	37	51	23.5% (r=22)	697 (r=108)	0.210
KRAS-M + PIK3CA-M + SMAD4-MD + TP53-M	Core	156	43	8.84% (r=272)	431 (r=110)	0.340
APC-M + BRAF-M + FBXW7-M + PIK3CA-M + PTEN-M	Core	146	42	5.52% (r=792)	443 (r=388)	0.050
APC-M + KRAS-M + SMAD4-MD	Core	26	37	15.5% (r=75)	756 (r=42)	0.390
APC-M + ATM-M + KRAS-M + PTEN-M + RBFOX1-D	Core	194	21	4.7% (r=1104)	406 (r=464)	0.059

7 Dictionary of Copy Number Events

CNV	Genes	Event_Name
a1	ID1, COX4I2, MIR3193	COX4I2/ID1/MIR3193-A
a2	SNORA26|ENSG00000212224.1, CHD6	CHD6/SNORA26-A
d1	RBFOX1	RBFOX1-D
d2	RNA5SP475, RN7SL864P, FLRT3, MACROD2	d2(n=4)
d3	PIH1, WWOX	PIH1/WWOX-D
d4	PARK2	PARK2-D
d5	RN7SKP248, CCSER1	CCSER1/RN7SKP248-D
d6	FKSG52, MIR582, PDE4D	FKSG52/MIR582/PDE4D-D
d7	snoU13|ENSG00000239144.1, BEND5, AGBL4	AGBL4/BEND5/snoU13-D
d8	U3|ENSG00000212211.1, NPCDR1, FHIT	FHIT/NPCDR1/U3-D
d9	RN7SL165P, RN7SL501P, PIGV	PIGV/RN7SL165P/RN7SL501P-D

Dictionary of important CNVs. Copy number events that are within any of the best K rule sets, or within the generalized core rules, or among the 20 most frequent events.
