# Supplementary material for: Identifying modules of cooperating cancer drivers
Source: Mol Syst Biol. 2021 Mar 26;17(3):e9810. doi: 10.15252/msb.20209810 (PMC7995435; doi:10.15252/msb.20209810)
Supplement: Supplementary file 6 — Dataset EV5 [file MSB-17-e9810-s010.zip › DATASET EV5/CRSO_Report_ESCA.pdf]

CRSO Output Report: ESCA

Michael Klein

Nov 11 2020

Contents

1 Dataset Overview and Parameters **2**
- 1.1 Parameter table 2
- 1.2 Timing breakdown (in minutes) 2

2 Heatmaps of D and P **3**

3 Summary of K Best Rule Sets **4**
- 3.1 Performance and coverage of best rule sets 4
- 3.2 Table of rules that appear in any best rule set 5

4 Core Rule Set **6**
- 4.1 Table of core rule set rules 6
- 4.2 Event breakdown of core rule set 7
- 4.3 Core Rule Set Penalties: Before and After Assignment 8

5 Generalized Core Analysis **9**

6 Combined Core Table **13**

7 Dictionary of Copy Number Events **14**

1 Dataset Overview and Parameters

Number of samples = 184.
Number of events = 92.
Rule coverage requirement = 10 samples.
Rule assignment requirement = 6 samples.
Rule library size = 1894 rules.

1.1 Parameter table

Parameter Name	Value	Description	Category
n.cores	20	Number HPC Cores	Resources
msa	6	Minimum Samples Assigned	RS Constraint
rule.thresh	0.0543	Rule Coverage Thresh	Library Definition
max.rl	2000	Max Rule Library Size	Library Definition
p1.ntpr	40	P1 Num Trial Per Rule	Phase 1
p1.stop	24	P1 Stop Elimination	Phase 1
cut.size	0.25	P1 Cut Size	Phase 1
k.max.2	10	P2 Max K	Phase 2
max.nrs.p2	200000	P2 Max RS Evaluated Per K	Phase 2
Total Time	114	P2 Max RS Family Check Per K	Phase 2
max.stored.p2	10	P2 Num Top RS Stored Per K	Phase 2
max.nrs.p3	200000	P3 Max RS Evaluated Per K	Phase 3
max.stored.p3	100	P3 Num Top RS Stored Per K	Phase 3
k.max.4	40	P4 Max K	Phase 4
max.nrs.p4	100000	P4 Max RS Evaluated	Phase 4
max.stored.p4	100	P4 Num Top RS Stored Per K	Phase 4
gc.iter	100	Num CG Iterations	Generalized Core
gc.eval	100	Num RS Per GC	Generalized Core
r.seed	111	Total Computational Time	Timing

1.2 Timing breakdown (in minutes)

P1	PS	P2	P3	P4	GC	Total
9.78	6.78	7.61	18	33.6	38.4	114

2 Heatmaps of D and P

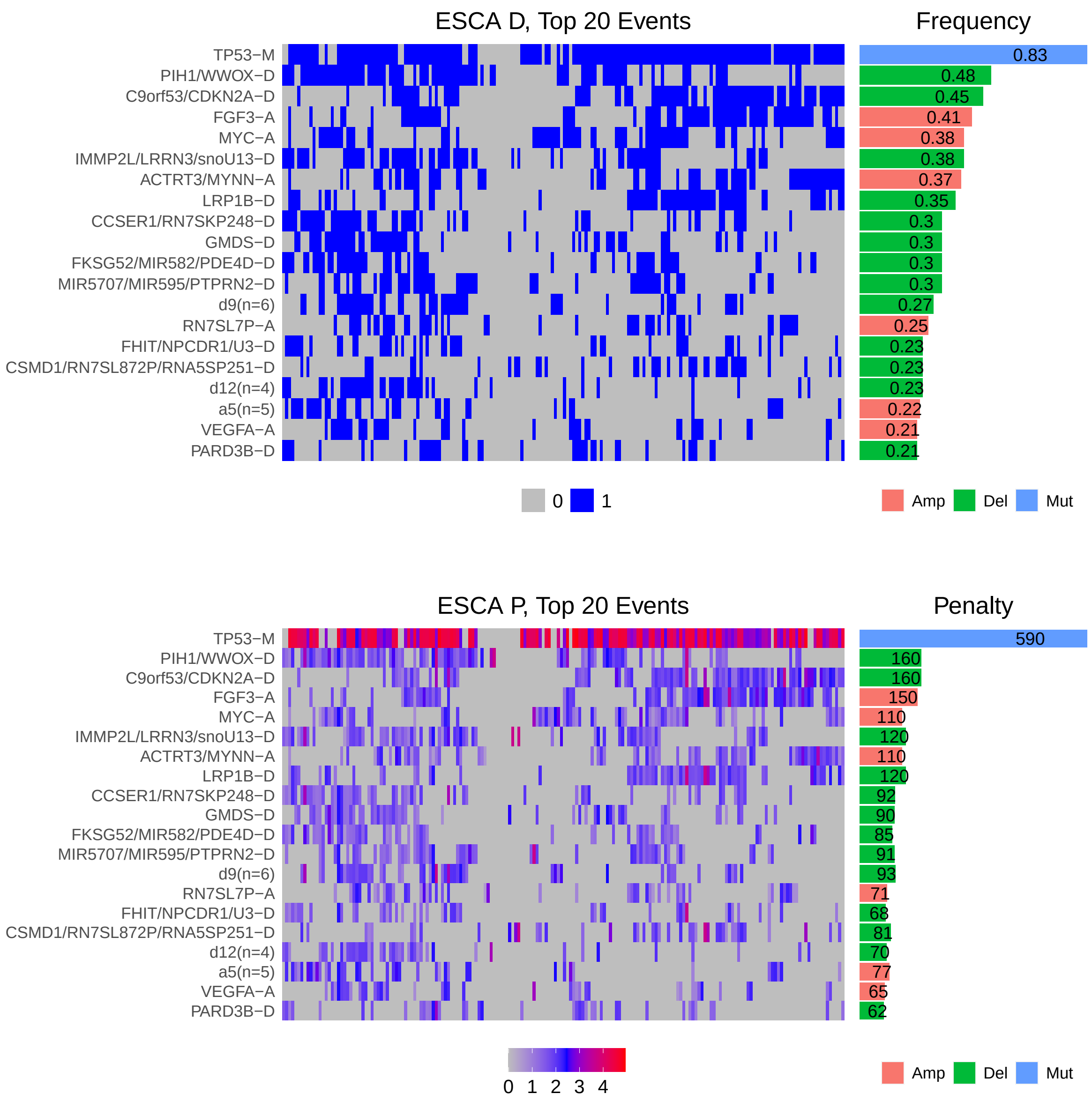

Figure 1: Heatmap of D and P. Top: binary representation of top 20 most frequent events. Top right shows event types and frequencies across the population. Bottom: Penalty matrix for top 20 most frequent events. Bottom right shows total event penalties. Events are ordered by frequency, from top to bottom. Samples are ordered using hierarchical clustering. Wild-type events indicated in grey. Event suffix -M: mutation, -A: amplification, -D: deletion, -MD: mutDel, -MA: mutAmp.

3 Summary of K Best Rule Sets

3.1 Performance and coverage of best rule sets

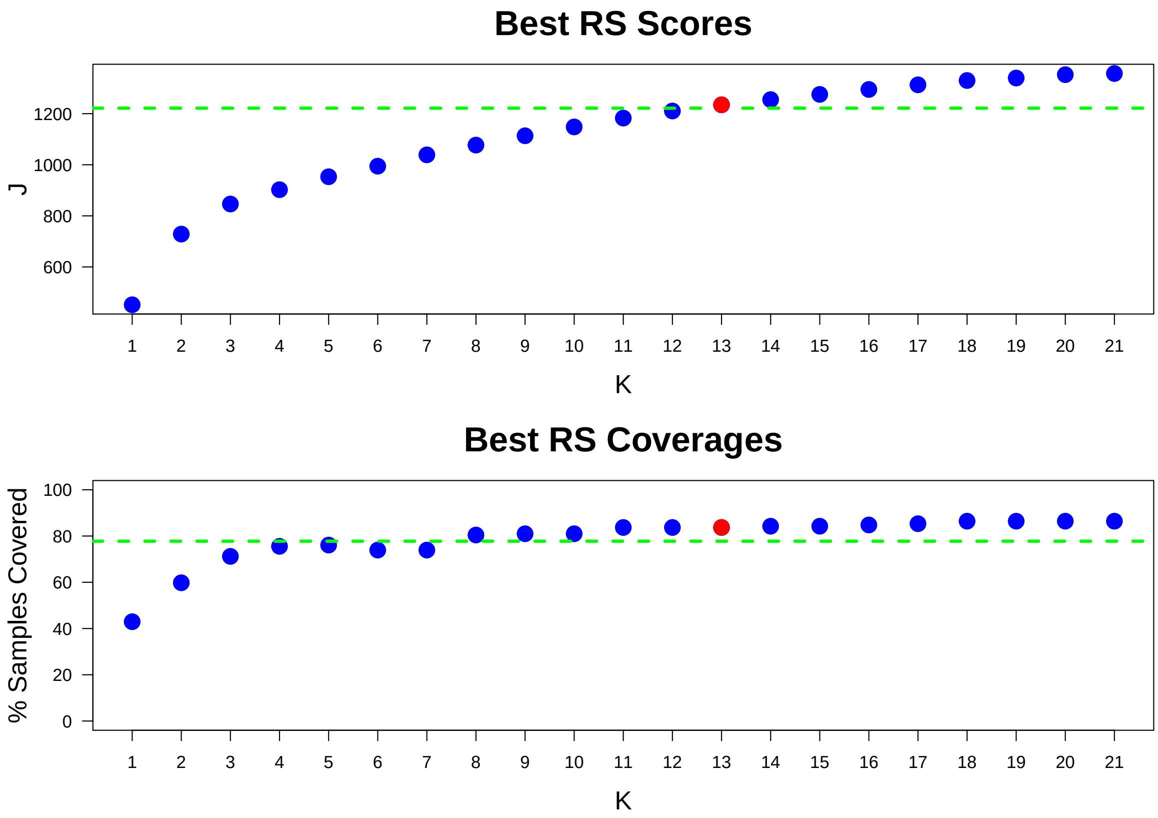

Figure 2: Performance and Coverage of Best Filtered RS for K. The core was determined to be the smallest RS that achieved at least 90% of maximum coverage and at least 90% of maximum performance.

3.2 Table of rules that appear in any best rule set

IR	Rule	PC	Ks
2	C9orf53/CDKN2A-D+FGF3-A+TP53-M	27	2-21
6	MYC-A+TP53-M	33	4-21
13	GMDS-D+PIH1/WWOX-D+TP53-M	18	6-21
15	SMAD4-MD+TP53-M	19	6-21
17	ACTRT3/MYNN-A+FGF3-A+IMMP2L/LRRN3/snoU13-D+MIR5707/MIR595/PTPRN2-D+TP53-M	7.1	7-21
140	MIR5707/MIR595/PTPRN2-D+PIH1/WWOX-D	16	8-21
7	LRP1B-D+TP53-M	33	3-14
21	CCSER1/RN7SKP248-D+d9(n=6)+IMMP2L/LRRN3/snoU13-D+PIH1/WWOX-D+TP53-M	6.5	11-21
116	a5(n=5)+CCSER1/RN7SKP248-D+FKSG52/MIR582/PDE4D-D+PIH1/WWOX-D+TP53-M	5.4	11-21
71	ACTRT3/MYNN-A+NFE2L2-M+TP53-M	6	9-18
112	d9(n=6)+FHIT/NPCDR1/U3-D+IMMP2L/LRRN3/snoU13-D+PIH1/WWOX-D+TP53-M	6	13-21
23	TP53-M+ZNF750-MD	14	11-19
88	snoU13-D+TP53-M	11	15-21
90	IMMP2L/LRRN3/snoU13-D+LRP1B-D+TP53-M	12	15-21
92	FGF3-A+PIK3CA-M+TP53-M	5.4	12-14,19-21
104	ACTRT3/MYNN-A+CSMD1/RN7SL872P/RNA5SP251-D+FGF3-A+LRP1B-D+TP53-M	8.7	15-20
182	C9orf53/CDKN2A-D+MIR5707/MIR595/PTPRN2-D+RN7SL7P-A+TP53-M	7.1	17-21
20	CCSER1/RN7SKP248-D+FKSG52/MIR582/PDE4D-D+IMMP2L/LRRN3/snoU13-D+PIH1/WWOX-D+TP53-M	7.6	6-10
1	PIH1/WWOX-D+TP53-M	40	2-5
37	PIK3CA-M+TP53-M	8.7	15-18
53	PARD3B-D+PIH1/WWOX-D+TP53-M	10	16-19
122	IMMP2L/LRRN3/snoU13-D+PIH1/WWOX-D+RNU6ATAC20P-A	9.8	18-21
79	NFE2L2-M+TP53-M	8.2	19-21
29	PARD3B-D+TP53-M	17	20-21
57	C9orf53/CDKN2A-D+TP53-M+ZNF750-MD	9.2	20-21
128	C9orf53/CDKN2A-D+CAT/ELF5-A+CD44-A+TP53-M	6	
209	ACTRT3/MYNN-A+FGF3-A+TP53-M+ZNF750-MD	5.4	20-21
4	C9orf53/CDKN2A-D+TP53-M	43	1
64	ACTRT3/MYNN-A+C9orf53/CDKN2A-D+LRP1B-D+TP53-M	12	20
91	CSMD1/RN7SL872P/RNA5SP251-D+FGF3-A+LRP1B-D+TP53-M	10	21
196	FKSG52/MIR582/PDE4D-D+IMMP2L/LRRN3/snoU13-D+MIR5707/MIR595/PTPRN2-D+RN7SL7P-A+TP53-M	6	5
220	d9(n=6)+FGF3-A+LRP1B-D+TP53-M	6	21

ID = Rule IDs, rules are numbered according to importance rank determined from phase 1
PC = Percent of samples covered **Ks** = Membership in best RS

4 Core Rule Set

Core K = 13.
Core rule set coverage = 83.7%.

4.1 Table of core rule set rules

IR	Rule	CR	SJR	SJ	NSC	NSA	PC	PA	FracA
2	C9orf53/CDKN2A-D + FGF3-A + TP53-M	9.5	4	377	49	29	27	16.0	0.59
6	MYC-A + TP53-M	5	5	341	61	14	33	7.6	0.23
7	LRP1B-D + TP53-M	6	7	336	60	11	33	6.0	0.18
13	GMDS-D + PIH1/WWOX-D + TP53-M	40	19	248	34	15	18	8.2	0.44
15	SMAD4-MD + TP53-M	36.5	35	202	35	9	19	4.9	0.26
17	ACTRT3/MYNN-A + FGF3-A + IMMP2L/LRRN3/snoU13-D + MIR5707/MIR595/PTPRN2-D + TP53-M	741.5	158.5	135	13	13	7.1	7.1	1.00
21	CCSER1/RN7SKP248-D + d9(n=6) + IMMP2L/LRRN3/snoU13-D + PIH1/WWOX-D + TP53-M	940.5	145	138	12	9	6.5	4.9	0.75
23	TP53-M + ZNF750-MD	96	105	151	25	10	14	5.4	0.40
71	ACTRT3/MYNN-A + NFE2L2-M + TP53-M	1219.5	339.5	108	11	8	6	4.3	0.73
92	FGF3-A + PIK3CA-M + TP53-M	1640	424.5	102	10	9	5.4	4.9	0.90
112	d9(n=6) + FHIT/NPCDR1/U3-D + IMMP2L/LRRN3/snoU13-D + PIH1/WWOX-D + TP53-M	1219.5	259	116	11	6	6	3.3	0.55
116	a5(n=5) + CCSER1/RN7SKP248-D + FKSG52/MIR582/PDE4D-D + PIH1/WWOX-D + TP53-M	1640	339.5	108	10	10	5.4	5.4	1.00
140	MIR5707/MIR595/PTPRN2-D + PIH1/WWOX-D	53.5	486	97.3	30	11	16	6.0	0.37

ID = Rule IDs, rules are numbered according to importance rank determined from phase 1
CR = Coverage rank
SJ/SJR = Single rule performance / performance rank
NSC/PC = Number/percent of samples covered
NSA/PA = Number/percent of samples assigned
FracA = Fraction of covered samples assigned to rule

4.2 Event breakdown of core rule set

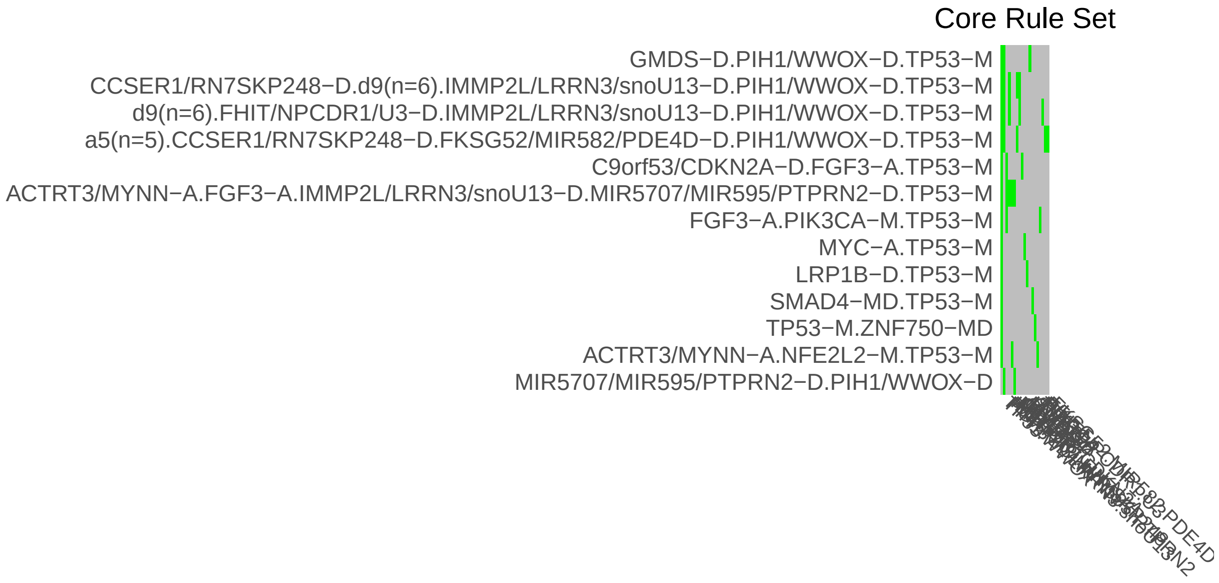

Figure 3: Visualization of core rule set. Rows are rules, columns are events. Events are ordered from left in decreasing rule membership frequency.

4.3 Core Rule Set Penalties: Before and After Assignment

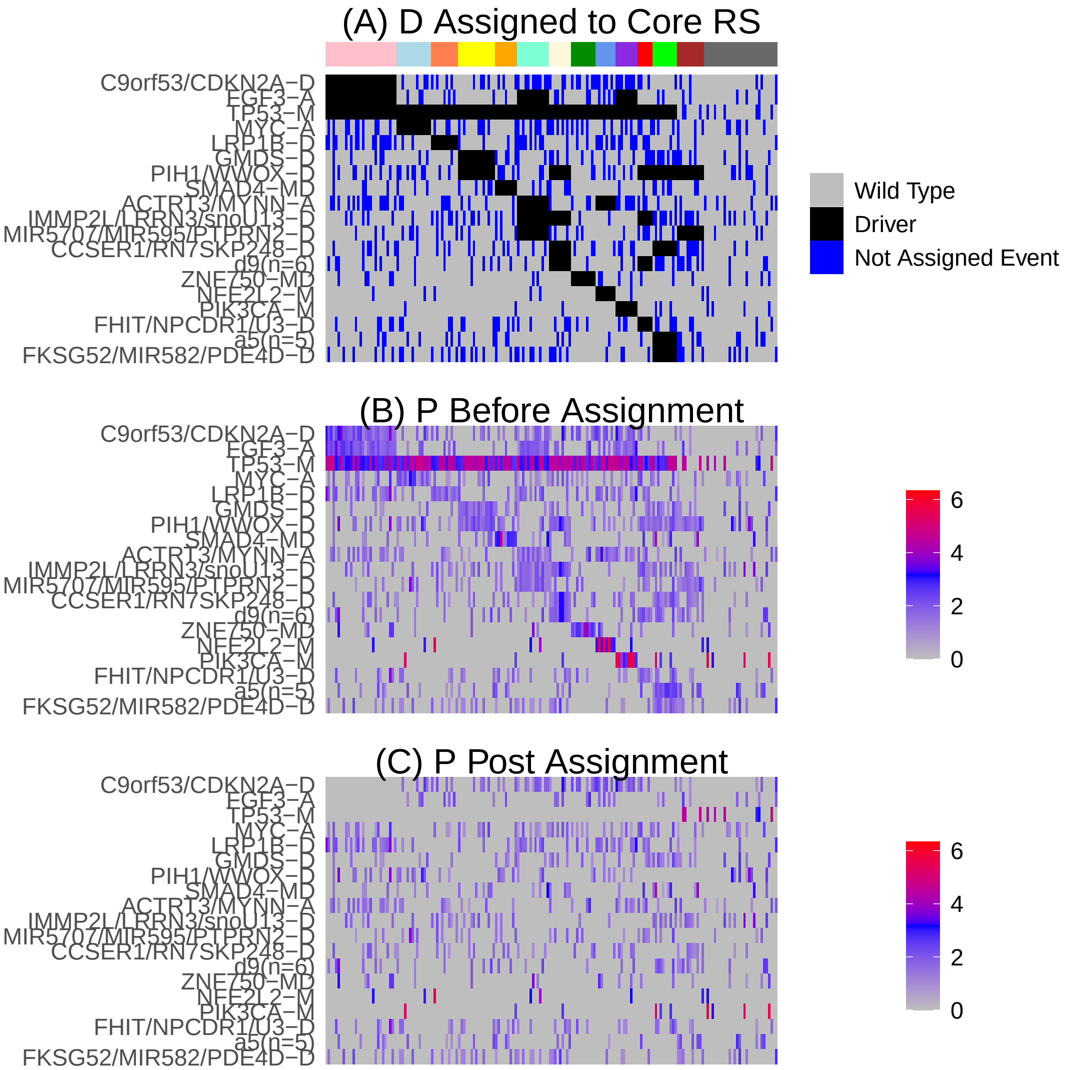

Figure 4: Heatmaps of D and P under core rule set assignment. Events are ordered by frequency. Samples are ordered according rule set membership, as indicated by the color bar. The dark grey group of samples are not assigned to any rule. A) For each sample, assigned events are designated as drivers and are shown in black, unassigned events are shown in blue. B-C) Heatmap of P before and after assignment to core rule set.

5 Generalized Core Analysis

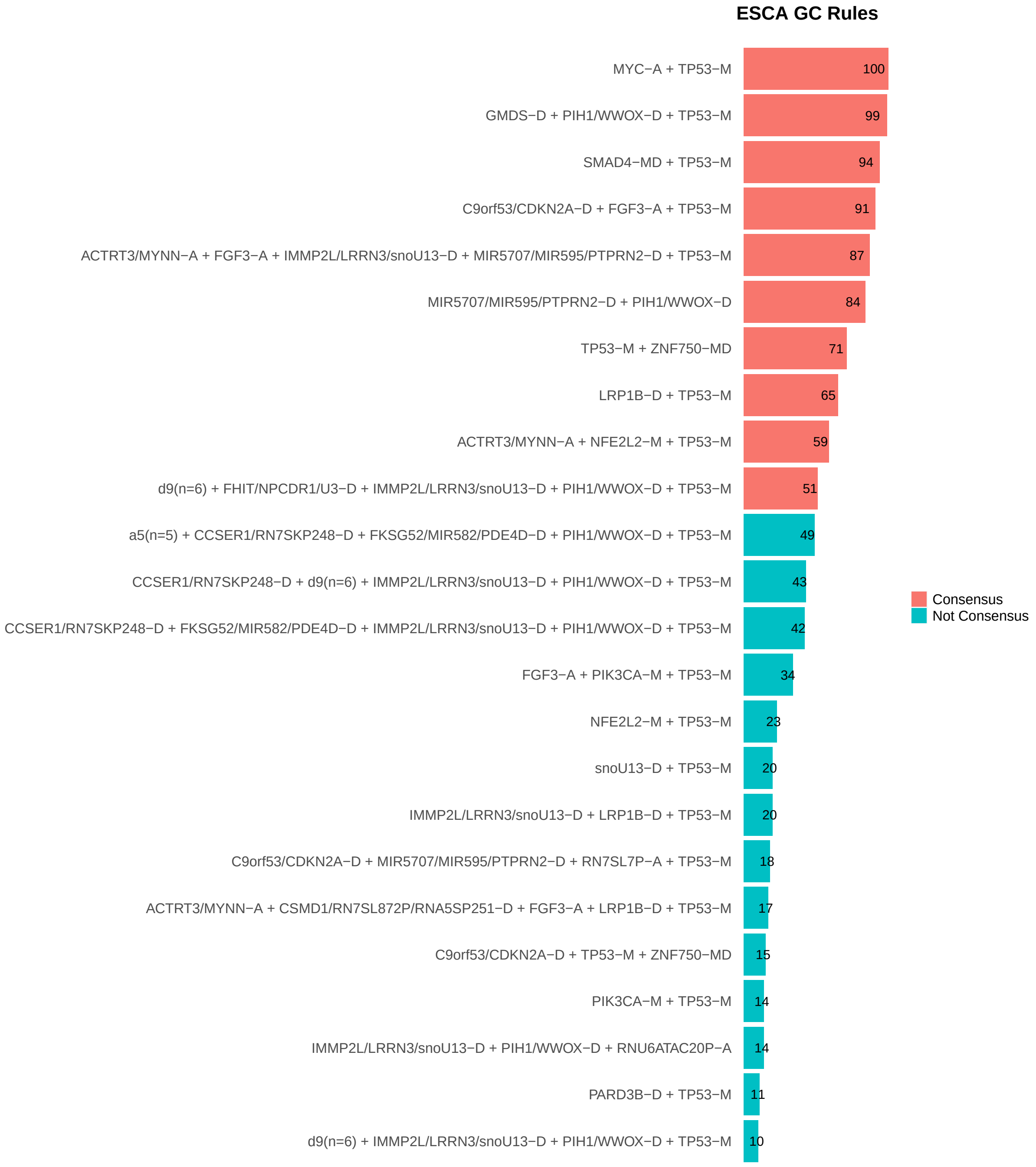

Figure 5: GCRs

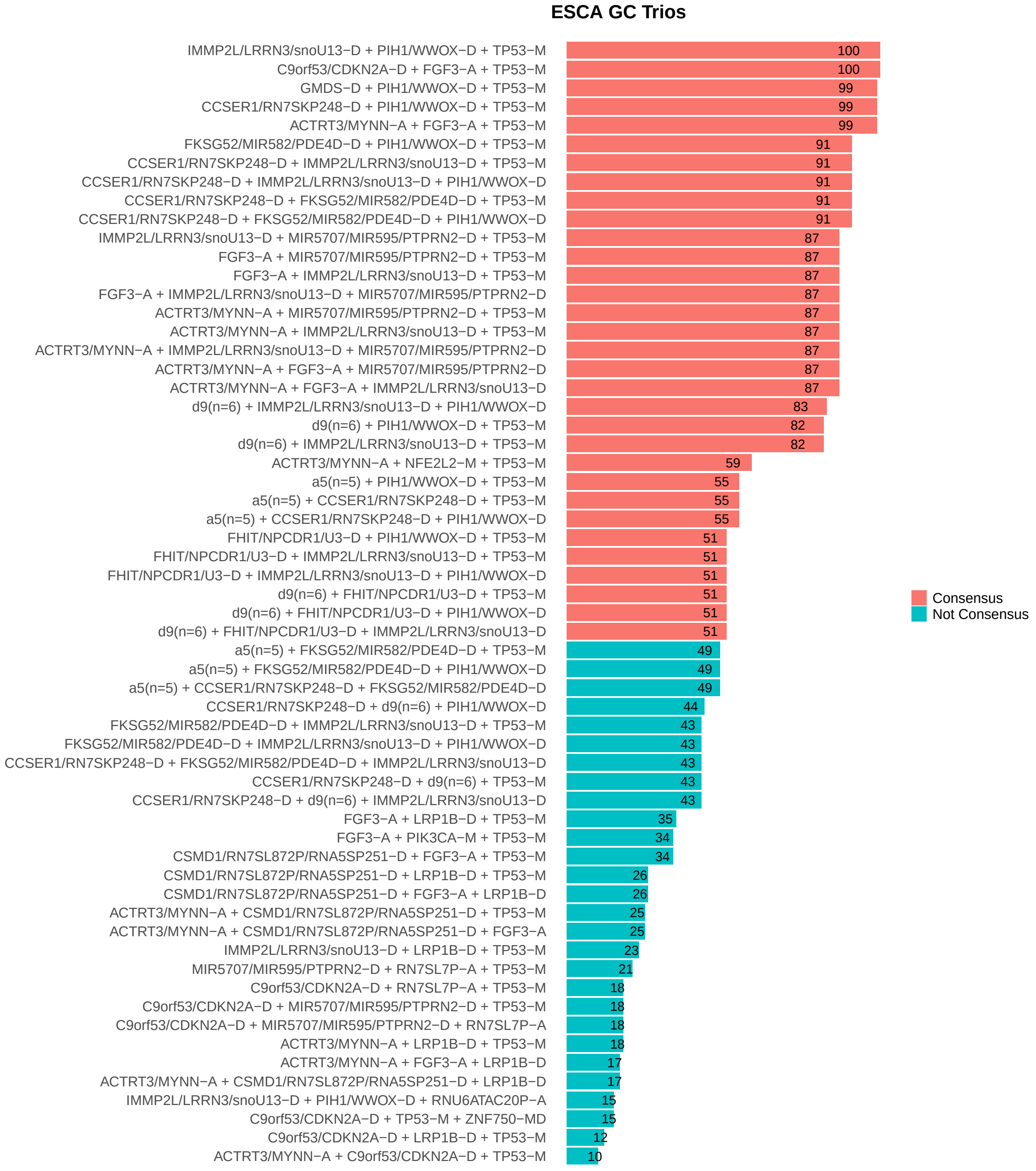

Figure 6: GCTs

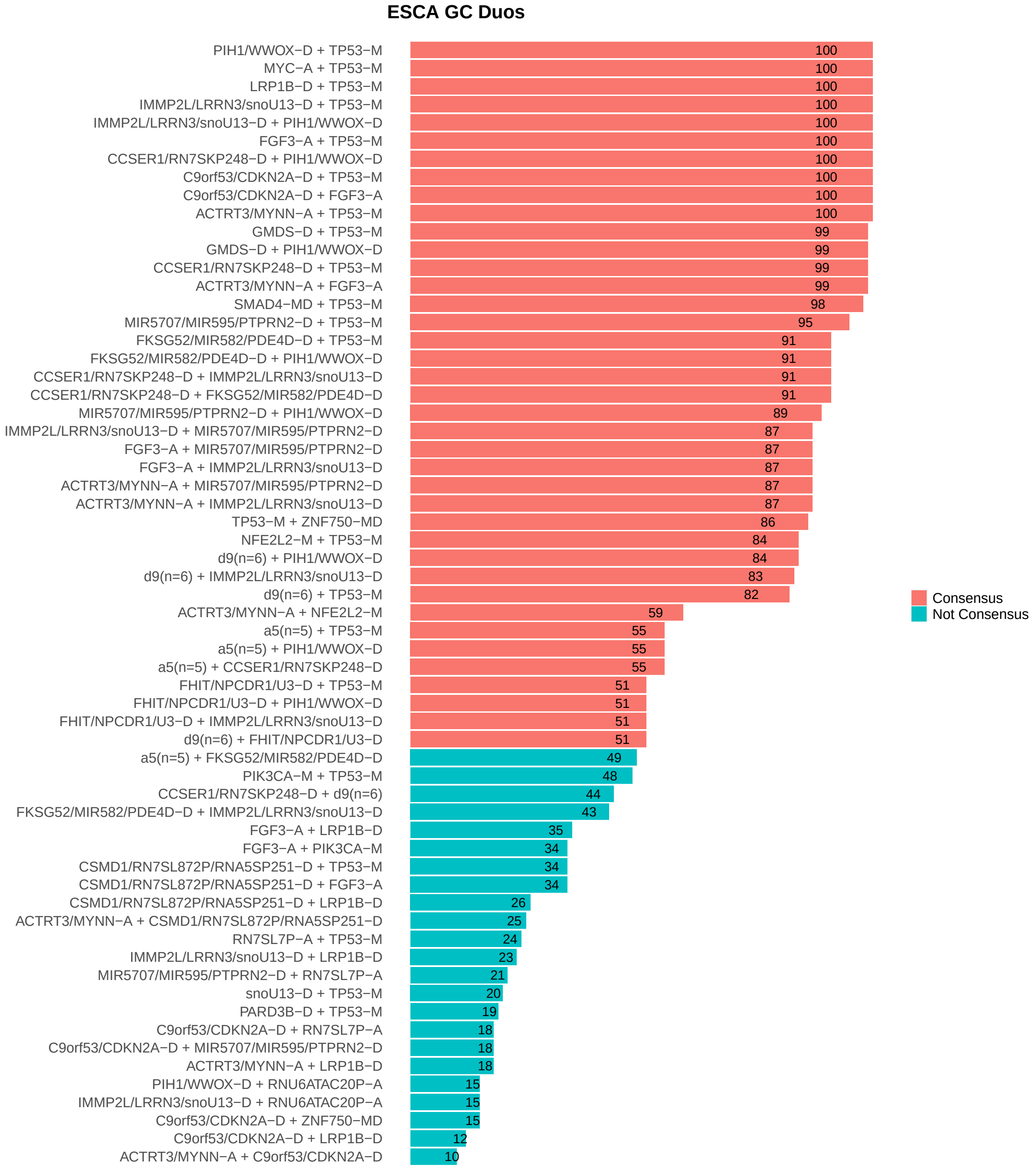

Figure 7: GCDs

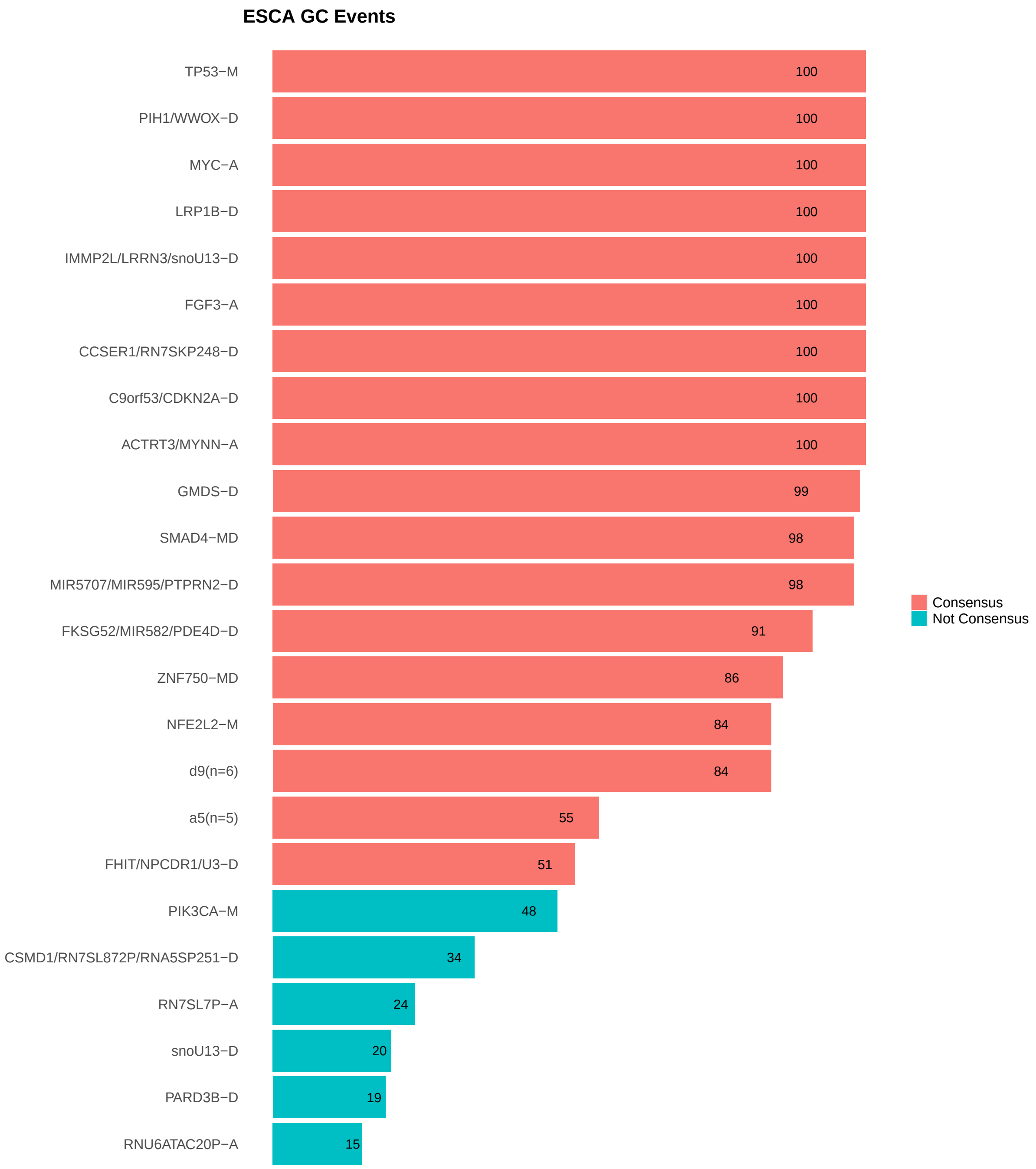

Figure 8: GCEs

6 Combined Core Table

Rule	Core_Type	P1_Rank	Confidence	Coverage	SJ	Fraction_Assigned
MYC-A + TP53-M	Both	6	100	33.2% (r=5)	340 (r=5)	0.520
GMDS-D + PIH1/WWOX-D + TP53-M	Both	13	99	18.5% (r=42)	276 (r=19)	0.290
SMAD4-MD + TP53-M	Both	15	94	19% (r=36)	272 (r=35)	0.600
C9orf53/CDKN2A-D + FGF3-A + TP53-M	Both	2	91	26.6% (r=10)	428 (r=4)	0.220
ACTRT3/MYNN-A + FGF3-A + IMMP2L/LRRN3/snoU13-D + MIR5707/MIR595/PTPRN2-D + TP53-M	Both	17	87	7.07% (r=797)	261 (r=157)	0.077
MIR5707/MIR595/PTPRN2-D + PIH1/WWOX-D	Both	140	84	16.3% (r=53)	139 (r=486)	0.470
TP53-M + ZNF750-MD	Both	23	71	13.6% (r=102)	242 (r=108)	0.640
LRP1B-D + TP53-M	Both	7	65	32.6% (r=6)	336 (r=7)	0.680
ACTRT3/MYNN-A + NFE2L2-M + TP53-M	Both	71	59	5.98% (r=1280)	169 (r=345)	0.180
d9(n=6) + FHIT/NPCDR1/U3-D + IMMP2L/LRRN3/snoU13-D + PIH1/WWOX-D + TP53-M	Both	112	51	5.98% (r=1378)	149 (r=260)	0.000
a5(n=5) + CCSER1/RN7SKP248-D + FKSG52/MIR582/PDE4D-D + PIH1/WWOX-D + TP53-M	Core	116	49	5.43% (r=1863)	148 (r=340)	0.100
CCSER1/RN7SKP248-D + d9(n=6) + IMMP2L/LRRN3/snoU13-D + PIH1/WWOX-D + TP53-M	Core	21	43	6.52% (r=1049)	246 (r=143)	0.083
FGF3-A + PIK3CA-M + TP53-M	Core	92	34	5.43% (r=1778)	155 (r=428)	0.400

7 Dictionary of Copy Number Events

CNV	Genes	Event__Name
a1	FGF3	FGF3-A
a2	MYC	MYC-A
a3	MYNN, ACTRT3	ACTRT3/MYNN-A
a4	RN7SL7P	RN7SL7P-A
a5	IKZF3, MIR4728, ERBB2, GRB7, MIEN1	a5(n=5)
a6	VEGFA	VEGFA-A
a8	RNU6ATAC20P	RNU6ATAC20P-A
a15	CAT, ELF5	CAT/ELF5-A
a20	CD44	CD44-A
d1	PIH1, WWOX	PIH1/WWOX-D
d2	CDKN2A, C9orf53	C9orf53/CDKN2A-D
d3	snoU13|ENSG00000238922.1, LRRN3, IMMP2L	IMMP2L/LRRN3/snoU13-D
d4	LRP1B	LRP1B-D
d5	RN7SKP248, CCSER1	CCSER1/RN7SKP248-D
d6	GMDS	GMDS-D
d7	FKSG52, MIR582, PDE4D	FKSG52/MIR582/PDE4D-D
d8	MIR595, PTPRN2, MIR5707	MIR5707/MIR595/PTPRN2-D
d9	snoU13|ENSG00000238969.1, MIR548F5, MIR3915, RNA5SP501, DMD, FTHL17	d9(n=6)
d10	U3|ENSG00000212211.1, NPCDR1, FHIT	FHIT/NPCDR1/U3-D
d11	RN7SL872P, RNA5SP251, CSMD1	CSMD1/RN7SL872P/RNA5SP251-D
d12	RNA5SP475, RN7SL864P, FLRT3, MACROD2	d12(n=4)
d13	PARD3B	PARD3B-D
d21	snoU13|ENSG00000238693.1	snoU13-D

Dictionary of important CNVs. Copy number events that are within any of the best K rule sets, or within the generalized core rules, or among the 20 most frequent events.
