# Supplementary material for: Identifying modules of cooperating cancer drivers
Source: Mol Syst Biol. 2021 Mar 26;17(3):e9810. doi: 10.15252/msb.20209810 (PMC7995435; doi:10.15252/msb.20209810)
Supplement: Supplementary file 13 — Dataset EV12 [file MSB-17-e9810-s004.zip › DATASET EV12/CRSO_Report_LUSC.pdf]

CRSO Output Report: LUSC

Michael Klein

Nov 11 2020

Contents

1 Dataset Overview and Parameters **2**
1.1 Parameter table 2
1.2 Timing breakdown (in minutes) 2

2 Heatmaps of D and P **3**

3 Summary of K Best Rule Sets **4**
3.1 Performance and coverage of best rule sets 4
3.2 Table of rules that appear in any best rule set 5

4 Core Rule Set **6**
4.1 Table of core rule set rules 6
4.2 Event breakdown of core rule set 7
4.3 Core Rule Set Penalties: Before and After Assignment 8

5 Generalized Core Analysis **9**

6 Combined Core Table **13**

7 Dictionary of Copy Number Events **14**

1 Dataset Overview and Parameters

Number of samples = 178.
Number of events = 112.
Rule coverage requirement = 8 samples.
Rule assignment requirement = 6 samples.
Rule library size = 1588 rules.

1.1 Parameter table

Parameter Name	Value	Description	Category
n.cores	20	Number HPC Cores	Resources
msa	6	Minimum Samples Assigned	RS Constraint
rule.thresh	0.0449	Rule Coverage Thresh	Library Definition
max.rl	2000	Max Rule Library Size	Library Definition
p1.ntpr	40	P1 Num Trial Per Rule	Phase 1
p1.stop	24	P1 Stop Elimination	Phase 1
cut.size	0.25	P1 Cut Size	Phase 1
k.max.2	10	P2 Max K	Phase 2
max.nrs.p2	200000	P2 Max RS Evaluated Per K	Phase 2
Total Time	113	P2 Max RS Family Check Per K	Phase 2
max.stored.p2	10	P2 Num Top RS Stored Per K	Phase 2
max.nrs.p3	200000	P3 Max RS Evaluated Per K	Phase 3
max.stored.p3	100	P3 Num Top RS Stored Per K	Phase 3
k.max.4	40	P4 Max K	Phase 4
max.nrs.p4	100000	P4 Max RS Evaluated	Phase 4
max.stored.p4	100	P4 Num Top RS Stored Per K	Phase 4
gc.iter	100	Num CG Iterations	Generalized Core
gc.eval	100	Num RS Per GC	Generalized Core
r.seed	111	Total Computational Time	Timing

1.2 Timing breakdown (in minutes)

P1	PS	P2	P3	P4	GC	Total
7.43	8.28	8.36	16.5	32.2	39.9	113

2 Heatmaps of D and P

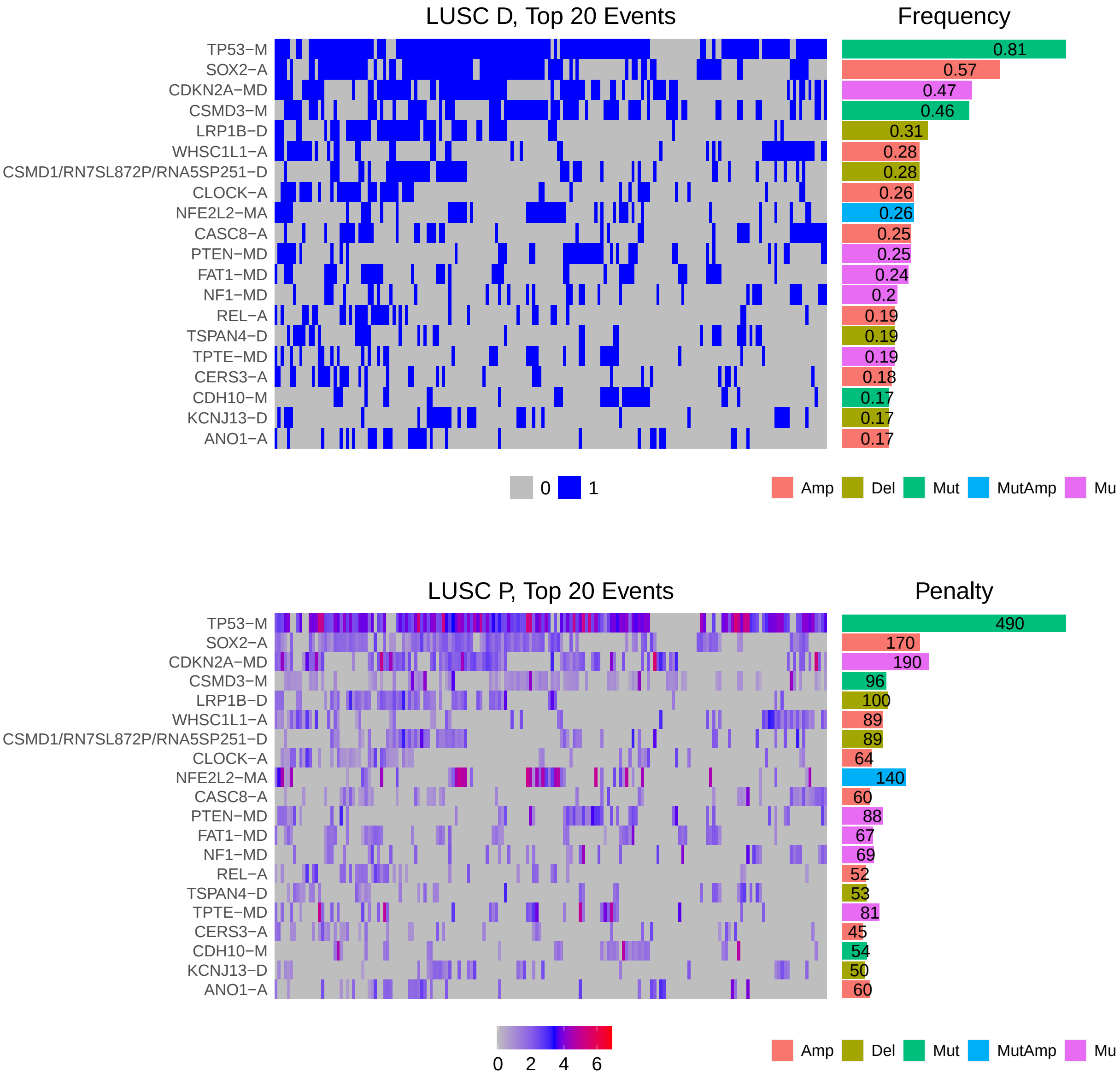

Figure 1: Heatmap of D and P. Top: binary representation of top 20 most frequent events. Top right shows event types and frequencies across the population. Bottom: Penalty matrix for top 20 most frequent events. Bottom right shows total event penalties. Events are ordered by frequency, from top to bottom. Samples are ordered using hierarchical clustering. Wild-type events indicated in grey. Event suffix -M: mutation, -A: amplification, -D: deletion, -MD: mutDel, -MA: mutAmp.

3 Summary of K Best Rule Sets

3.1 Performance and coverage of best rule sets

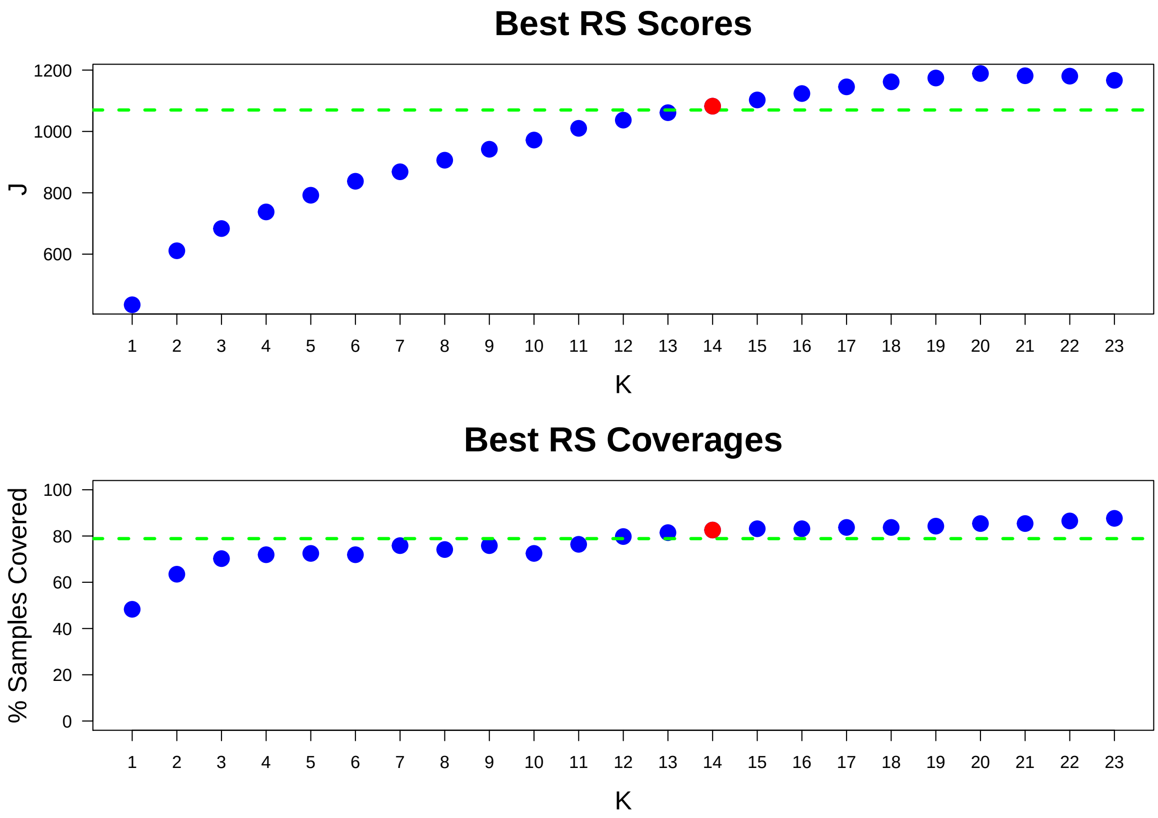

Figure 2: Performance and Coverage of Best Filtered RS for K. The core was determined to be the smallest RS that achieved at least 90% of maximum coverage and at least 90% of maximum performance.

3.2 Table of rules that appear in any best rule set

IR	Rule	PC	Ks
3	TP53-M+WHSC1L1-A	21	3-23
17	FOXP1/MIR1284-D+MIR3923/RN7SL751P/ROBO1-D+PROS1/STX19-D+ROBO2-D+TP53-M	6.7	6-23
30	CSMD3-M+NFE2L2-MA+SOX2-A	8.4	8-23
10	RB1-MD+SOX2-A+TP53-M	8.4	8-22
11	PIK3CA-M+SOX2-A+TP53-M	8.4	10-20
21	TP53-M+TSPAN4-D	16	10-12,15-23
12	CDH10-M+TP53-M	16	11-12,15-21
16	NF1-MD+TP53-M	16	8-13,15-19
18	CDKN2A-MD+EGFR-A+LRP1B-D+SOX2-A+TP53-M	6.2	13-23
46	CDKN2A-MD+LRP1B-D+NFE2L2-MA+SOX2-A+TP53-M	4.5	15-23
167	CDKN2A-MD+LRP1B-D+PLEKHO1/VPS45-A+TP53-M	6.2	15-23
77	ANO1-A+CSMD1/RN7SL872P/RNA5SP251-D+CSMD3-M+TP53-M	5.1	16-23
6	CDKN2A-MD+NFE2L2-MA+TP53-M	12	8-14
7	CSMD3-M+TP53-M	36	6-9,13-15
20	CDKN2A-MD+PTEN-MD+TP53-M	12	15-21
63	KEAP1-M+SOX2-A+TP53-M	7.9	17-23
75	CDKN2A-MD+CSMD3-M	22	16-18,20-23
81	ANO1-A+CDKN2A-MD	10	16-21
109	CSMD3-M+NFE2L2-MA+TP53-M+TPTE-MD	4.5	17-23
1	CDKN2A-MD+TP53-M	39	2-7
71	CDKN2A-MD+WHSC1L1-A	13	12-15
230	ATP8B5P/UNC13B-A+CLOCK-A+SOX2-A+TP53-M	4.5	18-23
2	SOX2-A+TP53-M	48	1-4
66	CLOCK-A+SOX2-A+TP53-M	14	13-16
74	CASC8-A+NF1-MD+TP53-M	6.2	20-23
166	FAT1-MD+SOX2-A	15	20-23
5	CDKN2A-MD+LRP1B-D+SOX2-A+TP53-M	13	10-12
19	LRP1B-D+SOX2-A+TP53-M	22	5-7
26	CDKN2A-MD+CSMD3-M+TP53-M	18	10-12
37	CDC42EP4-A+TP53-M	13	13-14
44	CERS3-A+TP53-M	15	22-23
85	FAT1-MD+NFE2L2-MA+TP53-M	8.4	20-22
4	CDKN2A-MD+SOX2-A+TP53-M	24	8-9
9	CSMD3-M+SOX2-A+TP53-M	22	
15	NFE2L2-MA+SOX2-A+TP53-M	15	6-7
34	CDKN2A-MD+TP53-M+TPTE-MD	9	13-14
41	PIK3CA-M+TP53-M	11	
59	CSMD3-M+PTEN-MD+TP53-M	11	22-23
91	FOXP1/MIR1284-D+MIR3923/RN7SL751P/ROBO1-D+ROBO2-D+SOX2-A+TP53-M	5.1	
103	PTEN-MD+RB1-MD+TP53-M	4.5	22-23
8	NFE2L2-MA+TP53-M	22	4
36	KCNJ13-D+TP53-M	16	23
38	CDKN2A-MD+NF1-MD+TP53-M	8.4	14
64	CDKN2A-MD+CSMD1/RN7SL872P/RNA5SP251-D+TP53-M	13	23
65	CASC8-A+CDKN2A-MD+TP53-M	11	19
398	a5(n=16)+CSMD3-M	11	19

ID = Rule IDs, rules are numbered according to importance rank determined from phase 1
PC = Percent of samples covered **Ks** = Membership in best RS

4 Core Rule Set

Core K = 14.
Core rule set coverage = 82.6%.

4.1 Table of core rule set rules

IR	Rule	CR	SJR	SJ	NSC	NSA	PC	PA	FracA
3	TP53-M + WHSC1L1-A	16	17	193	38	14	21	7.9	0.37
6	CDKN2A-MD + NFE2L2-MA + TP53-M	69	18	185	22	15	12	8.4	0.68
7	CSMD3-M + TP53-M	3	4	284	64	15	36	8.4	0.23
10	RB1-MD + SOX2-A + TP53-M	230	59.5	127	15	12	8.4	6.7	0.80
11	PIK3CA-M + SOX2-A + TP53-M	230	63.5	125	15	13	8.4	7.3	0.87
17	FOXP1/MIR1284-D + MIR3923/RN7SL751P/ROBO1-D + PROS1/STX19-D + ROBO2-D + TP53-M	439.5	111	106	12	10	6.7	5.6	0.83
18	CDKN2A-MD + EGFR-A + LRP1B-D + SOX2-A + TP53-M	547	84.5	112	11	11	6.2	6.2	1.00
30	CSMD3-M + NFE2L2-MA + SOX2-A	230	126	102	15	7	8.4	3.9	0.47
34	CDKN2A-MD + TP53-M + TPTE-MD	185.5	46	133	16	10	9	5.6	0.62
37	CDC42EP4-A + TP53-M	57	94.5	110	23	8	13	4.5	0.35
38	CDKN2A-MD + NF1-MD + TP53-M	230	105.5	107	15	9	8.4	5.1	0.60
44	CERS3-A + TP53-M	37.5	51	131	27	6	15	3.4	0.22
66	CLOCK-A + SOX2-A + TP53-M	45.5	31.5	151	25	10	14	5.6	0.40
71	CDKN2A-MD + WHSC1L1-A	57	210	88.3	23	7	13	3.9	0.30

ID = Rule IDs, rules are numbered according to importance rank determined from phase 1
CR = Coverage rank
SJ/SJR = Single rule performance / performance rank
NSC/PC = Number/percent of samples covered
NSA/PA = Number/percent of samples assigned
FracA = Fraction of covered samples assigned to rule

4.2 Event breakdown of core rule set

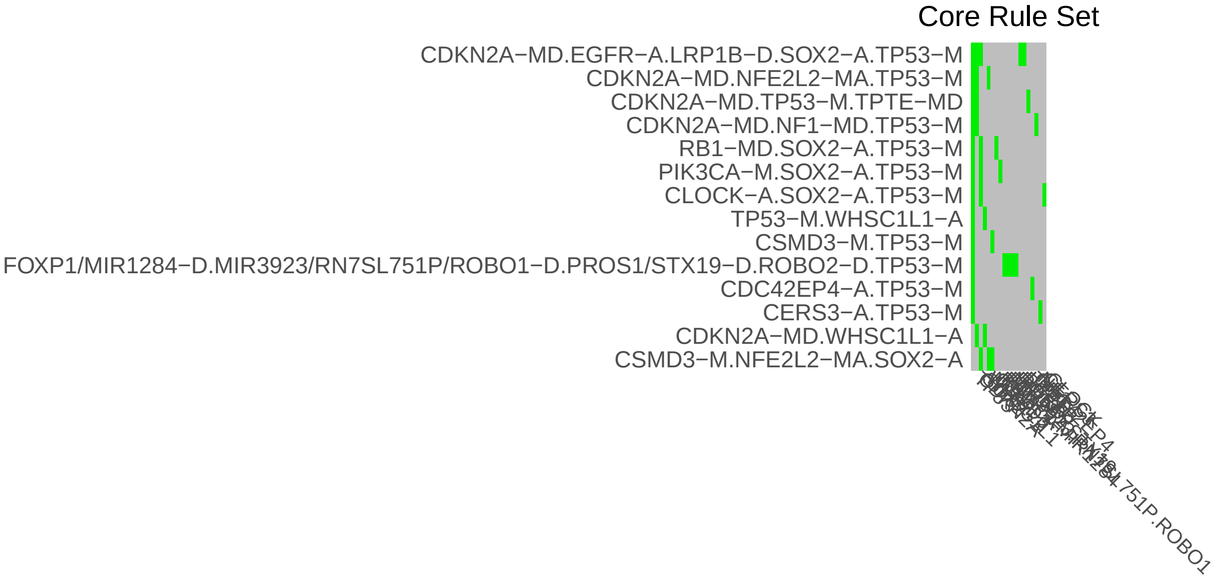

Figure 3: Visualization of core rule set. Rows are rules, columns are events. Events are ordered from left in decreasing rule membership frequency.

4.3 Core Rule Set Penalties: Before and After Assignment

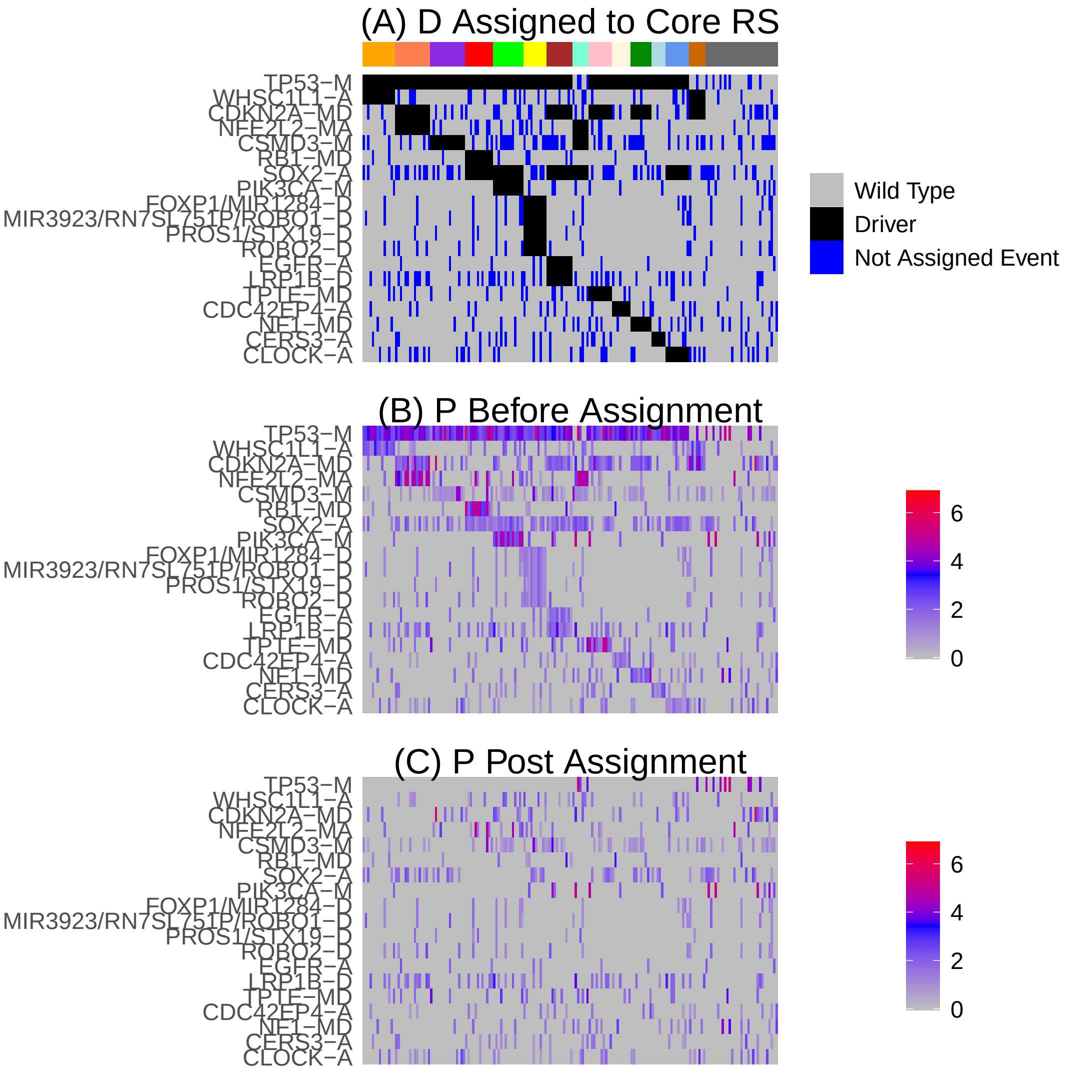

Figure 4: Heatmaps of D and P under core rule set assignment. Events are ordered by frequency. Samples are ordered according rule set membership, as indicated by the color bar. The dark grey group of samples are not assigned to any rule. A) For each sample, assigned events are designated as drivers and are shown in black, unassigned events are shown in blue. B-C) Heatmap of P before and after assignment to core rule set.

5 Generalized Core Analysis

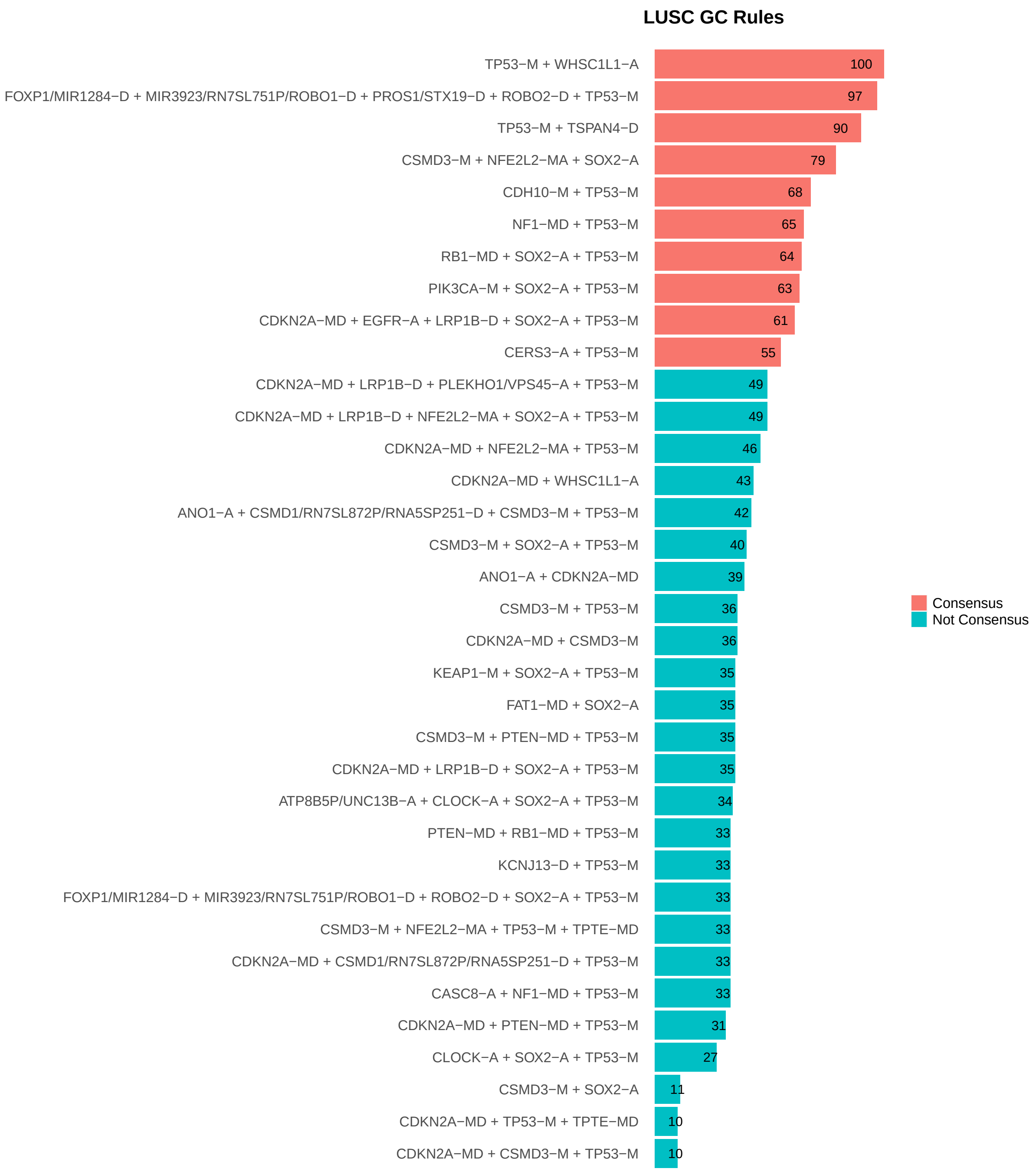

Figure 5: GCRs

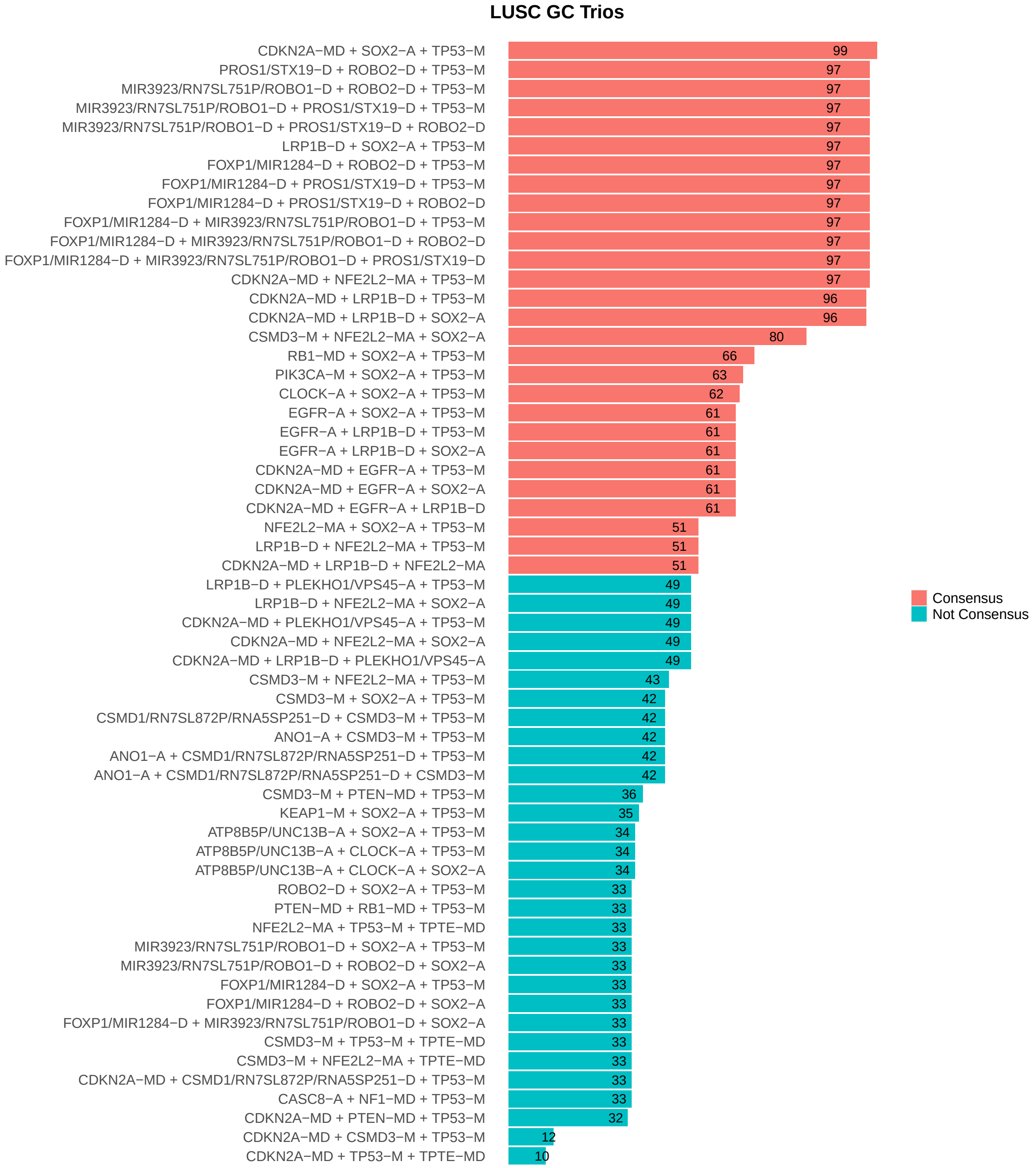

Figure 6: GCTs

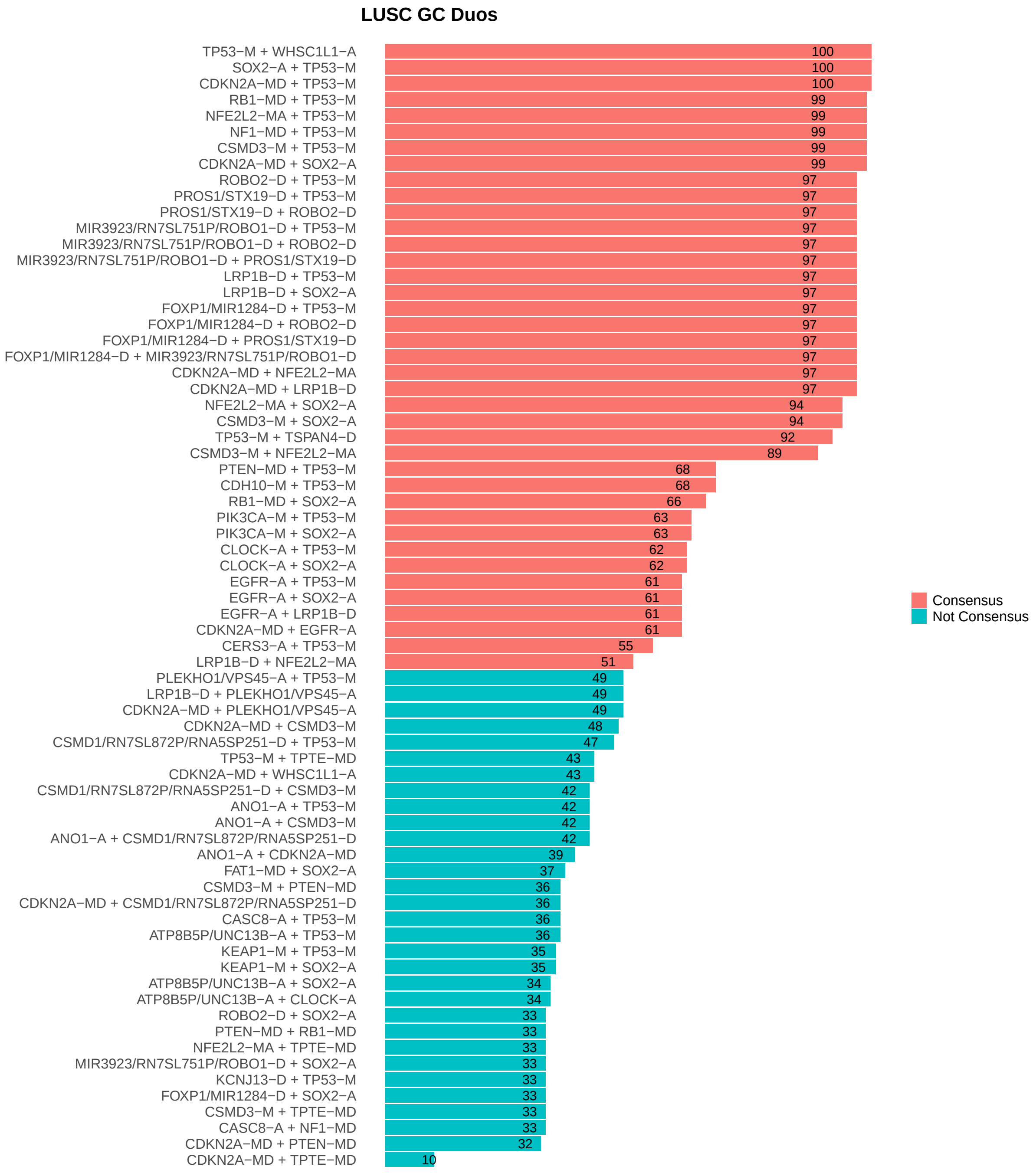

Figure 7: GCDs

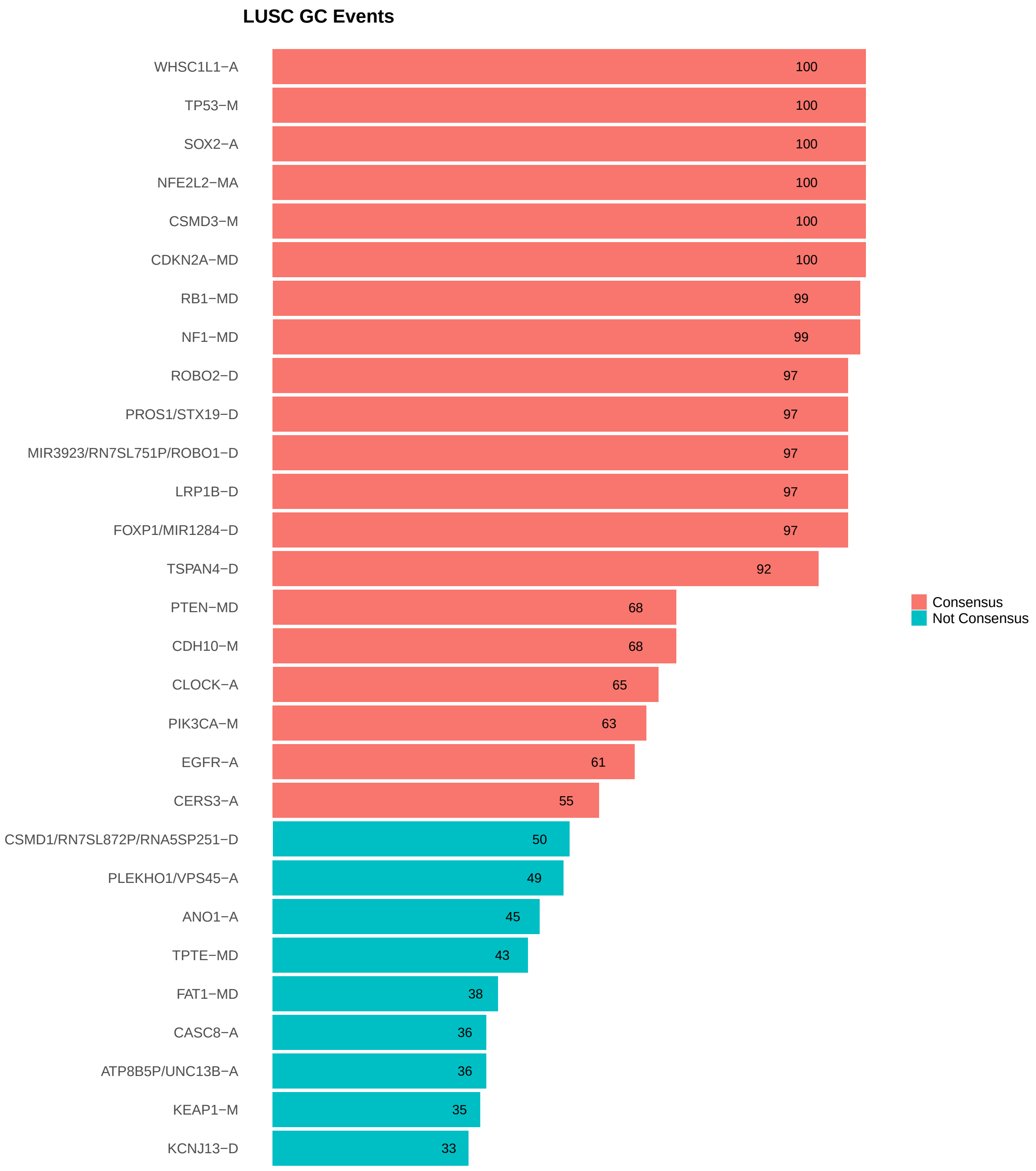

Figure 8: GCEs

6 Combined Core Table

Rule	Core_Type	P1_Rank	Confidence	Coverage	SJ	Fraction_Assigned
TP53-M + WHSC1L1-A	Both	3	100	21.3% (r=16)	299 (r=17)	0.71
FOXP1/MIR1284-D + MIR3923/RN7SL751P/ROBO1-D + PROS1/STX19-D + ROBO2-D + TP53-M	Both	17	97	6.74% (r=456)	193 (r=111)	0.17
CSMD3-M + NFE2L2-MA + SOX2-A	Both	30	79	8.43% (r=230)	152 (r=126)	0.2
RB1-MD + SOX2-A + TP53-M	Both	10	64	8.43% (r=251)	218 (r=59)	0.4
PIK3CA-M + SOX2-A + TP53-M	Both	11	63	8.43% (r=252)	216 (r=64)	0.53
CDKN2A-MD + EGFR-A + LRP1B-D + SOX2-A + TP53-M	Both	18	61	6.18% (r=605)	185 (r=84)	0.27
CERS3-A + TP53-M	Both	44	55	15.2% (r=37)	135 (r=50)	0.44
CDKN2A-MD + NFE2L2-MA + TP53-M	Core	6	46	12.4% (r=73)	254 (r=18)	0.59
CDKN2A-MD + WHSC1L1-A	Core	71	43	12.9% (r=55)	119 (r=210)	0.35
CSMD3-M + TP53-M	Core	7	36	36% (r=3)	252 (r=4)	0.42
CLOCK-A + SOX2-A + TP53-M	Core	66	27	14% (r=46)	124 (r=32)	0.28
CDKN2A-MD + TP53-M + TPTE-MD	Core	34	10	8.99% (r=200)	149 (r=47)	0.56
CDC42EP4-A + TP53-M	Core	37	4	12.9% (r=61)	145 (r=93)	0.65
CDKN2A-MD + NF1-MD + TP53-M	Core	38	1	8.43% (r=250)	144 (r=105)	0.47
TP53-M + TSPAN4-D	conGCR	21	90	15.7% (r=33)	172 (r=35)	-
CDH10-M + TP53-M	conGCR	12	68	16.3% (r=30)	212 (r=34)	-
NF1-MD + TP53-M	conGCR	16	65	15.7% (r=34)	194 (r=38)	-

7 Dictionary of Copy Number Events

CNV	Genes	Event__Name
a2	CASC8	CASC8-A
a3	WHSC1L1	WHSC1L1-A
a4	CLOCK	CLOCK-A
a5	ZDHHC11B, CCDC127, LRRC14B, PLEKHG4B, SDHA, SLC9A3, TRIP13, PDCD6, TPPP, EXOC3, CEP72, AHRR, BRD9, ZDHHC11, C5orf55, MIR4456	a5(n=16)
a6	ANO1	ANO1-A
a7	KCNK6, SPINT2, CATSPERG, C19orf33, YIF1B, PPP1R14A	a7(n=6)
a8	UNC13B, ATP8B5P	ATP8B5P/UNC13B-A
a9	CCNE1	CCNE1-A
a11	CERS3	CERS3-A
a12	REL	REL-A
a13	VPS45, PLEKHO1	PLEKHO1/VPS45-A
a14	CDC42EP4	CDC42EP4-A
a21	EGFR	EGFR-A
d2	LRP1B	LRP1B-D
d3	RN7SL872P, RNA5SP251, CSMD1	CSMD1/RN7SL872P/RNA5SP251-D
d4	KCNJ13	KCNJ13-D
d5	TSPAN4	TSPAN4-D
d6	FOXP1, MIR1284	FOXP1/MIR1284-D
d7	ROBO2	ROBO2-D
d10	RN7SL751P, ROBO1, MIR3923	MIR3923/RN7SL751P/ROBO1-D
d16	RN7SL5P, SNORD27|ENSG00000251699.1, PTPRD	PTPRD/RN7SL5P/SNORD27-D
d21	PROS1, STX19	PROS1/STX19-D

Dictionary of important CNVs. Copy number events that are within any of the best K rule sets, or within the generalized core rules, or among the 20 most frequent events.
