# Supplementary material for: Identifying modules of cooperating cancer drivers
Source: Mol Syst Biol. 2021 Mar 26;17(3):e9810. doi: 10.15252/msb.20209810 (PMC7995435; doi:10.15252/msb.20209810)
Supplement: Supplementary file 14 — Dataset EV13 [file MSB-17-e9810-s021.zip › DATASET EV13/CRSO_Report_OV.pdf]

CRSO Output Report: OV

Michael Klein

Nov 11 2020

Contents

1 Dataset Overview and Parameters **2**
1.1 Parameter table 2
1.2 Timing breakdown (in minutes) 2

2 Heatmaps of D and P **3**

3 Summary of K Best Rule Sets **4**
3.1 Performance and coverage of best rule sets 4
3.2 Table of rules that appear in any best rule set 5

4 Core Rule Set **6**
4.1 Table of core rule set rules 6
4.2 Event breakdown of core rule set 7
4.3 Core Rule Set Penalties: Before and After Assignment 8

5 Generalized Core Analysis **9**

6 Combined Core Table **13**

7 Dictionary of Copy Number Events **14**

1 Dataset Overview and Parameters

Number of samples = 455.
Number of events = 77.
Rule coverage requirement = 34 samples.
Rule assignment requirement = 14 samples.
Rule library size = 1990 rules.

1.1 Parameter table

Parameter Name	Value	Description	Category
n.cores	20	Number HPC Cores	Resources
msa	14	Minimum Samples Assigned	RS Constraint
rule.thresh	0.0747	Rule Coverage Thresh	Library Definition
max.rl	2000	Max Rule Library Size	Library Definition
p1.ntpr	40	P1 Num Trial Per Rule	Phase 1
p1.stop	24	P1 Stop Elimination	Phase 1
cut.size	0.25	P1 Cut Size	Phase 1
k.max.2	10	P2 Max K	Phase 2
max.nrs.p2	200000	P2 Max RS Evaluated Per K	Phase 2
Total Time	203	P2 Max RS Family Check Per K	Phase 2
max.stored.p2	10	P2 Num Top RS Stored Per K	Phase 2
max.nrs.p3	200000	P3 Max RS Evaluated Per K	Phase 3
max.stored.p3	100	P3 Num Top RS Stored Per K	Phase 3
k.max.4	40	P4 Max K	Phase 4
max.nrs.p4	100000	P4 Max RS Evaluated	Phase 4
max.stored.p4	100	P4 Num Top RS Stored Per K	Phase 4
gc.iter	100	Num CG Iterations	Generalized Core
gc.eval	100	Num RS Per GC	Generalized Core
r.seed	111	Total Computational Time	Timing

1.2 Timing breakdown (in minutes)

P1	PS	P2	P3	P4	GC	Total
41.9	7.44	10.7	33.9	59.7	49.8	203

2 Heatmaps of D and P

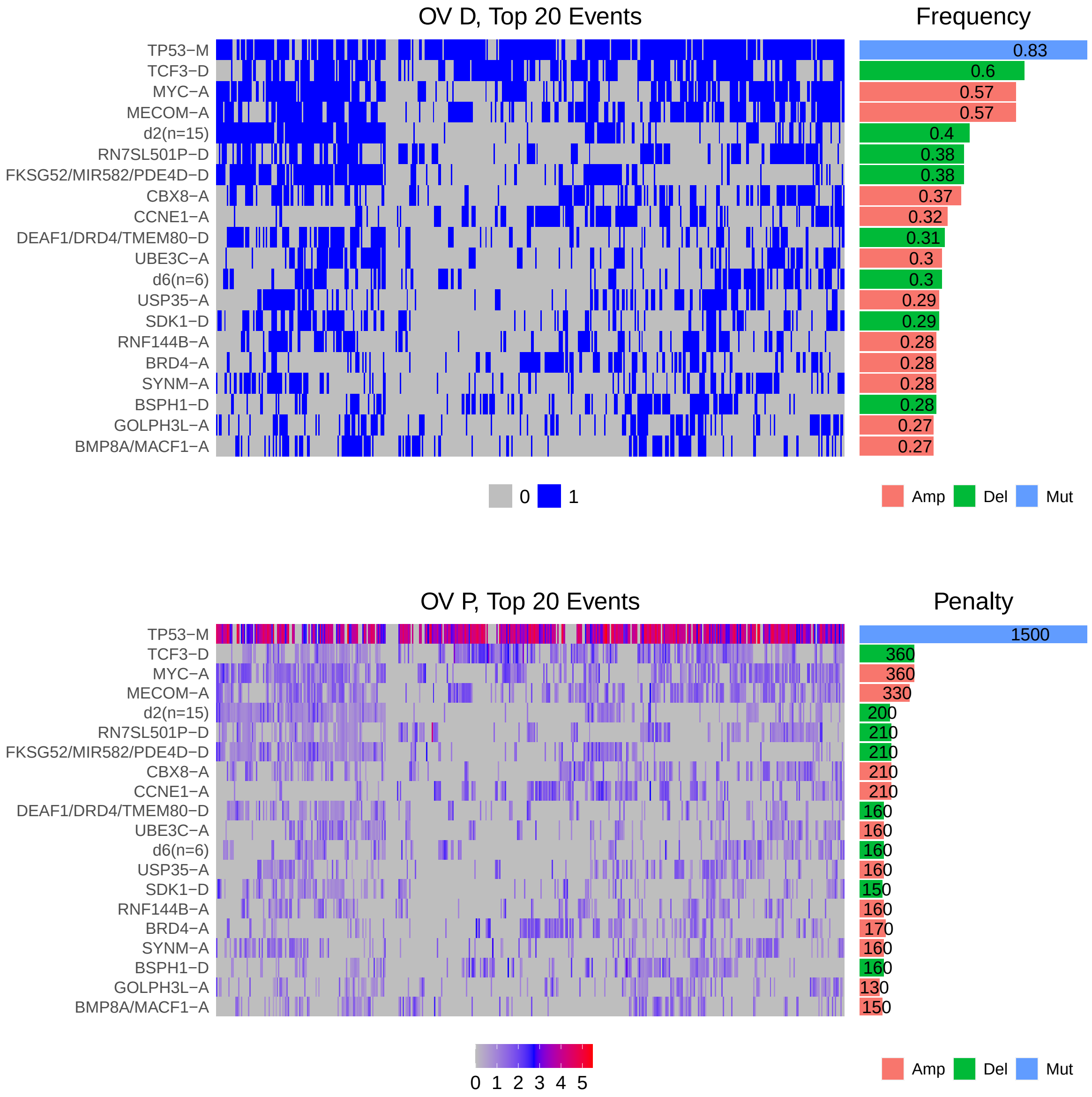

Figure 1: Heatmap of D and P. Top: binary representation of top 20 most frequent events. Top right shows event types and frequencies across the population. Bottom: Penalty matrix for top 20 most frequent events. Bottom right shows total event penalties. Events are ordered by frequency, from top to bottom. Samples are ordered using hierarchical clustering. Wild-type events indicated in grey. Event suffix -M: mutation, -A: amplification, -D: deletion, -MD: mutDel, -MA: mutAmp.

3 Summary of K Best Rule Sets

3.1 Performance and coverage of best rule sets

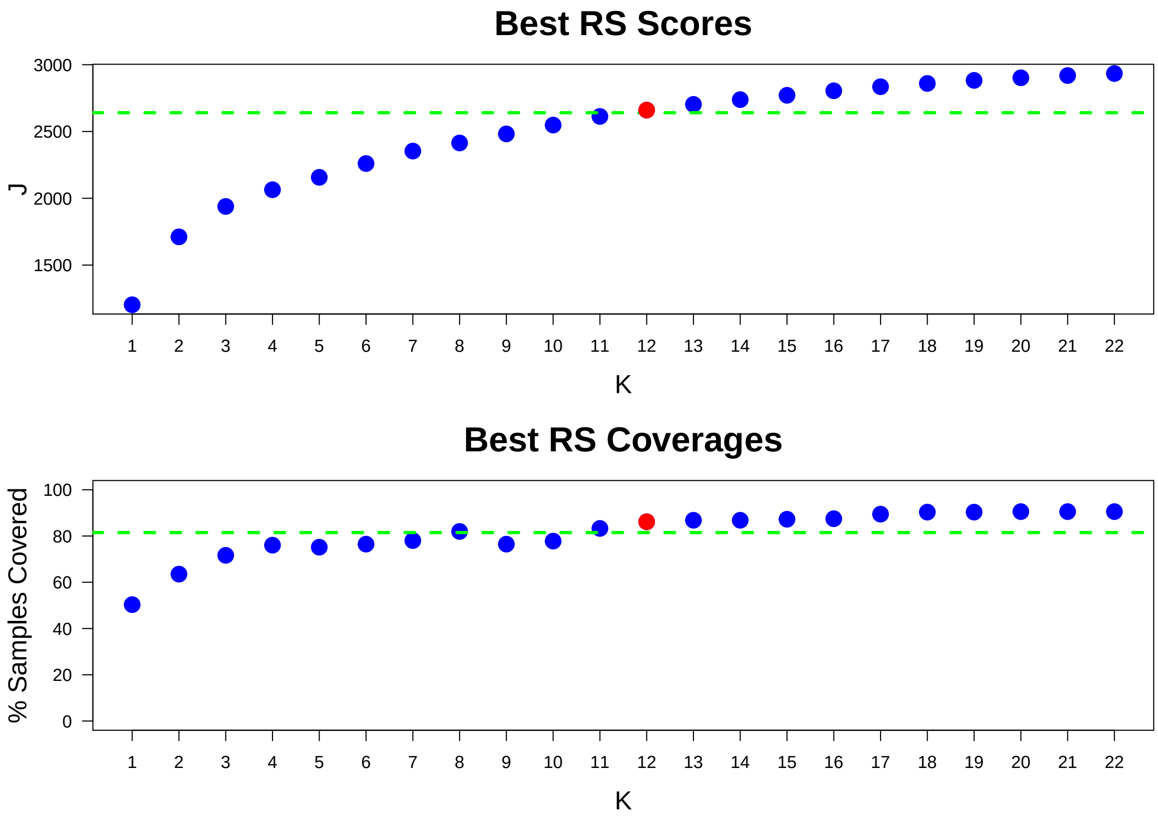

Figure 2: Performance and Coverage of Best Filtered RS for K. The core was determined to be the smallest RS that achieved at least 90% of maximum coverage and at least 90% of maximum performance.

3.2 Table of rules that appear in any best rule set

IR	Rule	PC	Ks
5	RN7SL501P-D+TP53-M	31	6-22
6	BSPH1-D+RN7SL526P-D+TCF3-D+TP53-M	12	6-22
11	BRD4-A+TP53-M	24	6-22
13	MYC-A+TCF3-D+TP53-M	31	6-18,20-22
15	d15(n=5)+PPP2R2A-D+TP53-M	13	9-22
8	d2(n=15)+FKSG52/MIR582/PDE4D-D+MECOM-A+MYC-A+TP53-M	13	9-22
76	d6(n=6)+MECOM-A+MYC-A+TP53-M	13	9-22
117	CBX8-A+MYC-A	25	11-22
275	d2(n=15)+FKSG52/MIR582/PDE4D-D+TCF3-D	18	12-16,18-22
9	MECOM-A+TCF3-D+TP53-M	29	9-16
14	d9(n=7)+TP53-M	21	14-22
36	CCNE1-A+MECOM-A+RN7SL566P/SAMD4B-A+TP53-M	8.4	14-22
94	d2(n=15)+DEAF1/DRD4/TMEM80-D+FKSG52/MIR582/PDE4D-D+MYC-A+TP53-M	10	14-22
55	ANKS1B/FAM71C/RNA5SP366-D+d13(n=19)+TP53-M	13	10-15
31	CCNE1-A+RN7SL566P/SAMD4B-A+TP53-M	13	7-13
75	CCNE1-A+RN7SL566P/SAMD4B-A+TCF3-D+TP53-M	7.5	16-22
2	MECOM-A+TP53-M	49	3-8
7	d2(n=15)+FKSG52/MIR582/PDE4D-D+MYC-A+TP53-M	18	3-8
77	MECOM-A+TP53-M+UBE3C-A	18	16-18,20-22
1	TCF3-D+TP53-M	50	1-5
30	MECOM-A+TCF3-D	34	17-18,20-22
33	d28(n=6)+MGA/MIR626-D+TP53-M	11	17-18,20-22
108	ANKS1B/FAM71C/RNA5SP366-D+d13(n=19)+MYC-A+TP53-M	8.6	20-22
50	COA6-A+TCF3-D+TP53-M	13	20-22
164	d2(n=15)+FKSG52/MIR582/PDE4D-D+SYNM-A+TP53-M	8.4	20-22
175	MECOM-A+MYC-A+NF1-MD+TP53-M	7.9	20-22
120	BMP8A/MACF1-A+MECOM-A+MYC-A+TP53-M	11	21-22
246	KLLN/PTEN-D+RNA5SP325-D+TCF3-D+TP53-M	7.5	
4	MECOM-A+MYC-A+TP53-M	33	2
16	CCNE1-A+TP53-M	27	4
20	BRD4-A+CCNE1-A+TP53-M	12	5
99	d15(n=5)+MYC-A+TCF3-D+TP53-M	9.9	19
104	RB1-MD+TCF3-D+TP53-M	12	17
129	MYC-A+SDK1-D+TP53-M	18	19
131	d2(n=15)+FKSG52/MIR582/PDE4D-D+TP53-M+USP35-A	9.2	13
250	d2(n=15)+MECOM-A+MYC-A+TP53-M+UBE3C-A	8.1	19
311	DEAF1/DRD4/TMEM80-D+MECOM-A	20	19
449	CBX8-A+d6(n=6)+TCF3-D+TP53-M	7.7	22

ID = Rule IDs, rules are numbered according to importance rank determined from phase 1
PC = Percent of samples covered **Ks** = Membership in best RS

4 Core Rule Set

Core K = 12.
Core rule set coverage = 86.2%.

4.1 Table of core rule set rules

IR	Rule	CR	SJR	SJ	NSC	NSA	PC	PA	FracA
5	RN7SL501P-D + TP53-M	9	8	717	143	26	31	5.7	0.18
6	BSPH1-D + RN7SL526P-D + TCF3-D + TP53-M	460.5	85	441	54	42	12	9.2	0.78
8	d2(n=15) + FKSG52/MIR582/PDE4D-D + MECOM-A + MYC-A + TP53-M	329.5	39	520	60	56	13	12.0	0.93
9	MECOM-A + TCF3-D + TP53-M	13.5	6	862	133	39	29	8.6	0.29
11	BRD4-A + TP53-M	27.5	24	579	110	26	24	5.7	0.24
13	MYC-A + TCF3-D + TP53-M	11	5	895	139	37	31	8.1	0.27
15	d15(n=5) + PPP2R2A-D + TP53-M	386.5	160.5	374	57	32	13	7.0	0.56
31	CCNE1-A + RN7SL566P/SAMD4B-A + TP53-M	348	119	404	59	31	13	6.8	0.53
55	ANKS1B/FAM71C/RNA5SP366-D + d13(n=19) + TP53-M	366	206	350	58	26	13	5.7	0.45
76	d6(n=6) + MECOM-A + MYC-A + TP53-M	366	108	415	58	39	13	8.6	0.67
117	CBX8-A + MYC-A	24	388.5	289	114	23	25	5.1	0.20
275	d2(n=15) + FKSG52/MIR582/PDE4D-D + TCF3-D	97.5	431.5	279	84	15	18	3.3	0.18

ID = Rule IDs, rules are numbered according to importance rank determined from phase 1
CR = Coverage rank
SJ/SJR = Single rule performance / performance rank
NSC/PC = Number/percent of samples covered
NSA/PA = Number/percent of samples assigned
FracA = Fraction of covered samples assigned to rule

4.2 Event breakdown of core rule set

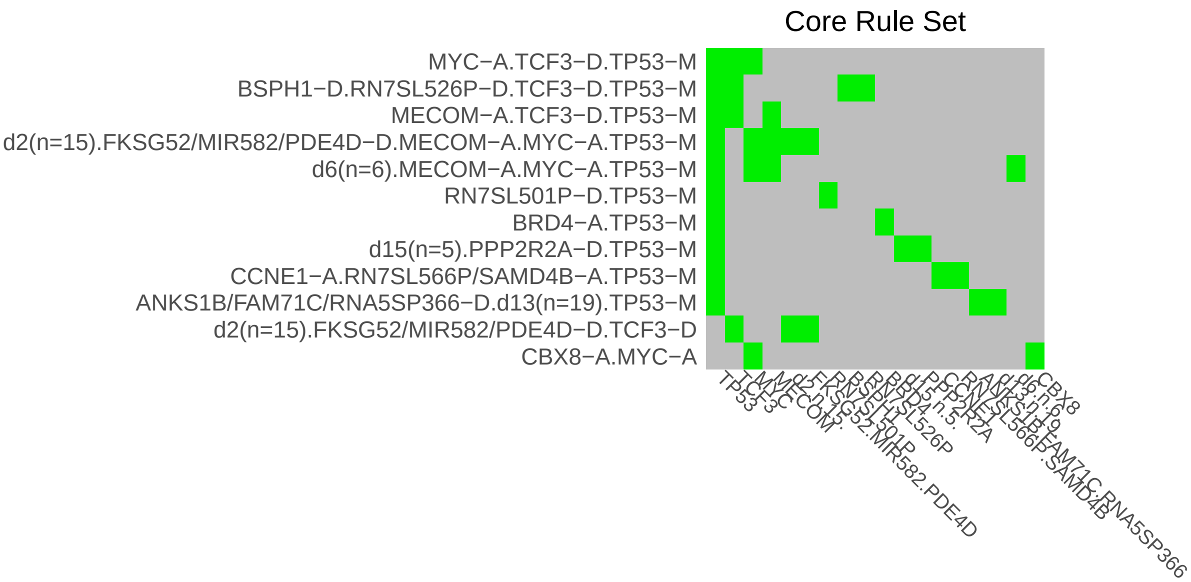

Figure 3: Visualization of core rule set. Rows are rules, columns are events. Events are ordered from left in decreasing rule membership frequency.

4.3 Core Rule Set Penalties: Before and After Assignment

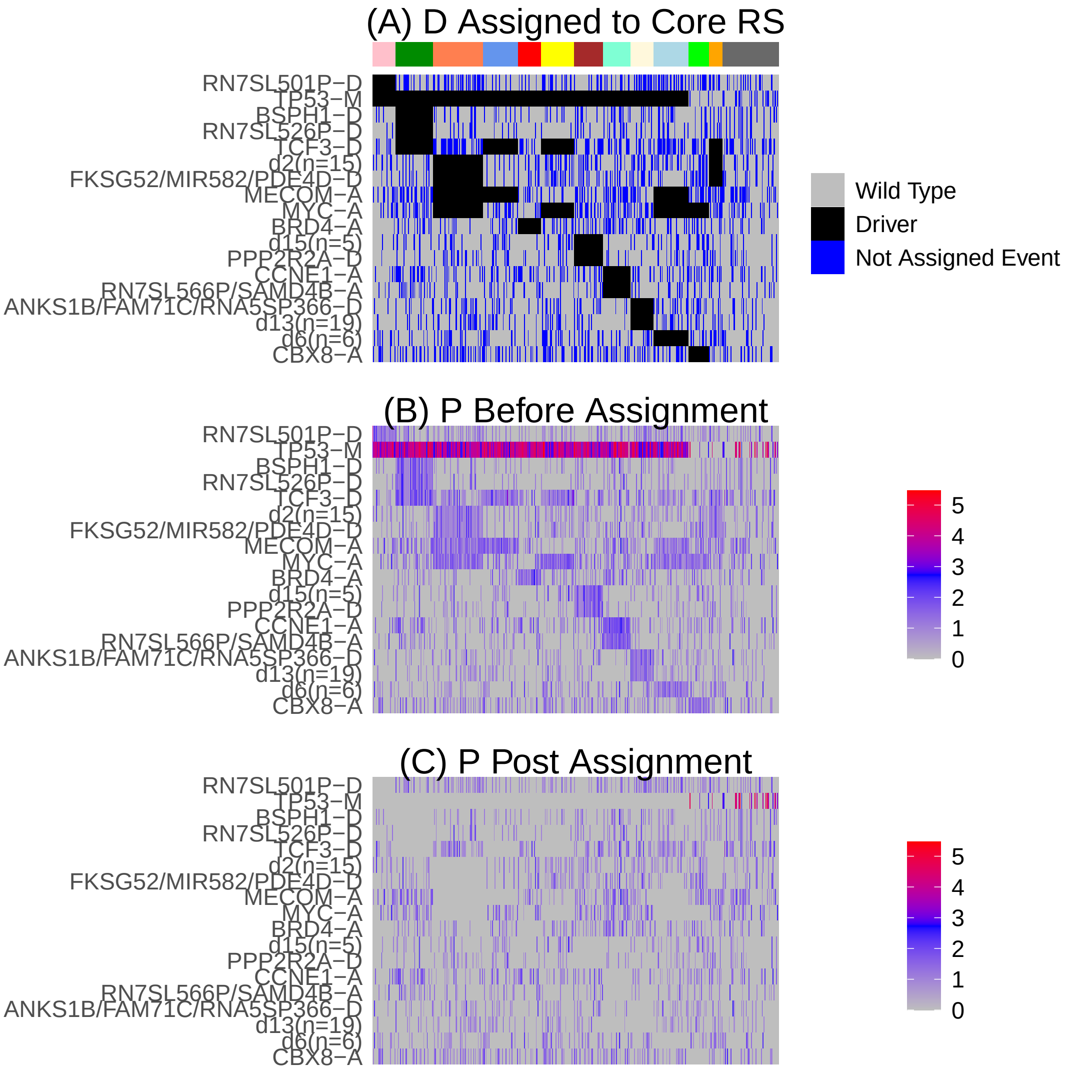

Figure 4: Heatmaps of D and P under core rule set assignment. Events are ordered by frequency. Samples are ordered according rule set membership, as indicated by the color bar. The dark grey group of samples are not assigned to any rule. A) For each sample, assigned events are designated as drivers and are shown in black, unassigned events are shown in blue. B-C) Heatmap of P before and after assignment to core rule set.

5 Generalized Core Analysis

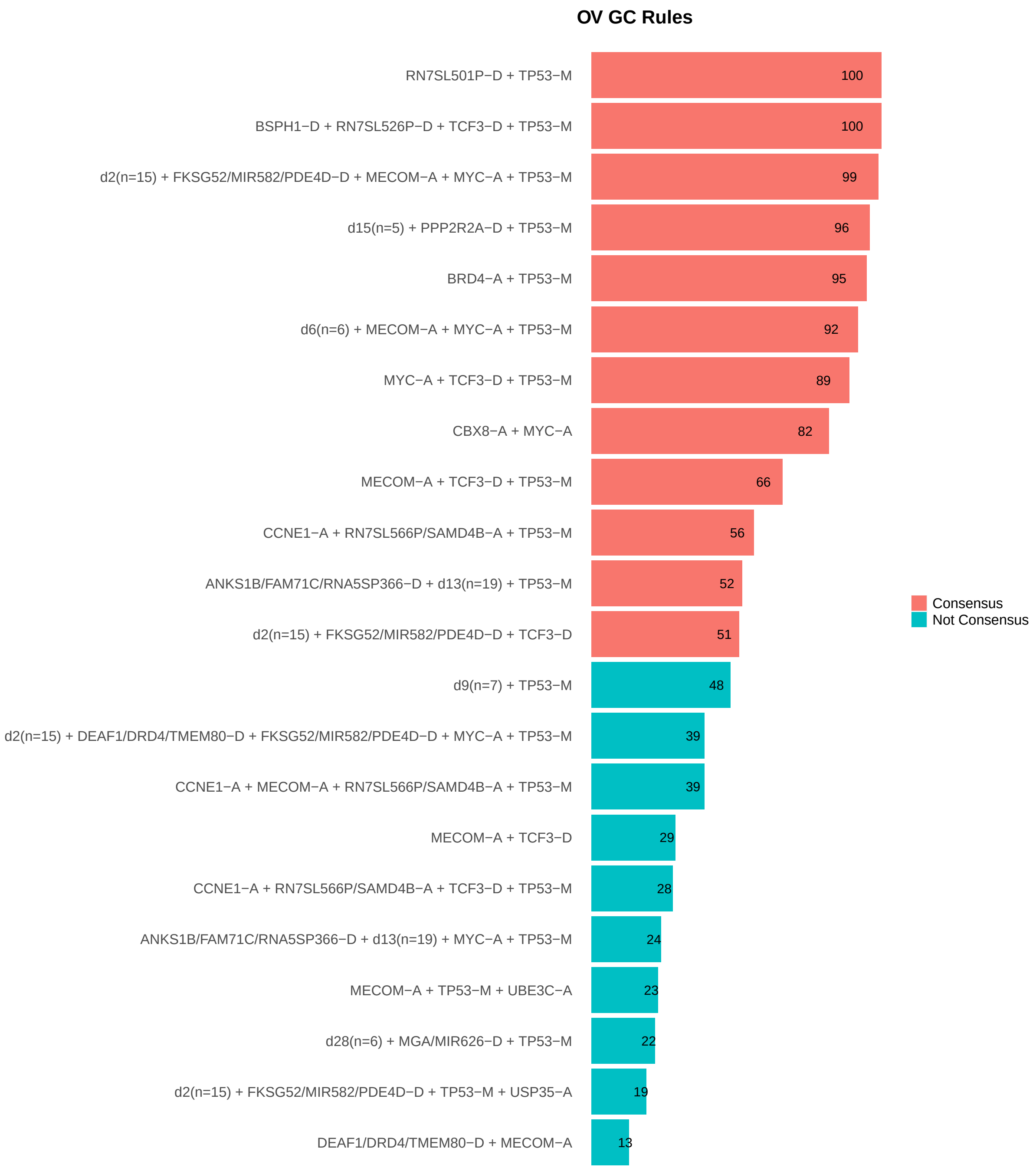

Figure 5: GCRs

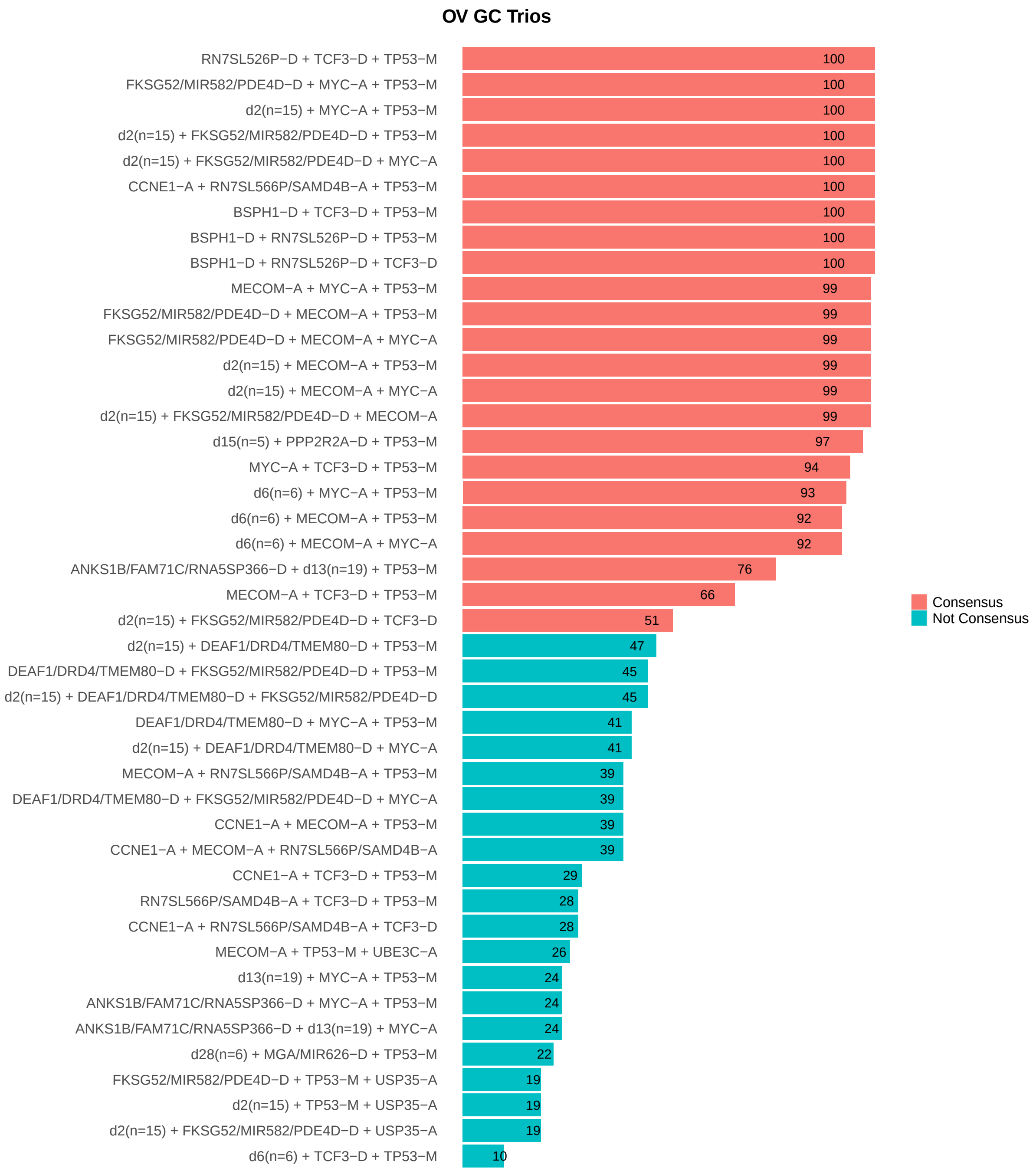

Figure 6: GCTs

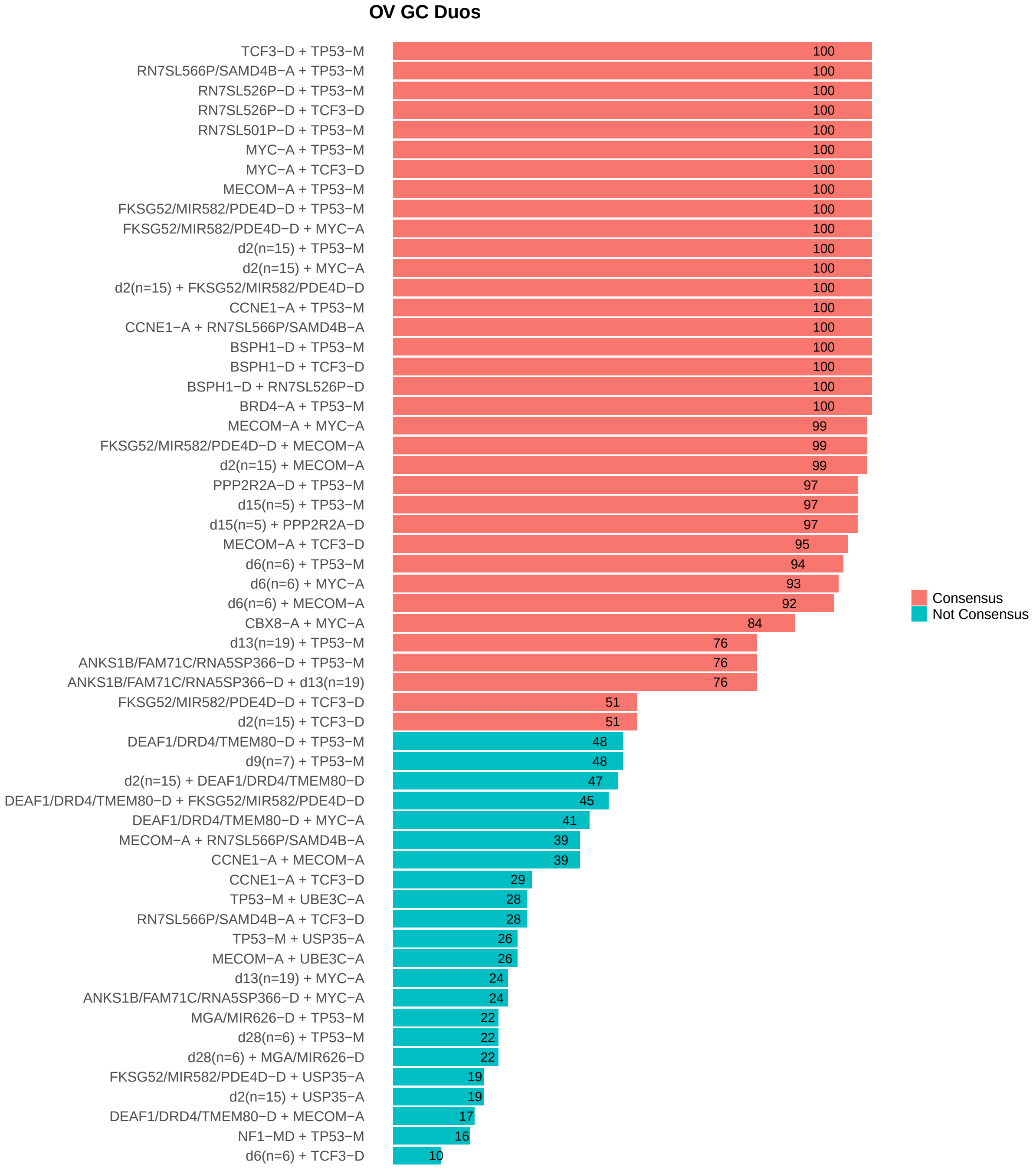

Figure 7: GCDs

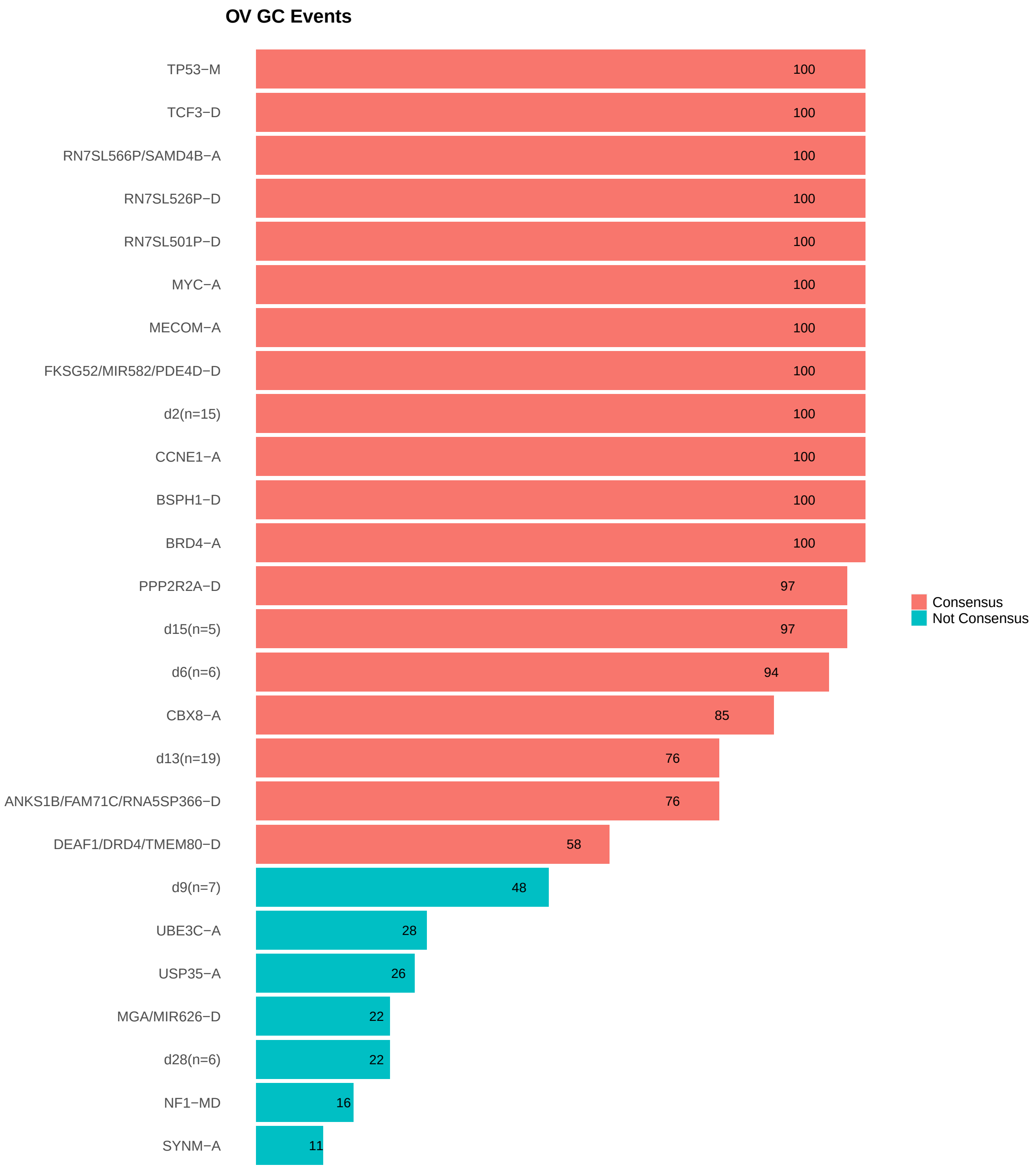

Figure 8: GCEs

6 Combined Core Table

Rule	Core_Type	P1_Rank	Confidence	Coverage	SJ	Fraction_Assigned
RN7SL501P-D + TP53-M	Both	5	100	31.4% (r=9)	895 (r=8)	0.750
BSPH1-D + RN7SL526P-D + TCF3-D + TP53-M	Both	6	100	11.9% (r=472)	862 (r=85)	0.200
d2(n=15) + FKSG52/MIR582/PDE4D-D + MECOM-A + MYC-A + TP53-M	Both	8	99	13.2% (r=339)	717 (r=39)	0.100
d15(n=5) + PPP2R2A-D + TP53-M	Both	15	96	12.5% (r=387)	617 (r=159)	0.400
BRD4-A + TP53-M	Both	11	95	24.2% (r=27)	687 (r=24)	0.730
d6(n=6) + MECOM-A + MYC-A + TP53-M	Both	76	92	12.7% (r=373)	456 (r=108)	0.069
MYC-A + TCF3-D + TP53-M	Both	13	89	30.5% (r=11)	674 (r=5)	0.160
CBX8-A + MYC-A	Both	117	82	25.1% (r=23)	405 (r=389)	0.440
MECOM-A + TCF3-D + TP53-M	Both	9	66	29.2% (r=14)	713 (r=6)	0.310
CCNE1-A + RN7SL566P/SAMD4B-A + TP53-M	Both	31	56	13% (r=349)	539 (r=119)	0.320
ANKS1B/FAM71C/RNA5SP366-D + d13(n=19) + TP53-M	Both	55	52	12.7% (r=367)	488 (r=208)	0.220
d2(n=15) + FKSG52/MIR582/PDE4D-D + TCF3-D	Both	275	51	18.5% (r=97)	321 (r=432)	0.190

7 Dictionary of Copy Number Events

CNV	Genes	Event_Name
a1	MYC	MYC-A
a2	MECOM	MECOM-A
a3	CBX8	CBX8-A
a4	CCNE1	CCNE1-A
a5	GOLPH3L	GOLPH3L-A
a6	USP35	USP35-A
a7	RNF144B	RNF144B-A
a8	BRD4	BRD4-A
a9	UBE3C	UBE3C-A
a10	BMP8A, MACF1	BMP8A/MACF1-A
a11	SYNM	SYNM-A
a12	COA6	COA6-A
a14	RN7SL566P, SAMD4B	RN7SL566P/SAMD4B-A
a19	MIR4635, TERT, SLC12A7, NKD2, SLC6A19, SLC6A18, MIR4457	a19(n=7)
d1	TCF3	TCF3-D
d2	snoU13|ENSG00000238451.1, GTF2H2B, RN7SL9P, snoU13|ENSG00000238740.1, GUSBP3, RN7SL616P, RN7SL476P, GTF2H2, NAIP, SMN1, SMN2, SERF1A, GTF2H2C, SERF1B, OCLN	d2(n=15)
d3	FKSG52, MIR582, PDE4D	FKSG52/MIR582/PDE4D-D
d4	RN7SL501P	RN7SL501P-D
d5	TMEM80, DRD4, DEAF1	DEAF1/DRD4/TMEM80-D
d6	TCP10, TTLL2, GPR31, C6orf123, UNC93A, TCP10L2	d6(n=6)
d7	SDK1	SDK1-D
d8	BSPH1	BSPH1-D
d9	LINC00901, TUSC7, RN7SL582P, LINC00903, RN7SL815P, LSAMP, MIR4447	d9(n=7)
d10	RN7SL526P	RN7SL526P-D
d12	PPP2R2A	PPP2R2A-D
d13	ANHX, ZNF891, ZNF140, RNU4ATAC12P, RNA5SP379, LRCOL1, GOLGA3, POLE, PXMP2, ZNF10, ZNF26, ZNF84, ZNF268, P2RX2, ANKLE2, CHFR, FBRSL1, PGAM5, ZNF605	d13(n=19)
d14	RNA5SP366, ANKS1B, FAM71C	ANKS1B/FAM71C/RNA5SP366-D
d15	RPL23AP53, OR4F21, FBXO25, TDRP, ZNF596	d15(n=5)
d20	RNA5SP325	RNA5SP325-D
d21	MIR626, MGA	MGA/MIR626-D
d24	PTEN, KLLN	KLLN/PTEN-D
d28	SNORD109B, SNORD115|ENSG00000212428.1, SNORD109A, SNORD108, SNORD64|ENSG00000270704.2, SNHG14	d28(n=6)

Dictionary of important CNVs. Copy number events that are within any of the best K rule sets, or within the generalized core rules, or among the 20 most frequent events.
