# Supplementary material for: Identifying modules of cooperating cancer drivers
Source: Mol Syst Biol. 2021 Mar 26;17(3):e9810. doi: 10.15252/msb.20209810 (PMC7995435; doi:10.15252/msb.20209810)
Supplement: Supplementary file 17 — Dataset EV16 [file MSB-17-e9810-s017.zip › DATASET EV16/CRSO_Report_READ.pdf]

CRSO Output Report: READ

Michael Klein

Nov 11 2020

Contents

1 Dataset Overview and Parameters **2**
1.1 Parameter table . . . 2
1.2 Timing breakdown (in minutes) . . . 2

2 Heatmaps of D and P **3**

3 Summary of K Best Rule Sets **4**
3.1 Performance and coverage of best rule sets . . . 4
3.2 Table of rules that appear in any best rule set . . . 5

4 Core Rule Set **6**
4.1 Table of core rule set rules . . . 6
4.2 Event breakdown of core rule set . . . 7
4.3 Core Rule Set Penalties: Before and After Assignment . . . 8

5 Generalized Core Analysis **9**

6 Combined Core Table **13**

7 Dictionary of Copy Number Events **14**

1 Dataset Overview and Parameters

Number of samples = 120.
Number of events = 67.
Rule coverage requirement = 5 samples.
Rule assignment requirement = 4 samples.
Rule library size = 1260 rules.

1.1 Parameter table

Parameter Name	Value	Description	Category
n.cores	20	Number HPC Cores	Resources
msa	4	Minimum Samples Assigned	RS Constraint
rule.thresh	0.0417	Rule Coverage Thresh	Library Definition
max.rl	2000	Max Rule Library Size	Library Definition
p1.ntpr	40	P1 Num Trial Per Rule	Phase 1
p1.stop	24	P1 Stop Elimination	Phase 1
cut.size	0.25	P1 Cut Size	Phase 1
k.max.2	10	P2 Max K	Phase 2
max.nrs.p2	200000	P2 Max RS Evaluated Per K	Phase 2
Total Time	84.4	P2 Max RS Family Check Per K	Phase 2
max.stored.p2	10	P2 Num Top RS Stored Per K	Phase 2
max.nrs.p3	200000	P3 Max RS Evaluated Per K	Phase 3
max.stored.p3	100	P3 Num Top RS Stored Per K	Phase 3
k.max.4	40	P4 Max K	Phase 4
max.nrs.p4	100000	P4 Max RS Evaluated	Phase 4
max.stored.p4	100	P4 Num Top RS Stored Per K	Phase 4
gc.iter	100	Num CG Iterations	Generalized Core
gc.eval	100	Num RS Per GC	Generalized Core
r.seed	111	Total Computational Time	Timing

1.2 Timing breakdown (in minutes)

P1	PS	P2	P3	P4	GC	Total
4.07	5.95	6.95	11.9	20	35.5	84.4

2 Heatmaps of D and P

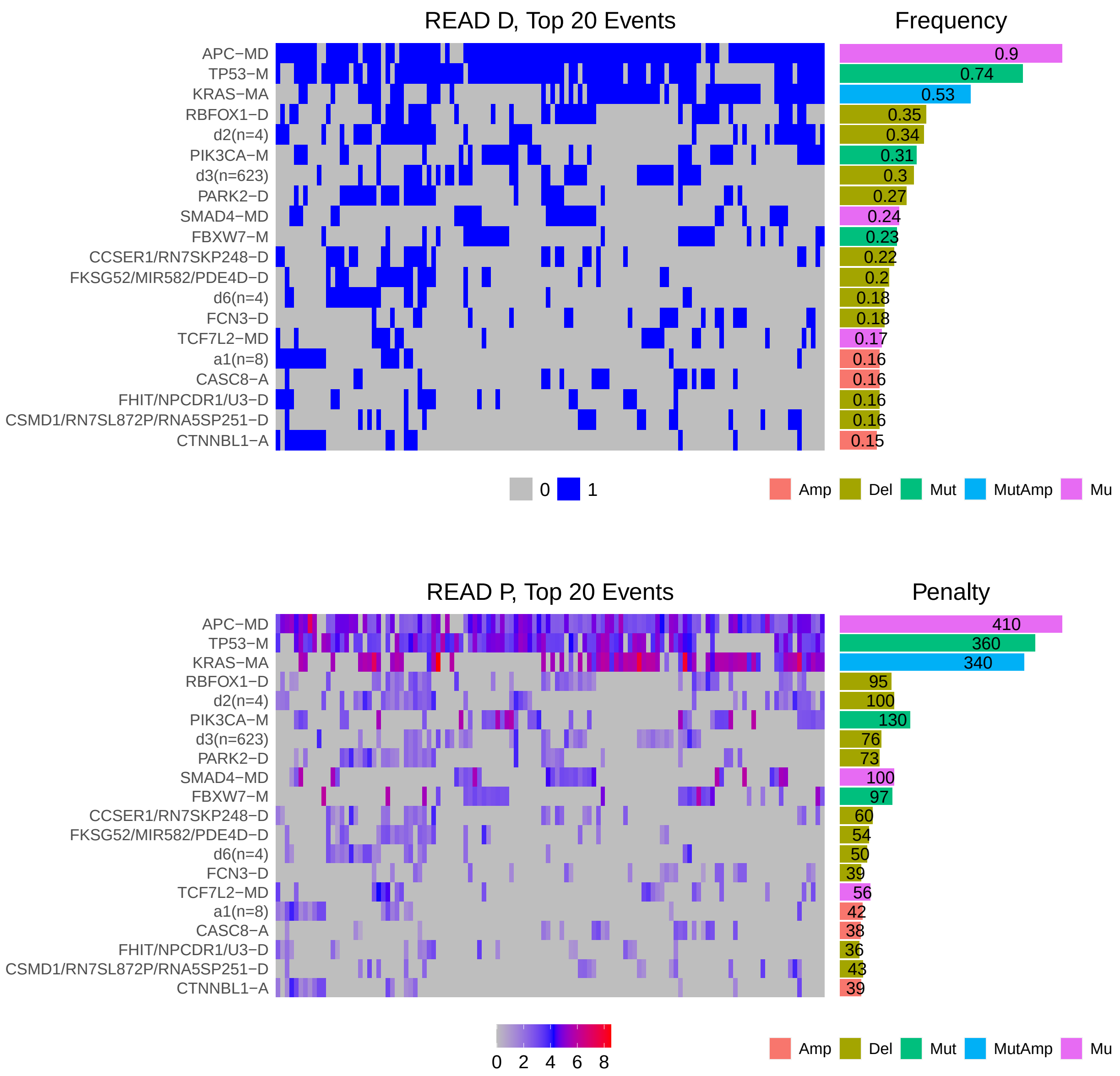

Figure 1: Heatmap of D and P. Top: binary representation of top 20 most frequent events. Top right shows event types and frequencies across the population. Bottom: Penalty matrix for top 20 most frequent events. Bottom right shows total event penalties. Events are ordered by frequency, from top to bottom. Samples are ordered using hierarchical clustering. Wild-type events indicated in grey. Event suffix -M: mutation, -A: amplification, -D: deletion, -MD: mutDel, -MA: mutAmp.

3 Summary of K Best Rule Sets

3.1 Performance and coverage of best rule sets

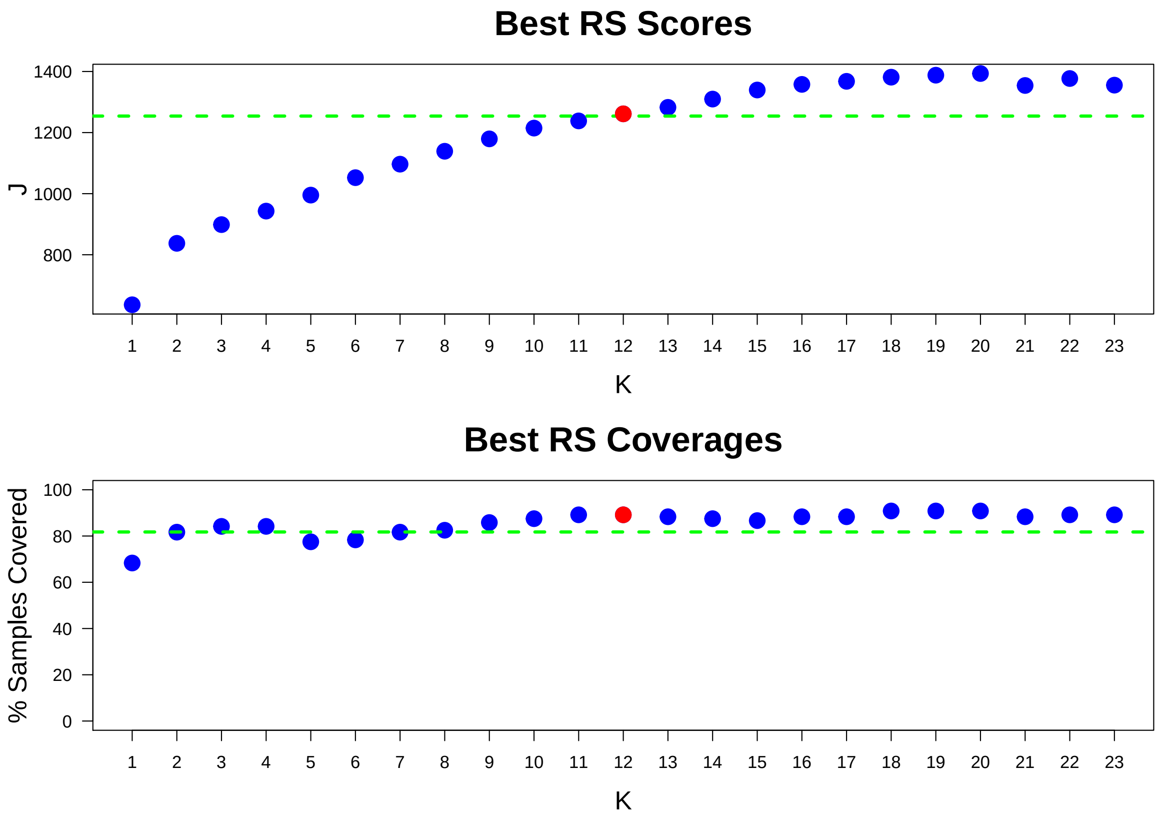

Figure 2: Performance and Coverage of Best Filtered RS for K. The core was determined to be the smallest RS that achieved at least 90% of maximum coverage and at least 90% of maximum performance.

3.2 Table of rules that appear in any best rule set

IR	Rule	PC	Ks
13	APC-MD+INS/MIR4686/TH-A+KRAS-MA	5.8	5-23
3	APC-MD+KRAS-MA+TP53-M	36	5-20
16	a1(n=8)+APC-MD+CTNNBL1-A+TP53-M	8.3	7-13,16-23
56	KRAS-MA+PIK3CA-M	17	9-23
192	d2(n=4)+KRAS-MA+PARK2-D+TP53-M	4.2	10-23
17	APC-MD+NRAS-M	9.2	11-23
67	APC-MD+d18(n=11)+PIK3CA-M+TP53-M	6.7	13-23
9	APC-MD+FBXW7-M+PIK3CA-M+TP53-M	12	13-22
10	APC-MD+d3(n=623)+TP53-M	22	7-13,15-17
20	APC-MD+d2(n=4)+KRAS-MA	16	14-23
91	APC-MD+d2(n=4)+GMDS-D+TP53-M	5.8	14-23
6	APC-MD+d2(n=4)	31	5-13
12	APC-MD+SMAD4-MD	22	5-13
82	APC-MD+CCSER1/RN7SKP248-D+d2(n=4)+d6(n=4)+FKSG52/MIR582/PDE4D-D+PARK2-D+TP53-M	4.2	14-16,18-23
95	APC-MD+KRAS-MA+RBFOX1-D+SMAD4-MD	6.7	15-23
4	APC-MD+PIK3CA-M+TP53-M	26	5-12
78	APC-MD+CCSER1/RN7SKP248-D+PARK2-D+RBFOX1-D+TP53-M	7.5	12-13,18-23
92	APC-MD+FHIT/NPCDR1/U3-D	16	16-23
29	APC-MD+d3(n=623)	27	18-23
30	APC-MD+TP53-M+USP12-A	9.2	18-23
138	APC-MD+CCSER1/RN7SKP248-D+d6(n=4)+FKSG52/MIR582/PDE4D-D+TP53-M	5.8	8-13
59	SMAD4-MD+TP53-M	18	15-19
60	APC-MD+CCSER1/RN7SKP248-D+RBFOX1-D+TP53-M	12	14-17
1	APC-MD+TP53-M	68	1-4
137	a4(n=7)+KRAS-MA+TP53-M+USP12-A	4.2	20-23
390	APC-MD+KRAS-MA+RBFOX1-D+TCF7L2-MD	4.2	20-23
2	APC-MD+KRAS-MA	49	2-4
24	APC-MD+CASC8-A+KRAS-MA+TP53-M	8.3	21-23
38	d3(n=623)+KRAS-MA+TP53-M	12	21-23
77	APC-MD+CSMD1/RN7SL872P/RNA5SP251-D+KRAS-MA+TP53-M	6.7	21-23
5	KRAS-MA+TP53-M	38	3-4
34	a1(n=8)+APC-MD+CTNNBL1-A	11	14-15
44	APC-MD+RBFOX1-D+SMAD4-MD+TP53-M	9.2	22-23
104	KRAS-MA+SMAD4-MD+TP53-M	8.3	22-23
122	APC-MD+CCSER1/RN7SKP248-D+SMAD4-MD+TP53-M	5.8	20-21
198	KRAS-MA+RBFOX1-D+TCF7L2-MD+TP53-M	5	18-19
7	APC-MD+CCSER1/RN7SKP248-D+d2(n=4)+FKSG52/MIR582/PDE4D-D+PARK2-D+TP53-M	5.8	17
15	APC-MD+SMAD4-MD+TP53-M	16	14
21	APC-MD+FBXW7-M+TP53-M	18	23
49	d3(n=623)+TP53-M	24	20
70	FBXW7-M+PIK3CA-M+TP53-M	12	23
128	AGBL4/BEND5/snoU13-D+APC-MD+d6(n=4)+IMMP2L/LRRN3/snoU13-D+RBFOX1-D+TP53-M	4.2	17
187	APC-MD+CASC8-A+FBXW7-M+KRAS-MA	5	19
566	CCSER1/RN7SKP248-D+d2(n=4)+FKSG52/MIR582/PDE4D-D+PARK2-D+TP53-M	5.8	4

ID = Rule IDs, rules are numbered according to importance rank determined from phase 1
PC = Percent of samples covered **Ks** = Membership in best RS

4 Core Rule Set

Core K = 12.
Core rule set coverage = 89.2%.

4.1 Table of core rule set rules

IR	Rule	CR	SJR	SJ	NSC	NSA	PC	PA	FracA
3	APC-MD + KRAS-MA + TP53-M	4	2	553	43	36	36	30.0	0.84
4	APC-MD + PIK3CA-M + TP53-M	11.5	5	339	31	14	26	12.0	0.45
6	APC-MD + d2(n=4)	6	14.5	230	37	9	31	7.5	0.24
10	APC-MD + d3(n=623) + TP53-M	18.5	8	251	26	6	22	5.0	0.23
12	APC-MD + SMAD4-MD	18.5	28	184	26	6	22	5.0	0.23
13	APC-MD + INS/MIR4686/TH-A + KRAS-MA	423.5	240	83.5	7	5	5.8	4.2	0.71
16	a1(n=8) + APC-MD + CTNNBL1-A + TP53-M	183	89	125	10	4	8.3	3.3	0.40
17	APC-MD + NRAS-M	148.5	156	98.7	11	4	9.2	3.3	0.36
56	KRAS-MA + PIK3CA-M	33	36	171	20	5	17	4.2	0.25
78	APC-MD + CCSER1/RN7SKP248-D + PARK2-D + RBFOX1-D + TP53-M	237	93	123	9	8	7.5	6.7	0.89
138	APC-MD + CCSER1/RN7SKP248-D + d6(n=4) + FKSG52/MIR582/PDE4D-D + TP53-M	423.5	163.5	96.4	7	5	5.8	4.2	0.71
192	d2(n=4) + KRAS-MA + PARK2-D + TP53-M	998	339	72.9	5	5	4.2	4.2	1.00

ID = Rule IDs, rules are numbered according to importance rank determined from phase 1
CR = Coverage rank
SJ/SJR = Single rule performance / performance rank
NSC/PC = Number/percent of samples covered
NSA/PA = Number/percent of samples assigned
FracA = Fraction of covered samples assigned to rule

4.2 Event breakdown of core rule set

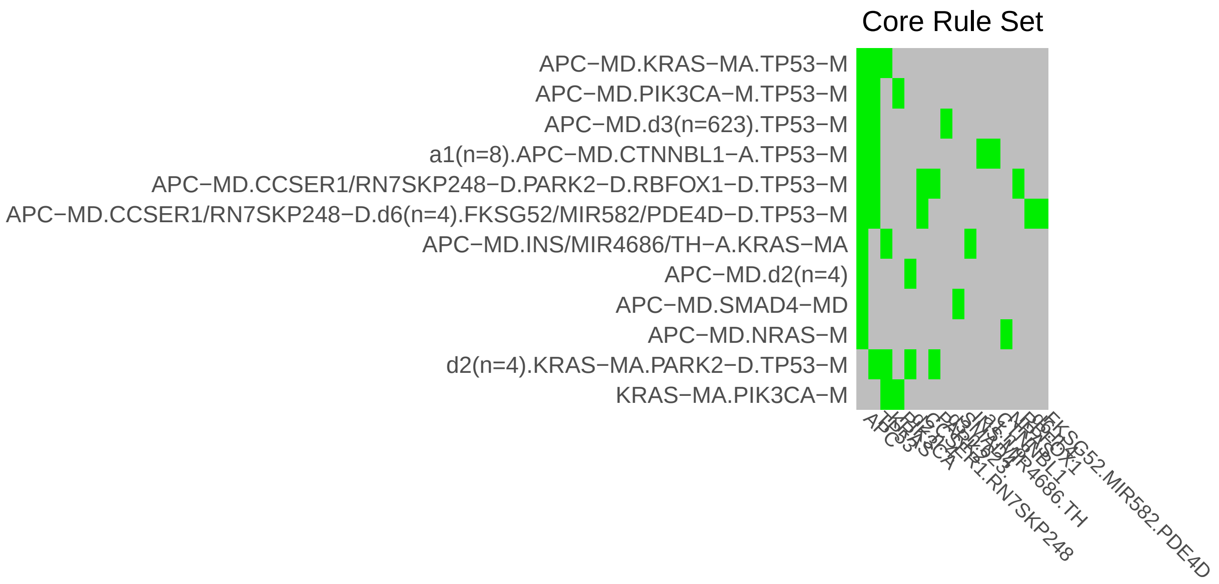

Figure 3: Visualization of core rule set. Rows are rules, columns are events. Events are ordered from left in decreasing rule membership frequency.

4.3 Core Rule Set Penalties: Before and After Assignment

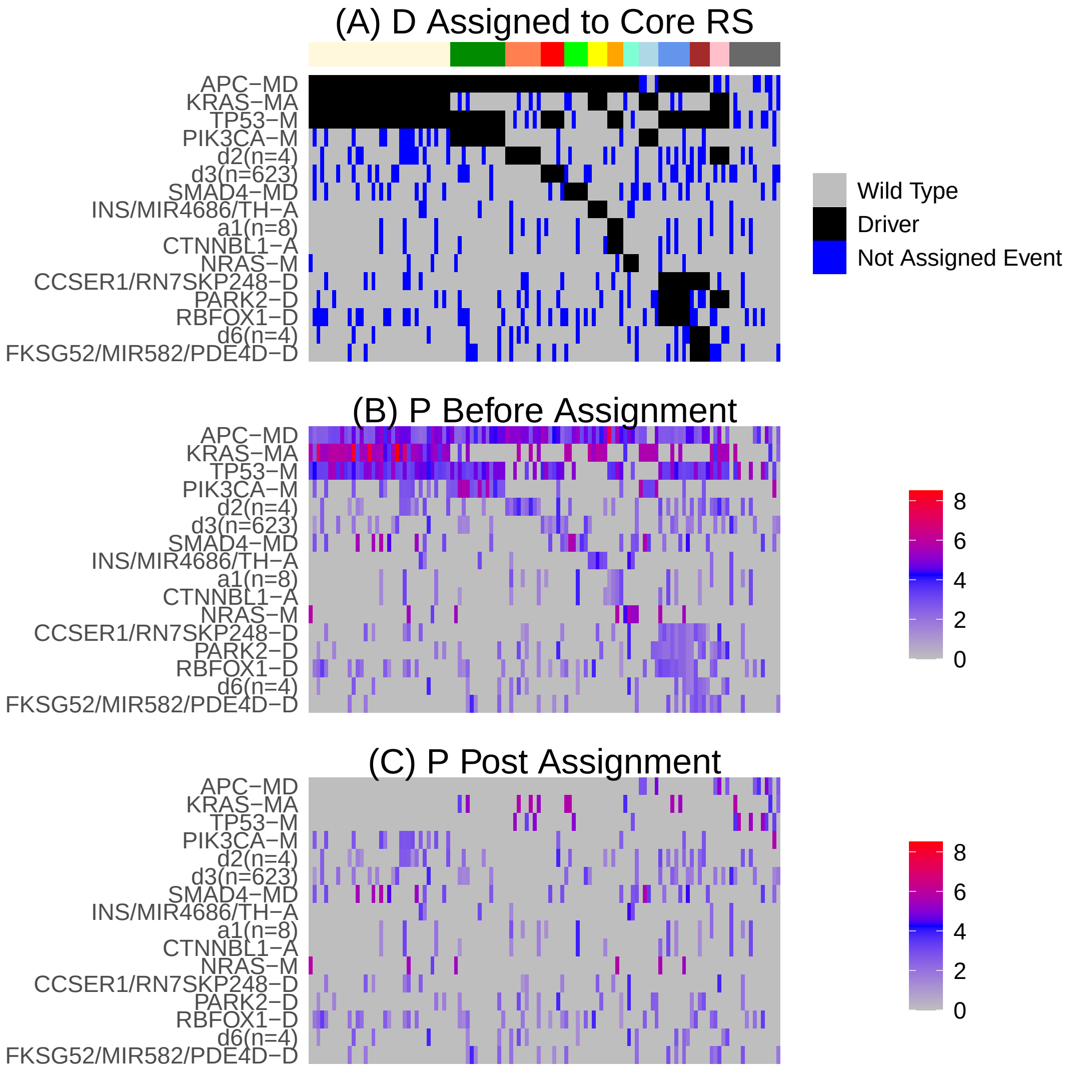

Figure 4: Heatmaps of D and P under core rule set assignment. Events are ordered by frequency. Samples are ordered according rule set membership, as indicated by the color bar. The dark grey group of samples are not assigned to any rule. A) For each sample, assigned events are designated as drivers and are shown in black, unassigned events are shown in blue. B-C) Heatmap of P before and after assignment to core rule set.

5 Generalized Core Analysis

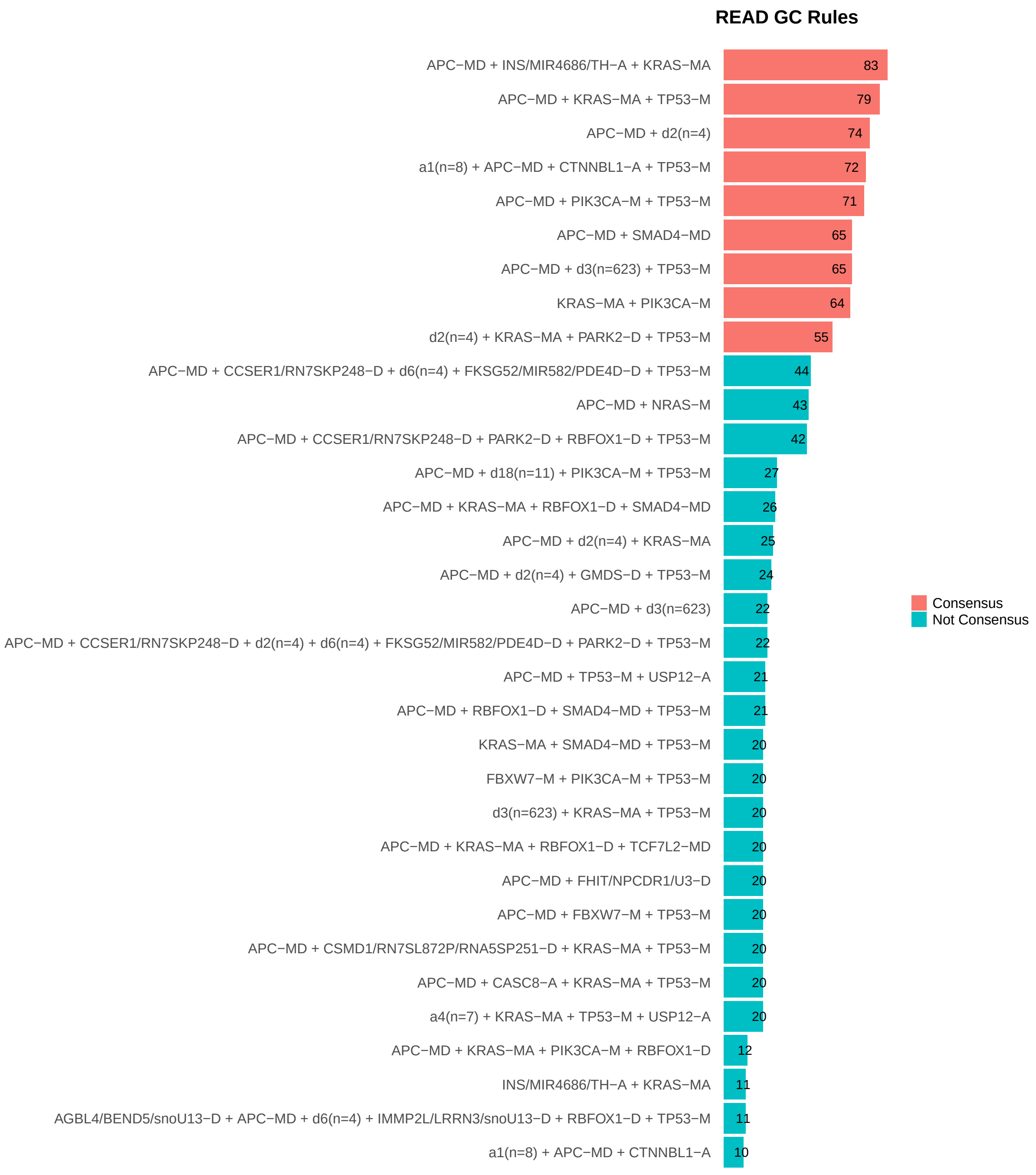

Figure 5: GCRs

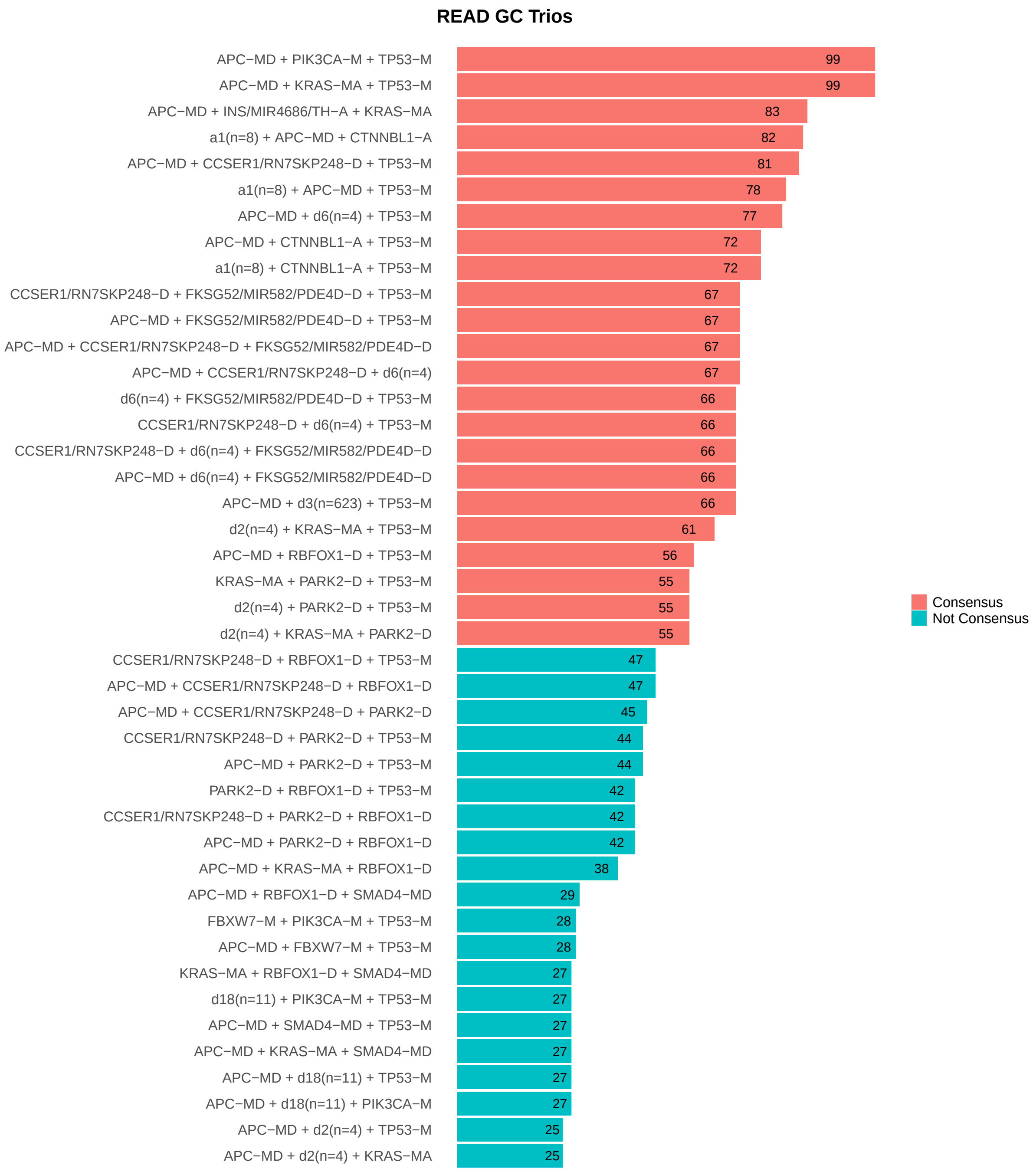

Figure 6: GCTs

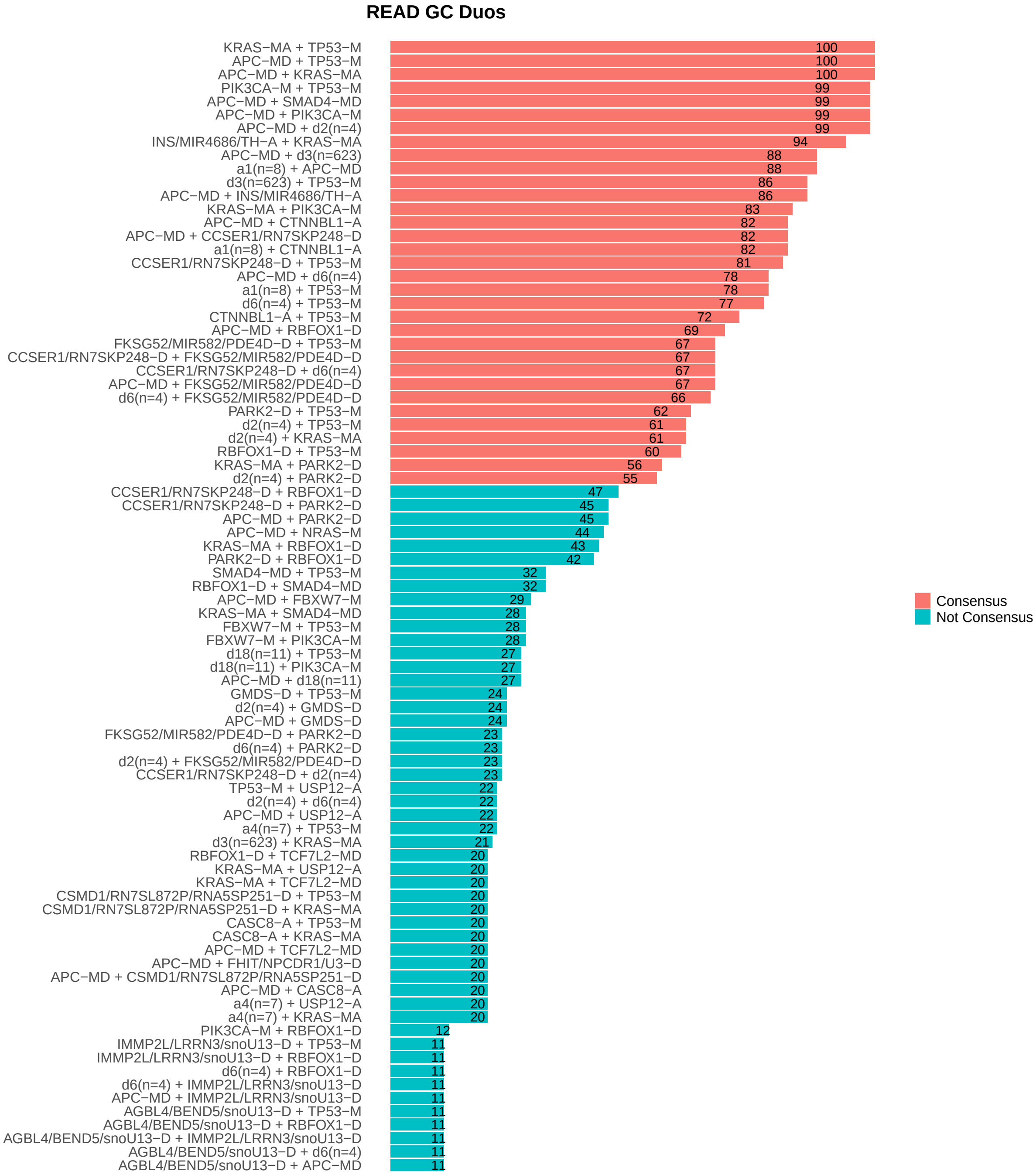

Figure 7: GCDs

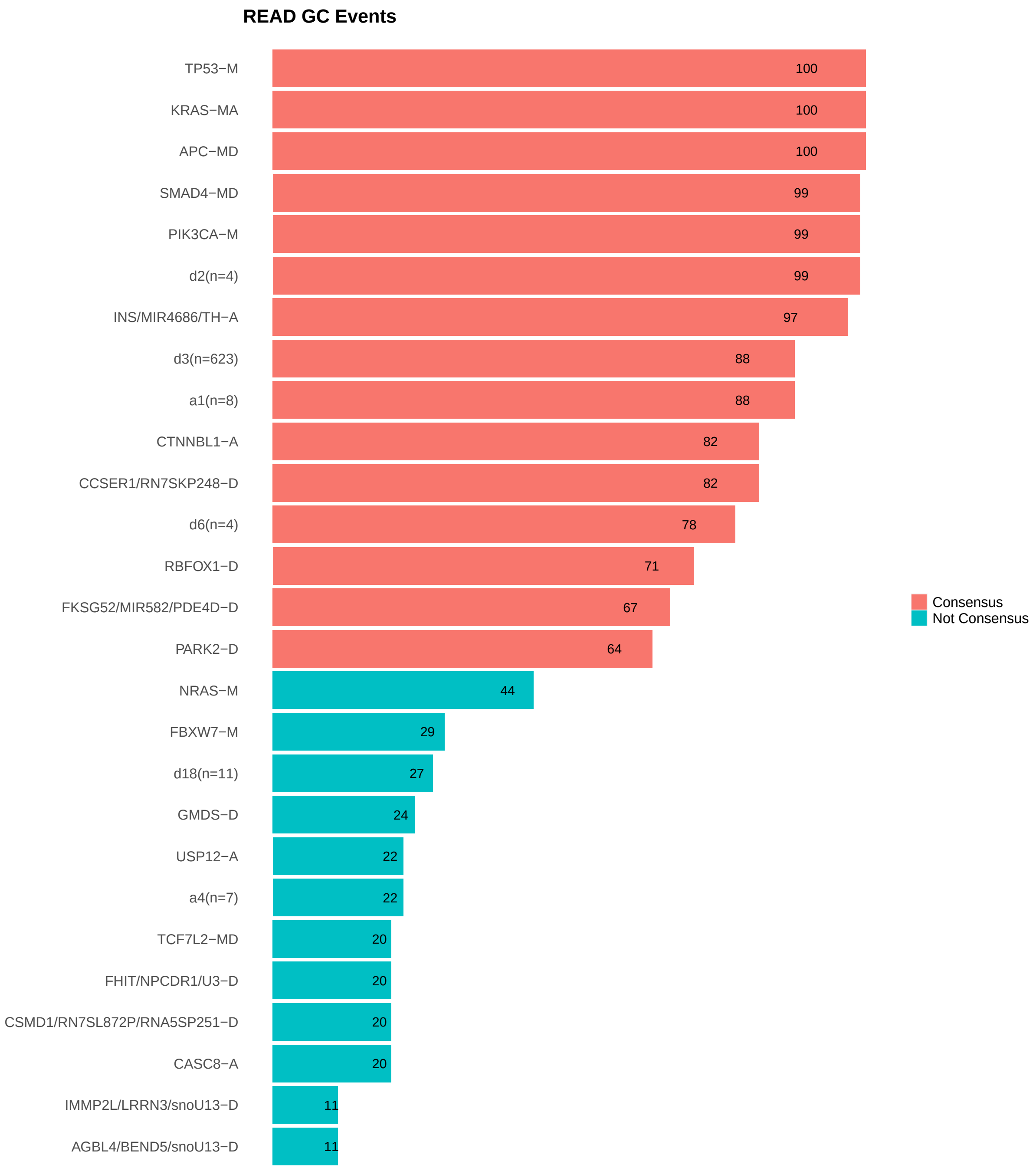

Figure 8: GCEs

6 Combined Core Table

Rule	Core_Type	P1_Rank	Confidence	Coverage	SJ	Fraction_Assigned
APC-MD + INS/MIR4686/TH-A + KRAS-MA	Both	13	83	5.83% (r=459)	231 (r=240)	0.71
APC-MD + KRAS-MA + TP53-M	Both	3	79	35.8% (r=4)	521 (r=2)	0.67
APC-MD + d2(n=4)	Both	6	74	30.8% (r=6)	278 (r=14)	0.41
a1(n=8) + APC-MD + CTNNBL1-A + TP53-M	Both	16	72	8.33% (r=194)	221 (r=88)	0.30
APC-MD + PIK3CA-M + TP53-M	Both	4	71	25.8% (r=12)	430 (r=5)	0.32
APC-MD + d3(n=623) + TP53-M	Both	10	65	21.7% (r=20)	239 (r=8)	0.12
APC-MD + SMAD4-MD	Both	12	65	21.7% (r=19)	237 (r=28)	0.65
KRAS-MA + PIK3CA-M	Both	56	64	16.7% (r=33)	147 (r=36)	0.55
d2(n=4) + KRAS-MA + PARK2-D + TP53-M	Both	192	55	4.17% (r=1011)	90.9 (r=339)	0.60
APC-MD + CCSER1/RN7SKP248-D + d6(n=4) + FKSG52/MIR582/PDE4D-D + TP53-M	Core	138	44	5.83% (r=472)	104 (r=163)	0.14
APC-MD + NRAS-M	Core	17	43	9.17% (r=148)	221 (r=156)	0.91
APC-MD + CCSER1/RN7SKP248-D + PARK2-D + RBFOX1-D + TP53-M	Core	78	42	7.5% (r=269)	130 (r=92)	0.00

7 Dictionary of Copy Number Events

CNV	Genes	Event_Name
a1	HCK, PLAGL2, KIF3B, TM9SF4, POFUT1, TSPY26P, ASXL1, MIR1825	a1(n=8)
a2	CTNNBL1	CTNNBL1-A
a3	CASC8	CASC8-A
a4	RN7SL272P, URAD, CDX2, LINC00543, FLT3, PDX1, ATP5EP2	a4(n=7)
a5	USP12	USP12-A
a8	INS, TH, MIR4686	INS/MIR4686/TH-A
d1	RBFOX1	RBFOX1-D
d2	RNA5SP475, RN7SL864P, FLRT3, MACROD2	d2(n=4)
d3	d3(n=623)	d3(n=623)
d4	PARK2	PARK2-D
d5	RN7SKP248, CCSER1	CCSER1/RN7SKP248-D
d6	snoU13|ENSG00000271842.1, MIR4789, RN7SKP40, NAALADL2	d6(n=4)
d7	FCN3	FCN3-D
d8	FKSG52, MIR582, PDE4D	FKSG52/MIR582/PDE4D-D
d9	U3|ENSG00000212211.1, NPCDR1, FHIT	FHIT/NPCDR1/U3-D
d10	RN7SL872P, RNA5SP251, CSMD1	CSMD1/RN7SL872P/RNA5SP251-D
d11	snoU13|ENSG00000239144.1, BEND5, AGBL4	AGBL4/BEND5/snoU13-D
d15	GMDS	GMDS-D
d16	snoU13|ENSG00000238922.1, LRRN3, IMMP2L	IMMP2L/LRRN3/snoU13-D
d18	ANKRD20A11P, CYP4F29P, ANKRD30BP2, BAGE2, SNORA70|ENSG00000252199.1, RN7SL52P, TEKT4P2, MIR3648, TPTE, POTED, MIR3687	d18(n=11)

Dictionary of important CNVs. Copy number events that are within any of the best K rule sets, or within the generalized core rules, or among the 20 most frequent events.
