# Supplementary material for: Identifying modules of cooperating cancer drivers
Source: Mol Syst Biol. 2021 Mar 26;17(3):e9810. doi: 10.15252/msb.20209810 (PMC7995435; doi:10.15252/msb.20209810)
Supplement: Supplementary file 18 — Dataset EV17 [file MSB-17-e9810-s005.zip › DATASET EV17/CRSO_Report_SKCM.pdf]

CRSO Output Report: SKCM

Michael Klein

Nov 11 2020

Contents

1 Dataset Overview and Parameters **2**
1.1 Parameter table 2
1.2 Timing breakdown (in minutes) 2

2 Heatmaps of D and P **3**

3 Summary of K Best Rule Sets **4**
3.1 Performance and coverage of best rule sets 4
3.2 Table of rules that appear in any best rule set 5

4 Core Rule Set **6**
4.1 Table of core rule set rules 6
4.2 Event breakdown of core rule set 7
4.3 Core Rule Set Penalties: Before and After Assignment 8

5 Generalized Core Analysis **9**

6 Combined Core Table **13**

7 Dictionary of Copy Number Events **14**

1 Dataset Overview and Parameters

Number of samples = 290.
Number of events = 71.
Rule coverage requirement = 9 samples.
Rule assignment requirement = 9 samples.
Rule library size = 197 rules.

1.1 Parameter table

**Parameter Name**	**Value**	**Description**	**Category**
n.cores	20	Number HPC Cores	Resources
msa	9	Minimum Samples Assigned	RS Constraint
rule.thresh	0.031	Rule Coverage Thresh	Library Definition
max.rl	2000	Max Rule Library Size	Library Definition
p1.ntpr	40	P1 Num Trial Per Rule	Phase 1
p1.stop	24	P1 Stop Elimination	Phase 1
cut.size	0.25	P1 Cut Size	Phase 1
k.max.2	10	P2 Max K	Phase 2
max.nrs.p2	200000	P2 Max RS Evaluated Per K	Phase 2
Total Time	53.5	P2 Max RS Family Check Per K	Phase 2
max.stored.p2	10	P2 Num Top RS Stored Per K	Phase 2
max.nrs.p3	200000	P3 Max RS Evaluated Per K	Phase 3
max.stored.p3	100	P3 Num Top RS Stored Per K	Phase 3
k.max.4	40	P4 Max K	Phase 4
max.nrs.p4	100000	P4 Max RS Evaluated	Phase 4
max.stored.p4	100	P4 Num Top RS Stored Per K	Phase 4
gc.iter	100	Num CG Iterations	Generalized Core
gc.eval	100	Num RS Per GC	Generalized Core
r.seed	111	Total Computational Time	Timing

1.2 Timing breakdown (in minutes)

P1	PS	P2	P3	P4	GC	Total
1.48	8.44	4.03	8.79	6.06	24.7	53.5

2 Heatmaps of D and P

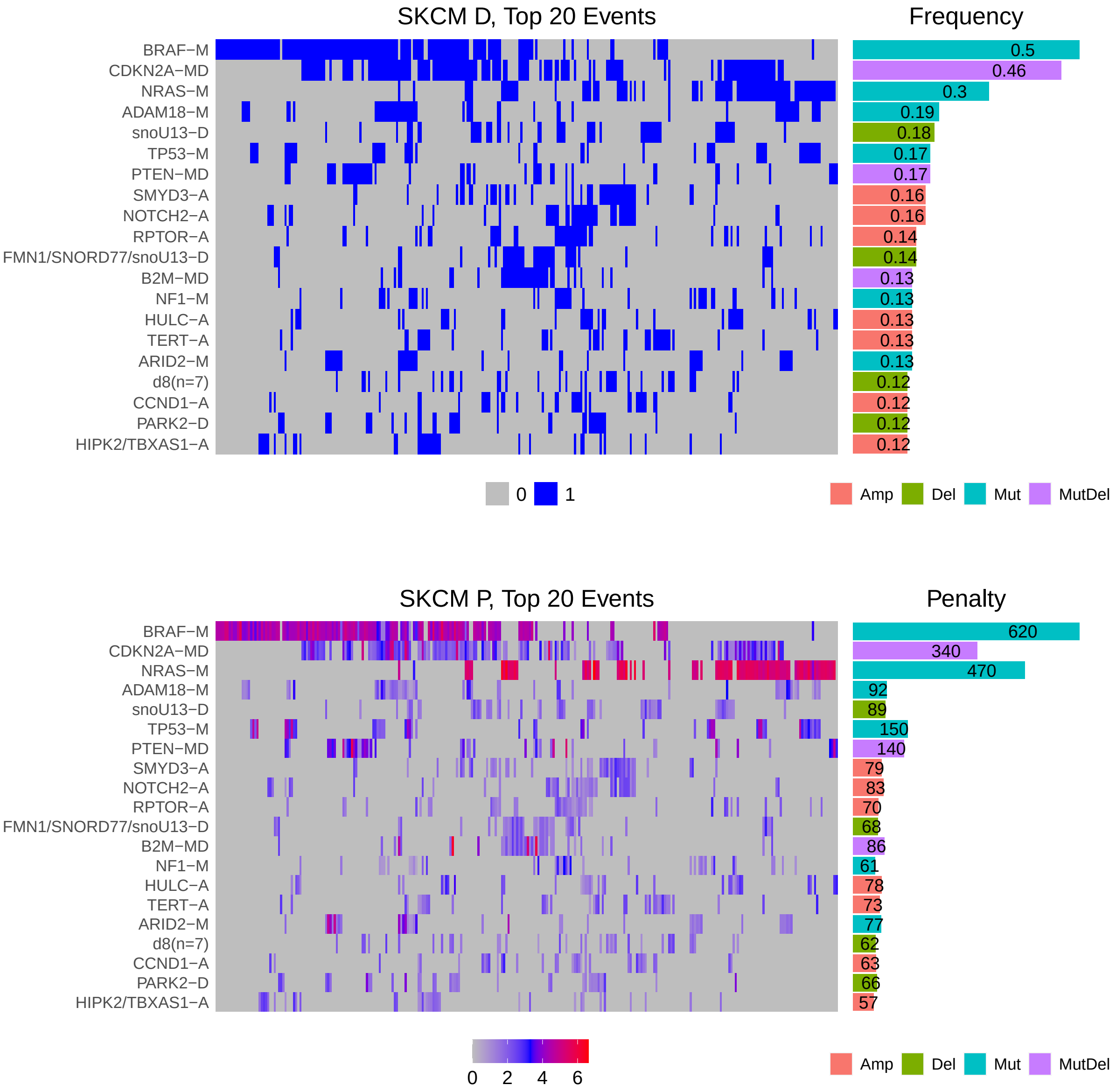

Figure 1: Heatmap of D and P. Top: binary representation of top 20 most frequent events. Top right shows event types and frequencies across the population. Bottom: Penalty matrix for top 20 most frequent events. Bottom right shows total event penalties. Events are ordered by frequency, from top to bottom. Samples are ordered using hierarchical clustering. Wild-type events indicated in grey. Event suffix -M: mutation, -A: amplification, -D: deletion, -MD: mutDel, -MA: mutAmp.

3 Summary of K Best Rule Sets

3.1 Performance and coverage of best rule sets

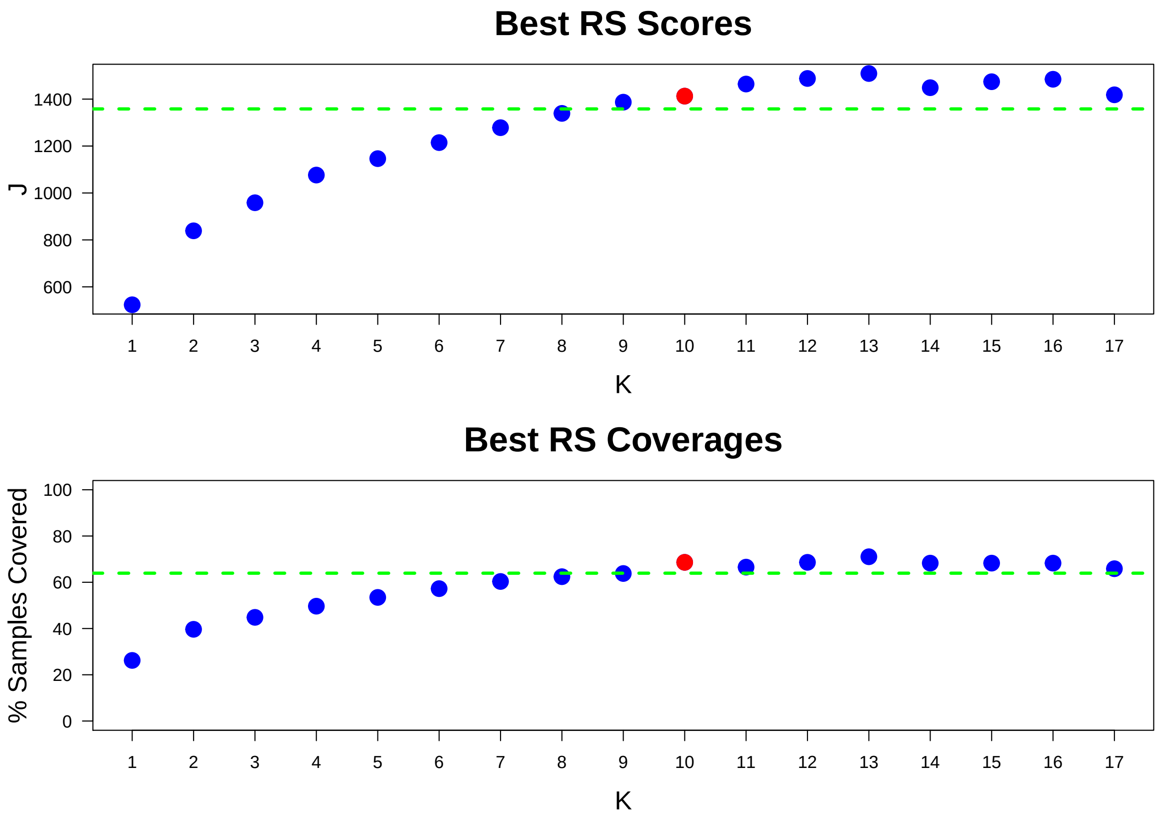

Figure 2: Performance and Coverage of Best Filtered RS for K. The core was determined to be the smallest RS that achieved at least 90% of maximum coverage and at least 90% of maximum performance.

3.2 Table of rules that appear in any best rule set

IR	Rule	PC	Ks
1	BRAF-M+CDKN2A-MD	26	1-14
2	CDKN2A-MD+NRAS-M	14	2-13,15-16
4	NRAS-M+TP53-M	6.6	4-17
3	BRAF-M+PTEN-MD	11	3-15
6	BRAF-M+HIPK2/TBXAS1-A	7.9	5-17
11	BRAF-M+RN7SKP254-A	6.6	6-17
21	ARID2-M+NRAS-M	5.2	7-9,11-12,14-16
32	KCNN3-A+NOTCH2-A+NRAS-M	3.4	8-9,11-16
5	HULC-A+NRAS-M	6.6	10-13,15-17
8	BRAF-M+TP53-M	8.3	11-17
28	B2M-MD+FMN1/SNORD77/snoU13-D+NRAS-M	3.4	11-14
10	B2M-MD+FMN1/SNORD77/snoU13-D	9	15-17
23	CDKN2A-MD+NF1-M	6.6	14-17
9	ADAM18-M+NRAS-M	6.9	
12	BRAF-M+CDKN2A-MD+PARK2-D	5.2	15-17
16	ADAM18-M+BRAF-M+CDKN2A-MD	6.9	15-17
18	BRAF-M+CDKN2A-MD+LINC00290-D	4.5	15-17
24	ARID2-M+BRAF-M	6.2	16-17
80	B2M-MD+CDKN2A-MD	7.6	12-14
7	BRAF-M+CDKN2A-MD+PTEN-MD	5.5	16-17
15	NRAS-M+SMYD3-A	5.5	
25	BRAF-M+d5(n=5)	6.9	16-17
59	CDKN2A-MD+NRAS-M+snoU13-D	3.1	
26	BRAF-M+CDKN2A-MD+d5(n=5)	5.2	15
29	NRAS-M+PTEN-MD	3.4	14
30	FMN1/SNORD77/snoU13-D+NRAS-M	4.8	17
117	d8(n=7)+SMYD3-A	4.8	13

ID = Rule IDs, rules are numbered according to importance rank determined from phase 1
PC = Percent of samples covered **Ks** = Membership in best RS

4 Core Rule Set

Core K = 10.
Core rule set coverage = 68.6%.

4.1 Table of core rule set rules

IR	Rule	CR	SJR	SJ	NSC	NSA	PC	PA	FracA
1	BRAF-M + CDKN2A-MD	1	1	524	76	65	26	22.0	0.86
2	CDKN2A-MD + NRAS-M	2	2	327	41	28	14	9.7	0.68
3	BRAF-M + PTEN-MD	3.5	3	227	31	21	11	7.2	0.68
4	NRAS-M + TP53-M	29	8	156	19	17	6.6	5.9	0.89
5	HULC-A + NRAS-M	29	9	146	19	11	6.6	3.8	0.58
6	BRAF-M + HIPK2/TBXAS1-A	11.5	10	144	23	12	7.9	4.1	0.52
9	ADAM18-M + NRAS-M	22.5	11	137	20	9	6.9	3.1	0.45
10	B2M-MD + FMN1/SNORD77/snoU13-D	6.5	36	96.4	26	14	9	4.8	0.54
11	BRAF-M + RN7SKP254-A	29	21	115	19	12	6.6	4.1	0.63
15	NRAS-M + SMYD3-A	42.5	20	119	16	10	5.5	3.4	0.62

ID = Rule IDs, rules are numbered according to importance rank determined from phase 1
CR = Coverage rank
SJ/SJR = Single rule performance / performance rank
NSC/PC = Number/percent of samples covered
NSA/PA = Number/percent of samples assigned
FracA = Fraction of covered samples assigned to rule

4.2 Event breakdown of core rule set

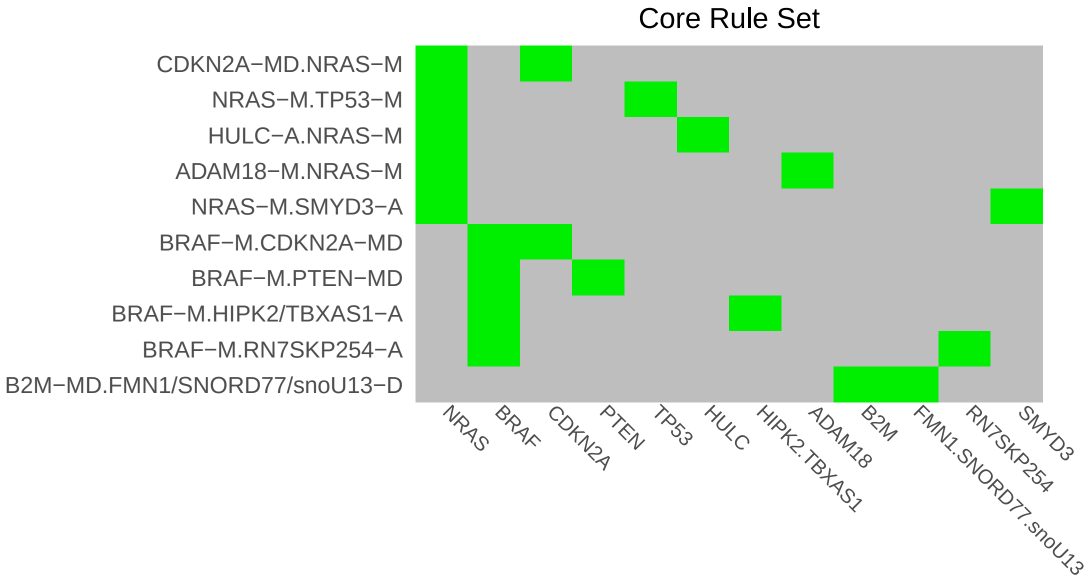

Figure 3: Visualization of core rule set. Rows are rules, columns are events. Events are ordered from left in decreasing rule membership frequency.

4.3 Core Rule Set Penalties: Before and After Assignment

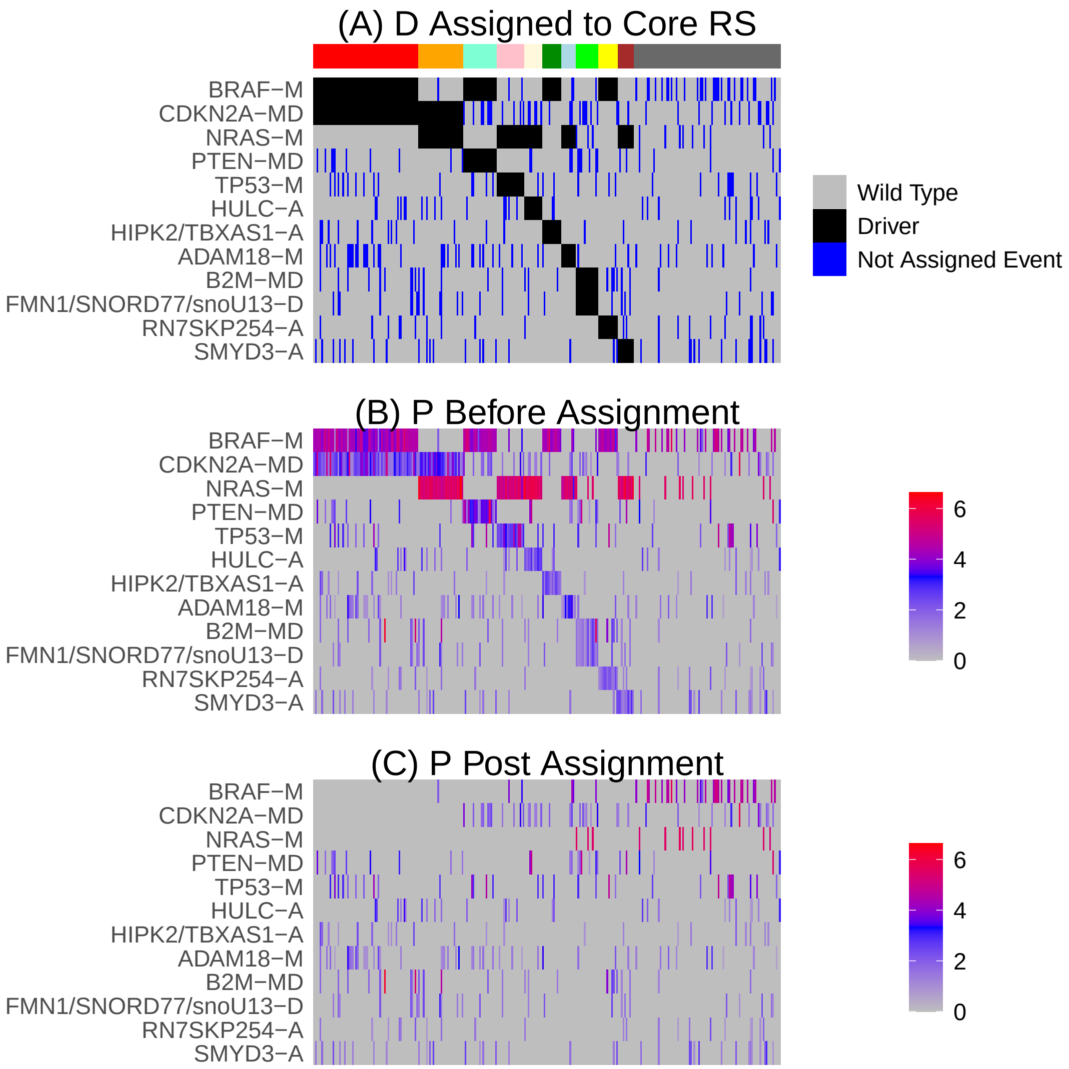

Figure 4: Heatmaps of D and P under core rule set assignment. Events are ordered by frequency. Samples are ordered according rule set membership, as indicated by the color bar. The dark grey group of samples are not assigned to any rule. A) For each sample, assigned events are designated as drivers and are shown in black, unassigned events are shown in blue. B-C) Heatmap of P before and after assignment to core rule set.

5 Generalized Core Analysis

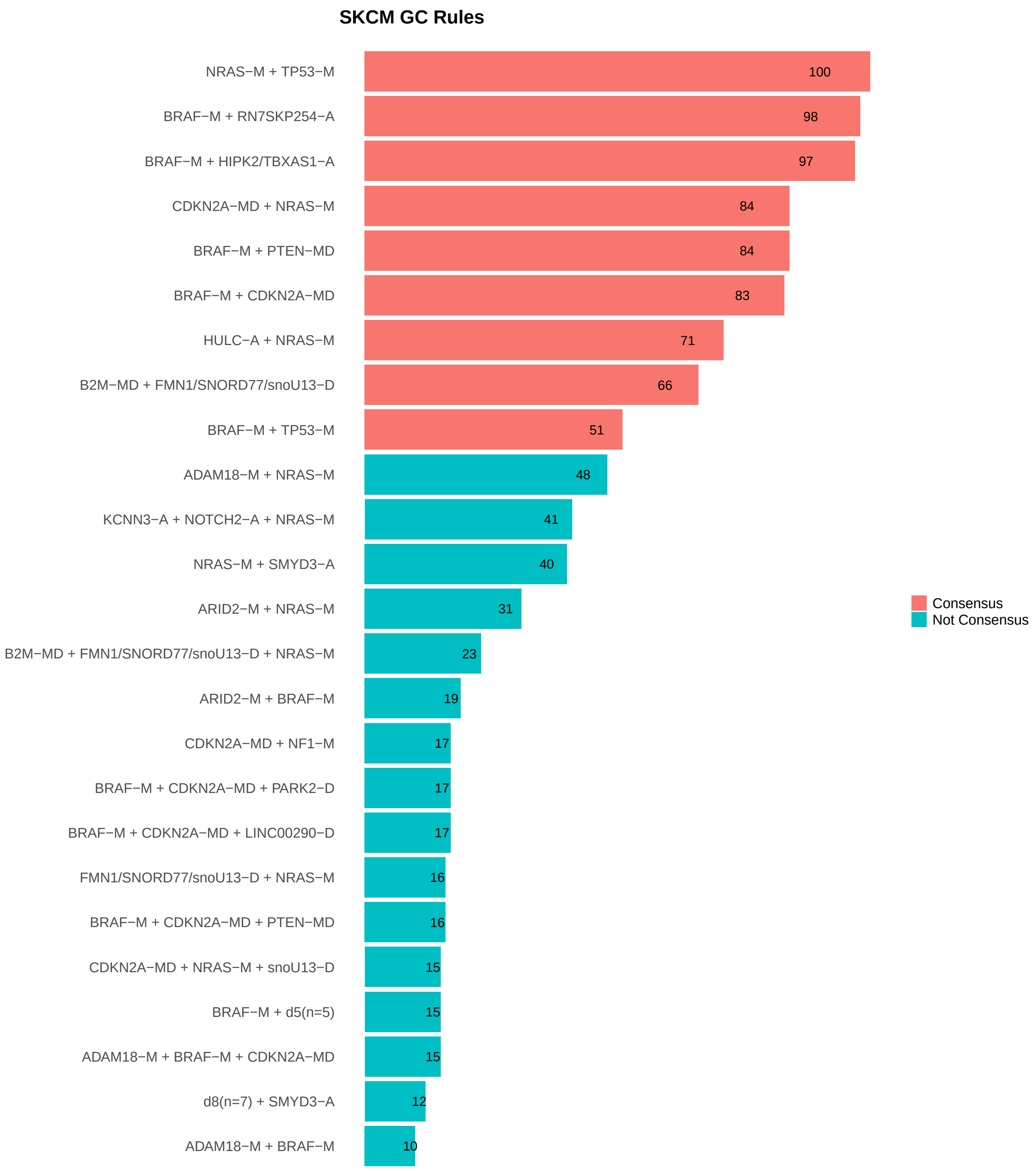

Figure 5: GCRs

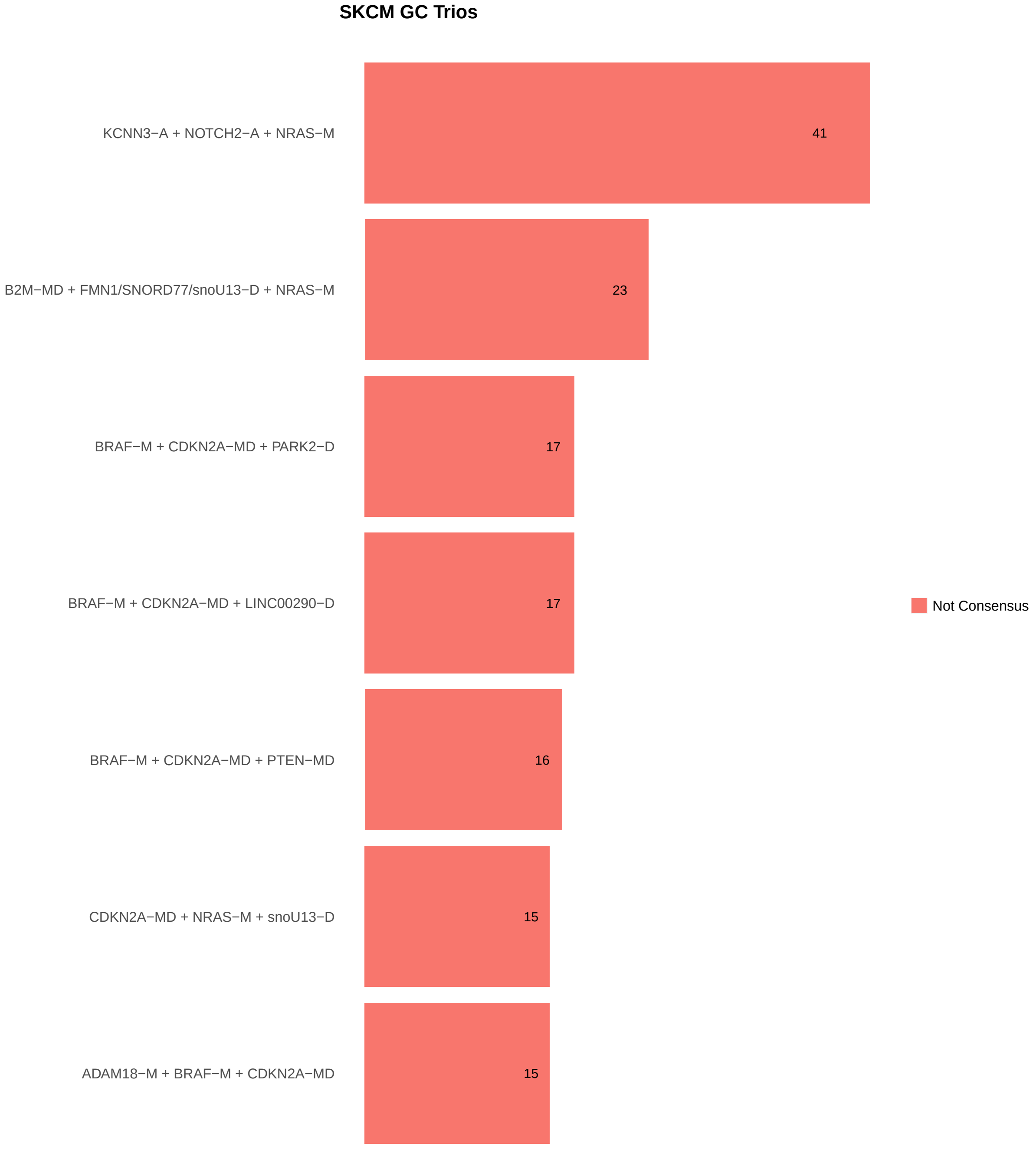

Figure 6: GCTs

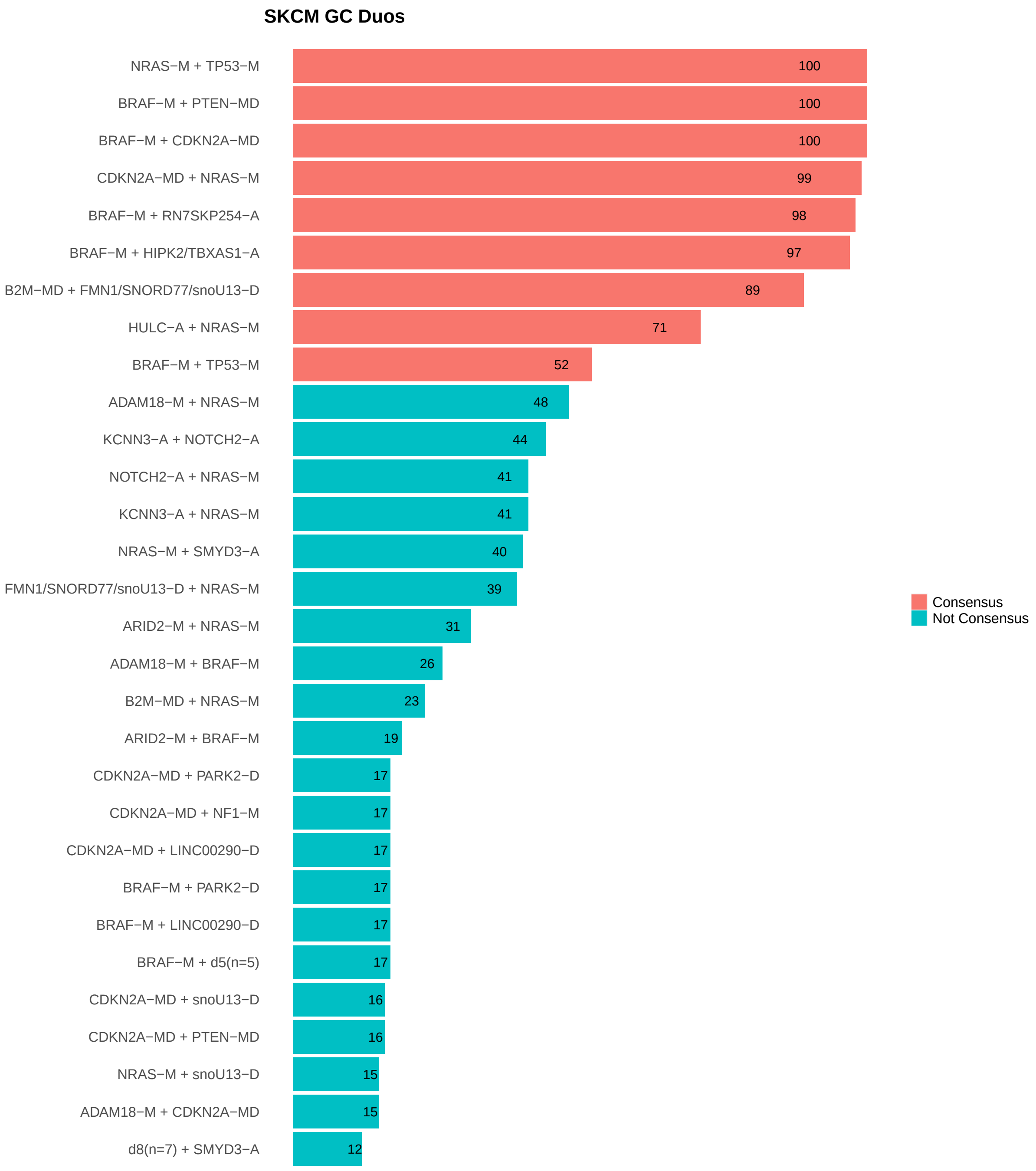

Figure 7: GCDs

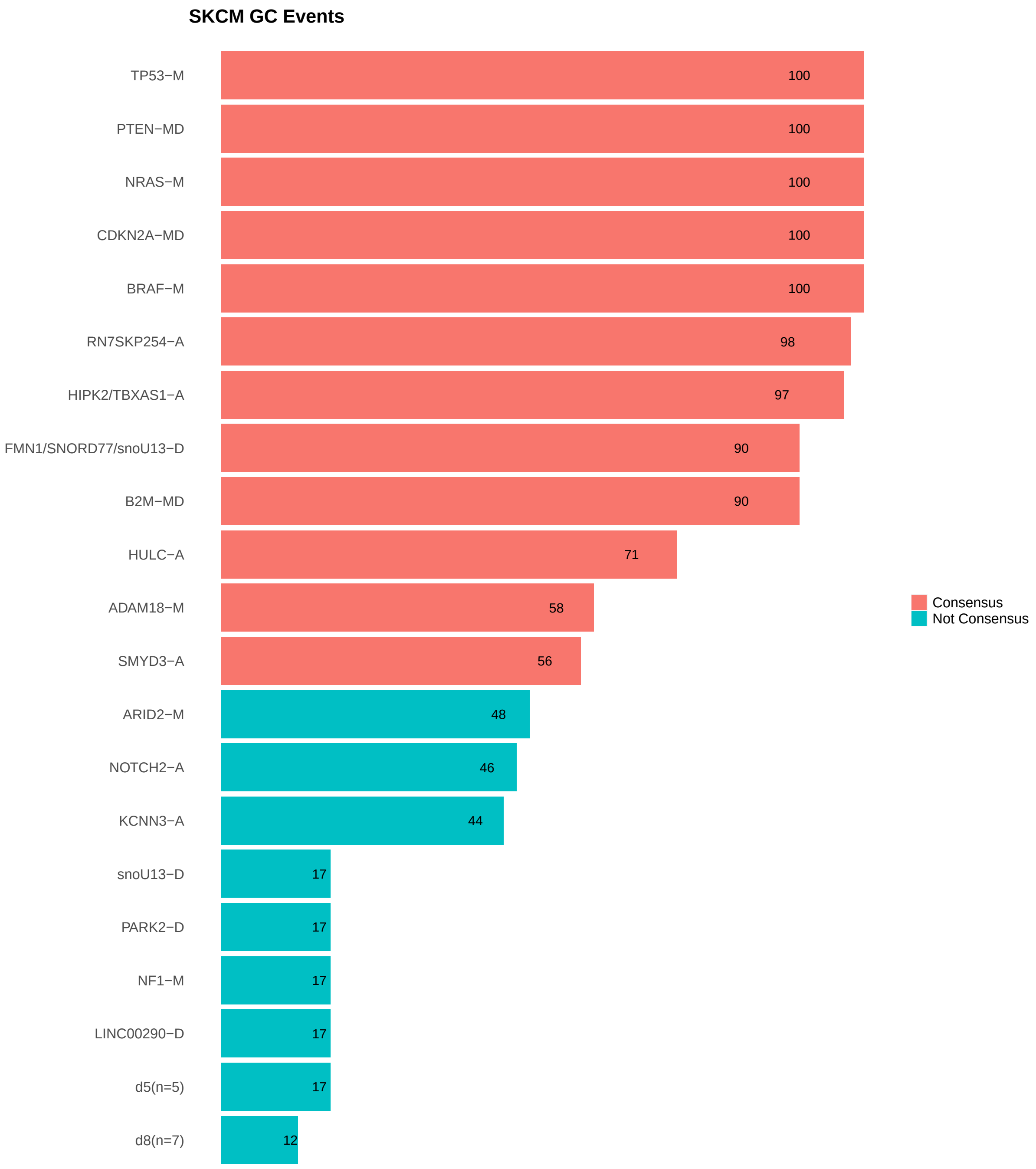

Figure 8: GCEs

6 Combined Core Table

Rule	Core_Type	P1_Rank	Confidence	Coverage	SJ	Fraction_Assigned
NRAS-M + TP53-M	Both	4	100	6.55% (r=29)	168 (r=8)	0.89
BRAF-M + RN7SKP254-A	Both	11	98	6.55% (r=32)	137 (r=21)	0.58
BRAF-M + HIPK2/TBXAS1-A	Both	6	97	7.93% (r=13)	161 (r=10)	0.52
CDKN2A-MD + NRAS-M	Both	2	84	14.1% (r=2)	327 (r=2)	0.68
BRAF-M + PTEN-MD	Both	3	84	10.7% (r=3)	227 (r=3)	0.71
BRAF-M + CDKN2A-MD	Both	1	83	26.2% (r=1)	524 (r=1)	0.83
HULC-A + NRAS-M	Both	5	71	6.55% (r=28)	167 (r=9)	0.58
B2M-MD + FMN1/SNORD77/snoU13-D	Both	10	66	8.97% (r=6)	144 (r=36)	0.62
ADAM18-M + NRAS-M	Core	9	48	6.9% (r=20)	146 (r=11)	0.45
NRAS-M + SMYD3-A	Core	15	40	5.52% (r=40)	131 (r=20)	0.62
BRAF-M + TP53-M	conGCR	8	51	8.28% (r=9)	156 (r=7)	-

7 Dictionary of Copy Number Events

CNV	Genes	Event_Name
a1	SMYD3	SMYD3-A
a2	RPTOR	RPTOR-A
a3	NOTCH2	NOTCH2-A
a4	HULC	HULC-A
a5	TBXAS1, HIPK2	HIPK2/TBXAS1-A
a6	CCND1	CCND1-A
a7	MITF	MITF-A
a8	TERT	TERT-A
a9	KCNN3	KCNN3-A
a10	RN7SKP254	RN7SKP254-A
d2	snoU13|ENSG00000239153.1	snoU13-D
d3	PARK2	PARK2-D
d5	SNORA51|ENSG00000207022.1, SNORA66|ENSG00000207523.1, SNORA66|ENSG00000251795.1, SNORD21, RPL5	d5(n=5)
d7	SNORD77|ENSG00000212415.1, snoU13|ENSG00000238342.1, FMN1	FMN1/SNORD77/snoU13-D
d8	RPL22, KCNAB2, CHD5, NPHP4, RNF207, MIR4689, MIR4417	d8(n=7)
d12	LINC00290	LINC00290-D
d14	PIH1, WWOX	PIH1/WWOX-D
d18	d18(n=27)	d18(n=27)

Dictionary of important CNVs. Copy number events that are within any of the best K rule sets, or within the generalized core rules, or among the 20 most frequent events.
